# Zwitterionic near infrared fluorescent agents for noninvasive real-time transcutaneous assessment of kidney function†
†Electronic supplementary information (ESI) available. See DOI: 10.1039/c6sc05059j



**DOI:** 10.1039/c6sc05059j

**Published:** 2017-01-11

**Authors:** Jiaguo Huang, Stefanie Weinfurter, Cristina Daniele, Rossana Perciaccante, Rodeghiero Federica, Leopoldo Della Ciana, Johannes Pill, Norbert Gretz

**Affiliations:** a Medical Research Center , Medical Faculty Mannheim , University of Heidelberg , Theodor-Kutzer-Ufer 1-3 , 68167 , Mannheim , Germany . Email: Norbert.Gretz@medma.uni-heidelberg.de; b Cyanagen S.r.l. , Via degli Stradelli Guelfi 40/C , 40138 Bologna , BO , Italy

## Abstract

We developed novel zwitterionic near infrared (NIR) fluorescent agents (ABZWCY-HPβCD and AAZWCY-HPβCD), which exhibit favorable hydrophilicity, low plasma protein binding, high stability and non-toxicity. These attractive characteristics ensure that they are excreted rapidly, without any skin accumulation or metabolism *in vivo*. More importantly, zwitterionic HPβCD based agents can be efficiently filtrated by the glomerulus and completely excreted through the kidneys into urine without reabsorption or secretion in the kidney proximal tubule. Relying on these novel zwitterionic NIR agents and a transcutaneous device, we demonstrate a rapid, robust and biocompatible approach for assessing kidney function in rat models of both healthy rats and those with kidney disease, without the need for time-consuming blood/urine sample preparation. Our work provides a promising tool for *in vivo* real-time non-invasive kidney function assessment in preclinical applications.

## Abstract 2

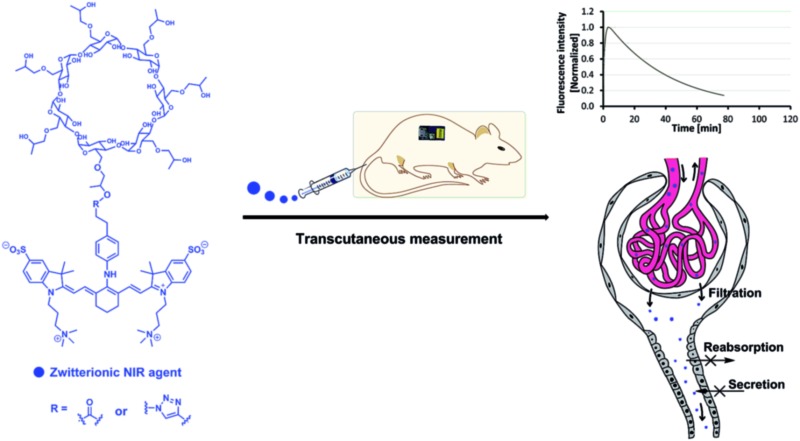
Zwitterionic near infrared fluorescent agents were developed for non-invasive real-time transcutaneous assessment of kidney function.

## Introduction

Evaluation of kidney function is crucial for a number of clinical situations.^1^ However, current approaches for kidney function assessment are time-consuming and cumbersome, and therefore delay definitive diagnosis.^2^ The glomerular filtration rate (GFR) is considered to be the best indicator for overall renal function. Plasma endogenous creatinine concentration is commonly used to estimate the GFR, but it may result in erroneous estimates due to age, gender, muscle mass and many other anthropometric variables.^3^ Determination of the plasma/urinary clearance of exogenous renal markers (such as ^99m^Tc-DTPA, inulin and iothalamate) is invasive and cumbersome, due to the requirement of multiple blood/urine sampling steps and tedious sample analysis by HPLC.^4^ Moreover, many studies revealed that creatinine and iothalamate are secreted by proximal tubule cells, while cystatin C and ^99m^Tc-DTPA are reabsorbed by the tubular epithelial cells, leading to a bias in the GFR.^1–4^ Many attempts have been made to tackle these problems. Recently, fluorescent GFR agents have gained much attention,^5–7^ however, very few fluorescent agents for GFR assessment have been reported. Although the fluorescence intensity of FITC-inulin in plasma can be measured over a specific time period after a bolus injection, the repeated blood sampling and the necessity to heat and dialyze the FITC-inulin solution makes the method cumbersome and still invasive in manner.^5^ Dorshow *et al.* reported that hydrophilic pyrazine-dicarboxylic acid derivatives can be excreted *via* the the kidneys, but the wavelength of pyrazine based fluorescent agents is still in the green region of the spectrum.^6^ Additionally, in our previous studies, we developed a non-invasive approach to assess kidney function in real-time based on a miniaturized electronic device attached to the skin and a GFR agent: FITC-sinistrin,^7^ which has better water solubility than FITC-inulin and there is no need to dialyze before injection. By these means, the elimination kinetics of the fluorescent agent FITC-sinistrin can be transcutaneously determined. The major advantage of this approach is the independence from blood/urine sampling and laboratory assays. This therefore allows renal function assessment in real-time and makes the evaluation of rapid changes in renal function possible, for example, in acute renal failure. Importantly, more precise results of the plasma clearance can be obtained *via* transcutaneous real-time measurement, which relies on a high number of data-points rather than a limited number of data-points from blood/urine sampling.^7^ Nevertheless, both inulin and sinistrin suffer from inherent limitations including high cost, limited availability, tedious extraction and purification from plant roots. Also, the wavelength of the aforementioned fluorescent GFR agents is still in the blue and green region of the spectrum, which further limits their application because of poor penetration and disturbance of the auto-fluorescence signal from tissue.^8^

**Scheme 1:**
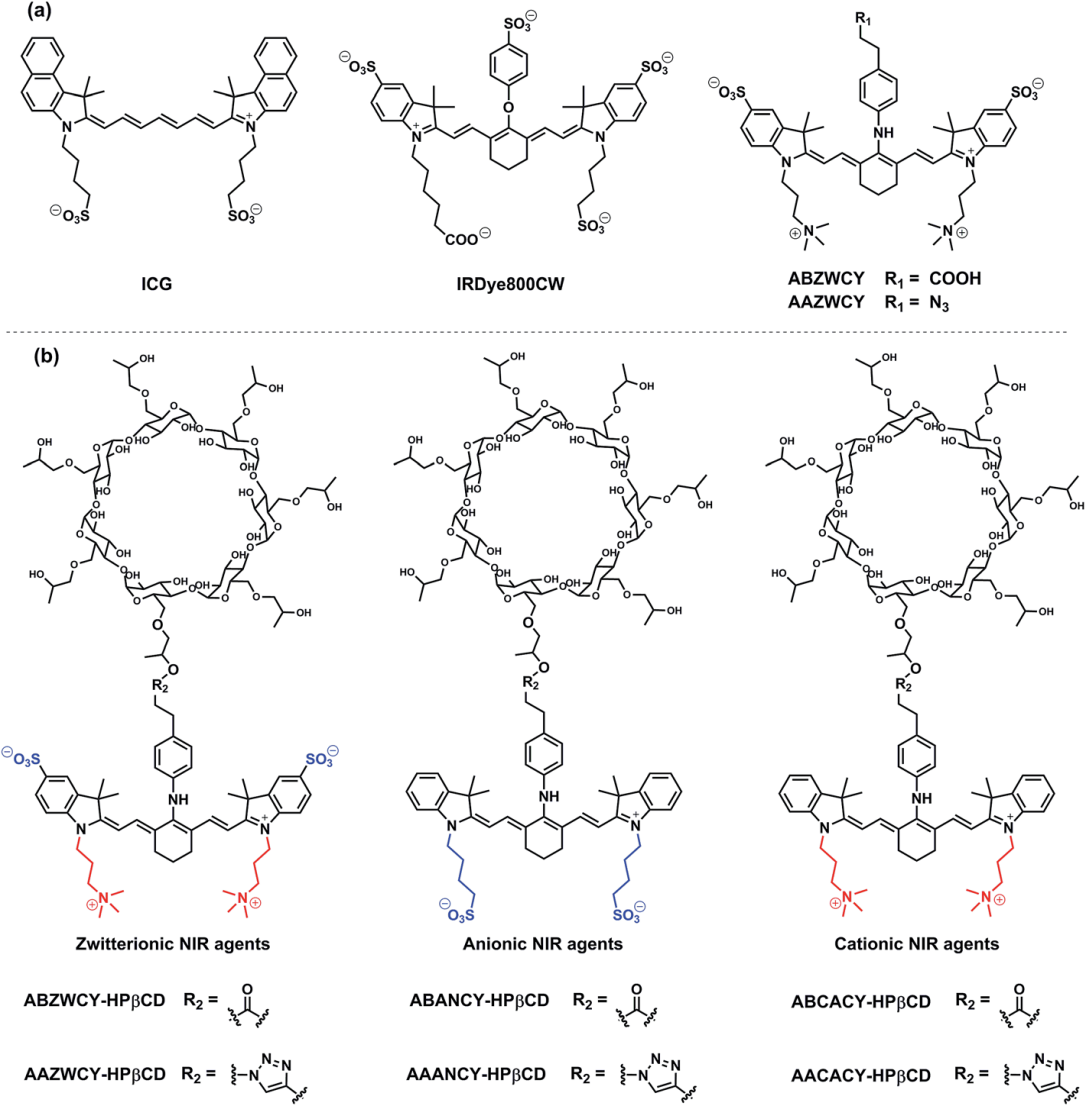
Structures of cyanine dyes and HPβCD based NIR agents.

One of the major obstacles encountered with non-invasive transcutaneous assessment of kidney function *in vivo* when using the aforementioned fluorescent GFR agents with a short emission wavelength (<600 nm) is the strong intrinsic auto-fluorescence background from living tissue, which significantly compromises the accuracy of measurement under physiological conditions.^8^ However, in the NIR region (650–900 nm), the absorption coefficient of tissue is greatly suppressed to a minimum level, thus it drastically reduces the noise from the auto-fluorescence and increases tissue penetration.^8^ Therefore, developing GFR agents in the NIR window is crucial for non-invasive transcutaneous assessment of kidney function. Zheng *et al.* focused on the development of renal clearable luminescent gold nanoparticles and used them to monitor the stages of kidney dysfunction, however, several terms are still unclear, such as plasma protein binding (PPB), urinary recovery, tubular reabsorption or secretion as well as potential toxicity.^9^ Several other studies pointed out that conventional organic fluorophores could persistently accumulate in the skin lipid membranes after intravenous injection, due to their high lipophilicity.^9^ What's worse is that the high lipophilicity leads to strong binding between fluorophores and plasma protein in serum, for example, indocyanine green (ICG, Scheme 1a) exhibits extremely high PPB (99%), liver uptake and excretion,^10^ and functions as a roadblock for kidney clearance. Many attempts have been made to overcome the above obstacles, for example, Hilger *et al.* have enhanced the hydrophilicity and decreased the PPB of dyes through increasing the number of sulfonate groups on asymmetric cyanine dyes.^11^ However, having regions of negative charge makes the dyes prone to being secreted or reabsorbed by the kidney proximal tubules.^11^ The fact that improving hydrophilicity and decreasing PPB comes at the expense of increasing tubular reabsorption or secretion prevents this method from gaining further application. Choi *et al.* reported a zwitterionic heptamethine cyanine dye,^12^ and although it has a decreased PPB and can be eliminated by the kidneys, its PPB (19%) is still very high compared to those ‘gold standard’ renal function agents such as inulin and iothalamate (9.5%),^13^ and this leads to a relatively long clearance half-life. Furthermore, its tubular reabsorption and excretion in the kidneys have not been studied. Therefore, to date, it is still an unmet challenge to develop an ideal GFR agent for non-invasive assessment of kidney function that simultaneously possesses the following features: (i) an absorption and emission wavelength in the NIR range for deeper penetration depth; (ii) high hydrophilicity and low PPB to accelerate renal clearance and avoid liver uptake; (iii) weakly charged characteristics to prevent interaction with organic transporter proteins in kidney tubules; (iv) no toxicity, no metabolism and capability to be completely excreted into urine; (v) filterable *via* the glomerulus, no tubular reabsorption and secretion in the kidneys.

Herein, we report the first NIR GFR agents with improved hydrophilicity and much lower PPB (<7%) through introducing zwitterionic charges and 2-hydroxylpropyl-β-cyclodextrin (HPβCD) into heptamethine cyanine dyes for assessment of kidney function in conscious rats. Relying on the attractive features of zwitterionic HPβCD based agents, which include high stability, non-toxicity and no metabolism *in vivo*, we demonstrate non-invasive, real-time monitoring of kidney function in an unprecedented efficient manner, with the zwitterionic HPβCD based agents being completely and rapidly excreted through the kidneys into the urine, exclusively filtered *via* the glomerulus, and with no reabsorption or secretion in the kidney proximal tubule. We further demonstrated that the zwitterionic HPβCD based agents can be used to assess kidney function in both healthy and nephropathic rats, which represents groundbreaking progress in the field of kidney function assessment and preclinical diagnosis.

## Results and discussion

### Design and synthesis

Based on the aforementioned criteria of an ideal GFR agent, a series of NIR agents were designed, which comprise two key functional components: HPβCD and NIR fluorophores. Firstly, introduction of HPβCD is used to increase hydrophilicity, decrease PPB and accelerate excretion, due to HPβCD having better water solubility than β-cyclodextrin (βCD) and higher stability against hydrolysis by α-amylases from either porcine or human origin,^14^ and it allows the encapsulation of the lipophilic fluorophore into the cavity of HPβCD. The merit of its non-toxicity resulted in FDA approval ten years ago.^14^ Additionally, its well-defined structure, low cost and excellent availability make it an ideal component for GFR agents in comparison with inulin and sinistrin. Secondly, NIR fluorophores were employed for labeling HPβCD, thus, providing a deeper penetration depth and minimizing the disturbance of auto-fluorescence from skin tissue during transcutaneous measurements. NIR fluorescent agents with three different molecular surface charge characteristics (zwitterionic, anionic and cationic in Scheme 1b) were developed to systematically study the influence of different molecular surface charges on their PPB and excretion. The detailed synthetic procedures are described in the ESI.† Three parent cyanine dyes (ZWCY, ANCY and CACY) have been modified with sulfonate groups and quaternary ammonium cations to impart negative charges and positive charges, respectively. Starting from phenylhydrazine or 4-hydrazino-benzenesulfonic acid,^12^ carboxylic acid or azide-tagged NIR dyes were synthesized (Fig. S1†) by reacting the *meso* chlorine atom of three parent cyanine cores with the appropriate nucleophiles. Finally, these tagged dyes were conjugated with HPβCD or propynyl-HPβCD to obtain the corresponding agents (Fig. S2 and S4†). The intermediates and products were characterized by ^1^H-NMR, ^13^C-NMR and LR-MS.

### Physicochemical characteristics and optical properties

**Fig. 1:**
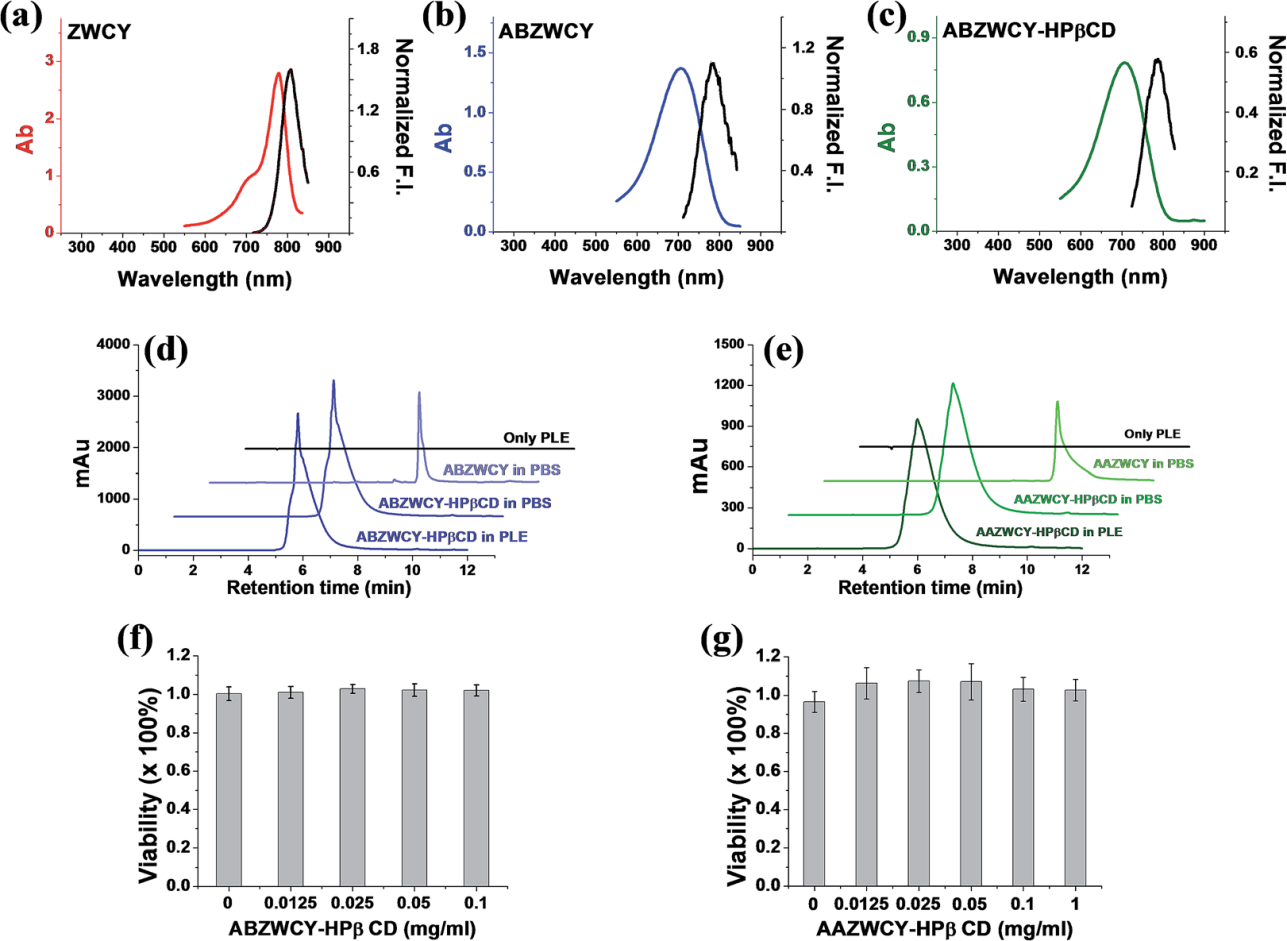
Absorption and emission spectra of (a) ZWCY (0.05 mg mL^–1^), (b) ABZWCY (0.02 mg mL^–1^), and (c) ABZWCY-HPβCD (2 mg mL^–1^) in PBS. (d and e) High performance liquid chromatography (HPLC, monitored at 710 nm) spectra of ABZWCY-HPβCD and AAZWCY-HPβCD incubated with porcine liver esterase (PLE) for 24 h. (f and g) Cell viability of HK-2 human proximal tubular cells incubated with ABZWCY-HPβCD and AAZWCY-HPβCD in MTT assays.

The absorption and fluorescence spectra are depicted in Table 1, and Fig. 1, S5 and S6.† The parent dyes such as ZWCY, ANCY and CACY display an absorption peak at around 780 nm and an emission peak at roughly 810 nm with a small Stokes-shift of 30 nm. As expected, modification with an aromatic amine moiety at the chloro atom position significantly increased the Stokes-shift up to 80 nm. The introduction of HPβCD moieties on those dyes does not cause any shifts in the spectrum. Notably, their spectra effectively match with the configuration of the transcutaneous device, which consists of two light-emitting diodes with an excitation wavelength of 700 nm and a photodiode for emission wavelength detection at 790 nm (Fig. S10†). Moreover, the absence of reagent and free dye peaks in the optical spectrum and HPLC curves (Fig. 1, S5 and S9†), as well as in the NMR spectra, proves the high purity of ABZWCY-HPβCD and AAZWCY-HPβCD.

**Table 1:** Photo-physical properties of the three parent dyes and the corresponding HPβCD based agents

Surface charge	Compounds	*λ*_ab_ (nm) PBS/plasma	*λ*_em_ (nm) PBS/plasma	Stokes shift (nm) PBS/plasma	*ε* ^*b*^ (M^–1^ cm^–1^) PBS/plasma	*φ* ^*c*^ (%) in plasma	Net charge	log *D* (pH = 7.4)	PPB
Zwitterionic	ZWCY	778/780	808/810	30/30	234 000/218 000	10.2	+1	–0.97	ND
	ABZWCY	706/700	790/792	84/92	76 000/74 000	3.2	0	–3.28	19%
	ABZWCY-HPβCD	706/704	790/790	84/86	ND^*a*^	ND	+1	–9.50	3.7%
	AAZWCY	708/710	791/792	83/82	77 000/74 800	3.8	+1	–0.22	15.7%
	AAZWCY-HPβCD	708/712	791/796	83/84	ND	ND	+1	–9.72	6.5%
Anionic	ICG	782/785	810/806	28/21	121 000/118 000	9.1	–1	7.88	99%
	IRDye800CW	774/774	798/800	24/26	240 000/237 000	14.2	–4	2.51	41%
	ANCY	782/782	808/804	26/22	193 000/189 000	8.4	–1	3.63	ND
	ABANCY	726/720	790/786	64/66	72 200/71 800	4.1	–2	1.40	95%
	ABANCY-HPβCD	726/722	790/788	64/66	ND	ND	–1	–4.90	19.7%
	AAANCY	730/728	794/792	64/64	78 500/76 000	4.5	–1	4.39	80%
	AAANCY-HPβCD	730/730	794/792	64/62	ND	ND	–1	–5.11	26.8%
Cationic	CACY	772/770	808/810	36/40	215 000/222 000	7.9	+3	–3.38	ND
	ABCACY	716/710	796/798	80/88	60 060/61 100	4.4	+2	–2.00	54.5%
	ABCACY-HPβCD	716/722	796/804	80/82	ND	ND	+3	–11.91	23.5%
	AACACY	720/716	798/798	78/82	80 840/82 000	3.5	+3	–2.62	60%
	AACACY-HPβCD	718/716	794/790	76/74	ND	ND	+3	–12.12	27.4%

^*a*^Not determined.

^*b*^ *ε*: molar extinction coefficient.

^*c*^ *φ*: fluorescence quantum yield; *λ*_ab_ and *λ*_em_: wavelength of maximum absorbance and emission, respectively.

The log *D* value (distribution coefficient) at pH 7.4 for each compound was calculated using the JChem plugin of ChemAxon (Table 1). It indicated that all of the compounds have high hydrophilicity, especially for the HPβCD based agents. The net charge of the compounds spans from –2 to +3, depending on the number of attached sulfonate and/or tetraethyl ammonium groups. The degree of labeling (DOL) is defined as the average number of dye molecules coupled to HPβCD. To preserve the properties of HPβCD, the DOL of ABZWCY-HPβCD and AAZWCY-HPβCD was controlled below 0.05 with small variations (±0.004) between different batches of products (Table S3†), suggesting good reproducibility of such labeling procedures. Molar extinction coefficients and fluorescence quantum yields for each compound are shown in Table 1, except for the HPβCD based agents, as their extinction coefficients and quantum yields are dependent on their DOL.

### Plasma protein binding (PPB)

The interaction between fluorescent dyes and plasma proteins is particularly important as it affects the capability of the dyes as GRF agents in the following aspects: bio-distribution and clearance pathways as well as the rate of excretion *in vivo*.^15^ For example, in our previous study, we found that the commercial anionic dye IRDye800CW has a high PPB up to 41%.^16^ It is also reported that IRDye800CW exhibits skin accumulation after intravenous injection and cannot be excreted rapidly by the kidneys.^9*b*^ We first evaluated the PPB values for free dyes, anionic dyes and cationic dyes, which exhibit very high PPB values, as shown in Table 1, for example, ABANCY and AAANCY show 95% and 80% PPB, respectively. ABCACY and AACACY display 54.5% and 60% PPB, respectively. Meanwhile, the zwitterionic dyes ABZWCY and AAZWCY have much lower PPB (19% and 15.7%, respectively) than all other free dyes, which indicated that zwitterionic dyes possess much lower binding abilities towards plasma proteins than anionic and cationic dyes. Similar PPB values were also observed in the mixture of free dye (ABZWCY and AAZWCY) and HPβCD, with values of 17.7% and 13.7%, respectively. Attaching HPβCD to free dyes could further decrease the PPB, for example, anionic HPβCD based agents (ABANCY-HPβCD and AAANCY-HPβCD) and cationic HPβCD based agents (ABCACY-HPβCD and AACACY-HPβCD) exhibit PPB values ranging from 20% to 30%. Surprisingly, the zwitterionic agents ABZWCY-HPβCD and AAZWCY-HPβCD have much lower PPB (3.7% and 6.5%, respectively), even lower than some ‘gold standard’ agents. Dynamic light scattering experiments further demonstrated that no aggregation occurred on either zwitterionic agents or their mixture with rat plasma protein (Fig. S8†). The above results demonstrate that HPβCD modification could significantly decrease the PPB for all three types of charged free dyes. Additionally, PPB is not only dependent on the dye hydrophilicity, but is also related to the molecular charge number and distribution. Since albumin is alkalotic, acidic agents will preferentially bind to albumin, then to lipoprotein after albumin becomes saturated. Basic agents will bind to the acidic alpha-1 acid glycoprotein.^17^ Thus, anionic and cationic compounds are more inclined to bind to proteins than zwitterionic compounds. Therefore, we will mainly focus on zwitterionic compounds in the following parts.

### Stability studies in esterase and cell viability evaluated by MTT assay

In order to evaluate the stability of conjugation bonds between HPβCD and NIR fluorophores, we co-incubated ABZWCY-HPβCD and AAZWCY-HPβCD with porcine liver esterase (PLE) for 24 h, respectively, and then tracked their stability with HPLC (Fig. 1d and e). A peak for ABZWCY-HPβCD and AAZWCY-HPβCD in both PBS and PLE appeared at 5.8 min and 6.0 min, respectively, while a unimodal peak for free dye ABZWCY and AAZWCY in PBS appeared at 8.0 min and 9.0 min, respectively. No degradation was observed for both NIR agents that were co-incubated with PLE, as indicated by there being no appearance of a free dye peak in the HPLC chromatographs. This supports the observation that both the triazole unit of AAZWCY-HPβCD from a classic click reaction and the ester bond of ABZWCY-HPβCD are highly stable in esterase conditions.

The cytotoxicity of ABZWCY-HPβCD and AAZWCY-HPβCD was evaluated using a 3-(4,5-dimethyl-2-thiazoly)-2,5-diphenyltetrazolium bromide (MTT) assay, with the results (Fig. 1f and g) showing that HK-2 human proximal tubular cells maintained high viability after being incubated with varying concentrations of these two agents, suggesting no cytotoxic effect from ABZWCY-HPβCD and AAZWCY-HPβCD.

### Transcutaneous measurement of kidney function in a healthy rat model

**Fig. 2:**
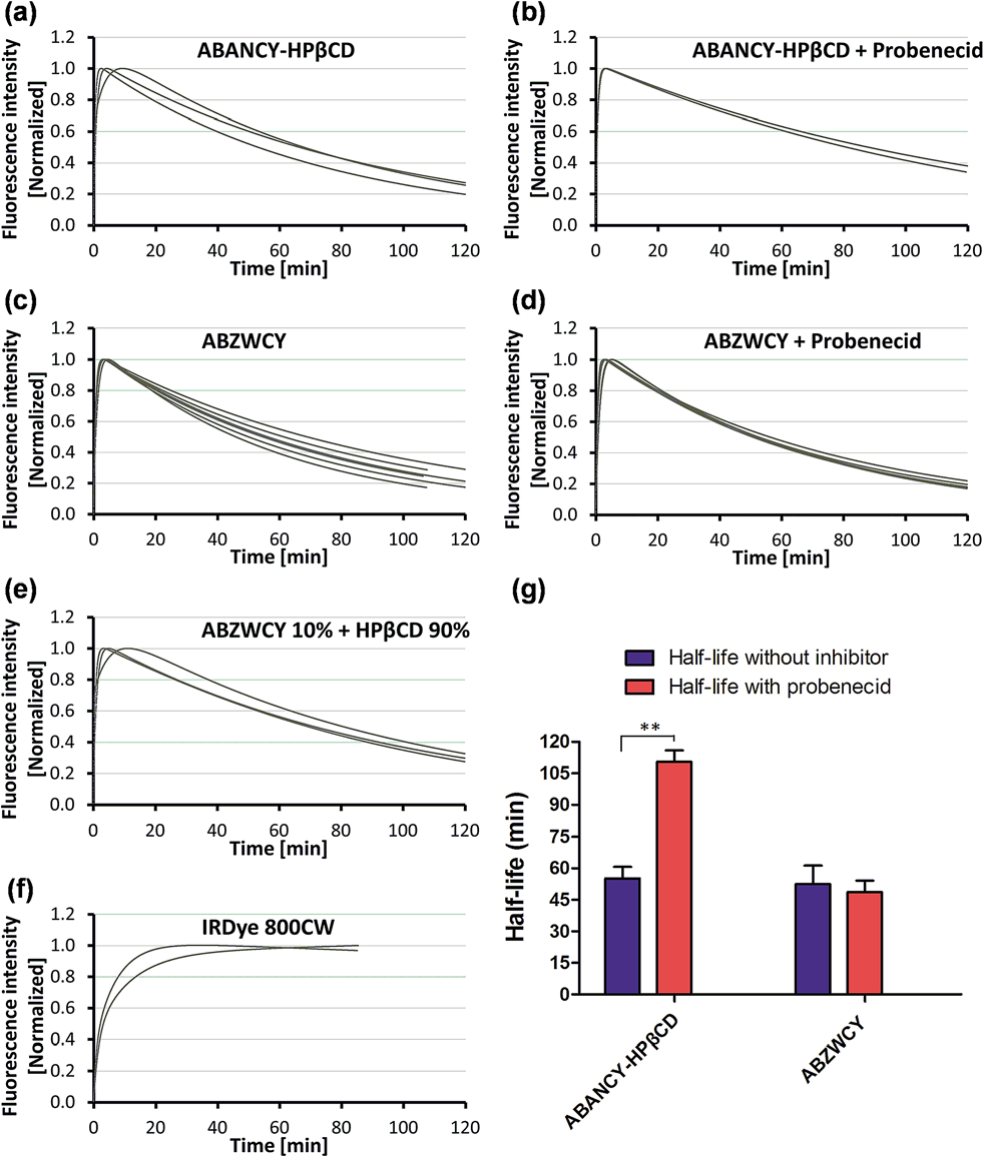
Elimination curves of ABANCY-HPβCD (a and b, *n* = 3), ABZWCY (c and d, *n* = 6), ABZWCY mixed with HPβCD (e, *n* = 3) and IRDye800CW (f, *n* = 2) by transcutaneous measurements in healthy rats in the absence and presence of probenecid, *n* means the number of rats. (g) Clearance half-life for ABANCY-HPβCD and ABZWCY in the absence and presence of probenecid. **P < 0.01.

**Fig. 3:**
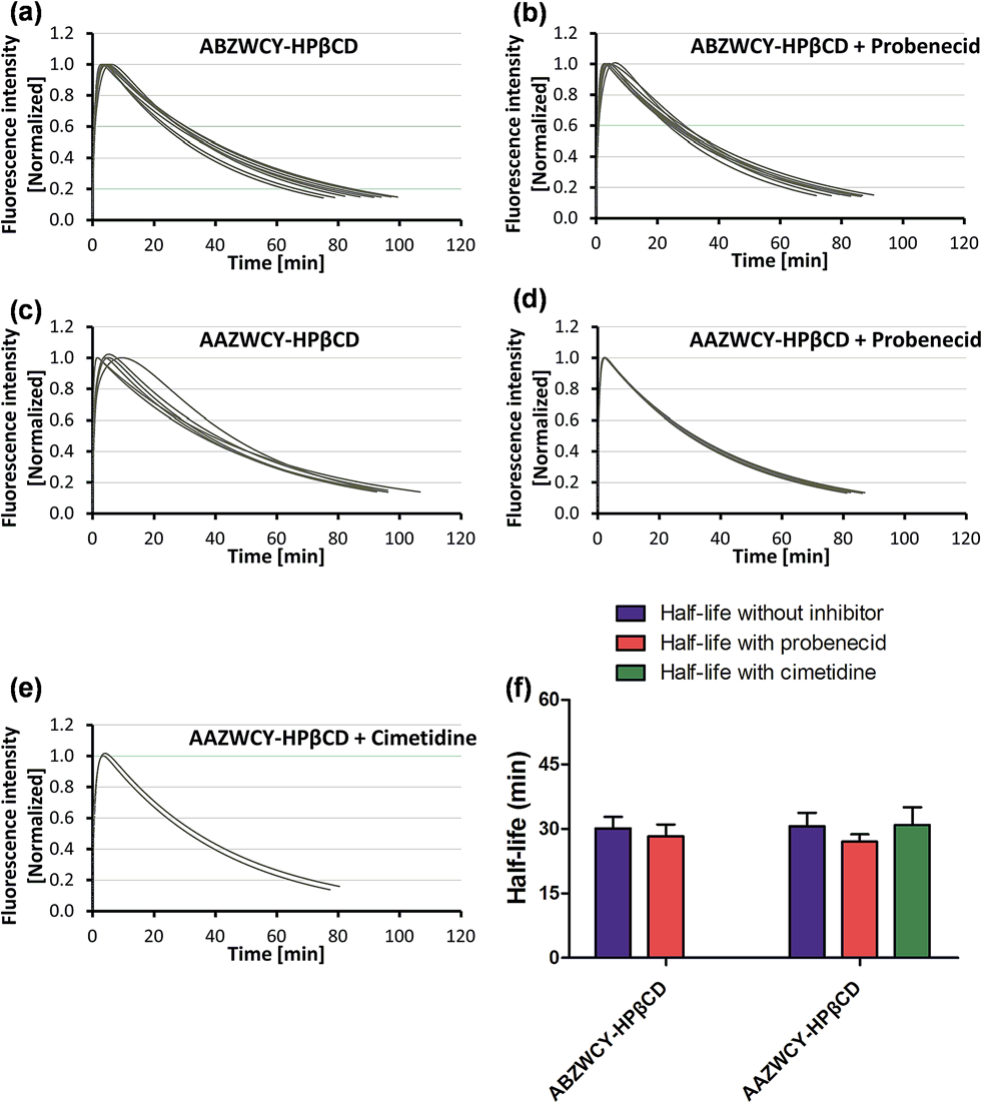
Elimination curves of ABZWCY-HPβCD (a and b, *n* = 8) and AAZWCY-HPβCD (c and d, *n* = 6; e, *n* = 2) by transcutaneous measurements in healthy rats in the absence and presence of probenecid or cimetidine, *n* means the number of rats. (f) Clearance half-life for ABZWCY-HPβCD and AAZWCY-HPβCD in the absence and presence of probenecid or cimetidine.

Having demonstrated low PPB and no cytotoxicity of the zwitterionic ABZWCY-HPβCD and AAZWCY-HPβCD NIR agents, we proceeded to study whether these zwitterionic compounds can be excreted by the kidneys in healthy rats (8–10 weeks), by conducting non-invasive transcutaneous measurements. The principles and methods of the transcutaneous technique are described in Fig. S10.† Firstly, as a control, IRDye800CW was chosen to be injected intravenously into rats and its elimination curves were measured transcutaneously. However, its clearance curves show no decay even at 90 min post-injection (Fig. 2f), consistent with previous findings.^9*b*^ A long retention time of IRDye800CW *in vivo* was attributed to its higher PPB (41%) and negative charge feature, leading to skin accumulation and slow renal excretion. In contrast, the anionic HPβCD based agent ABANCY-HPβCD exhibited an obvious decay in the clearance curves (Fig. 2a) and had a half-life of 55.2 ± 5.86 min (Table 2), which is attributed to its lower PPB (19.7%) than IRDye800CW. To assess whether the anionic agent ABANCY-HPβCD is reabsorbed or secreted by organic anion transporter (OAT) proteins in the kidney tubules, probenecid, an inhibitor of OAT proteins,^18^ was administered (30 min prior to injection of ABANCY-HPβCD) to block tubular reabsorption and the secretion pathway, then, elimination curves were measured for the same rats. However, ABANCY-HPβCD has severe tubular secretion, as evidenced by slower clearance curves and a longer clearance half-life (110.5 ± 5.4 min, Table 2 and Fig. 2b and g). We also measured the cationic HPβCD based agent in the same manner and the results are similar to those of anionic HPβCD based agents. Next, the free dye ABZWCY was injected intravenously and its elimination curves were also recorded transcutaneously. As shown in Fig. 2c, its fluorescence kinetics curves declined, but did not return to background levels at 2 h post-injection, suggesting it was not excreted completely during this period. The half-life of the free dye ABZWCY (52.6 ± 8.8 min) is similar to that of ABANCY-HPβCD. This is in agreement with their similar PPB values (∼20%). To determine whether ABZWCY has tubular reabsorption or secretion in addition to filtration, probenecid was administered to block tubular reabsorption and the secretion pathway. It was found that the half-life of ABZWCY (48.7 ± 5.4 min, Table 2 and Fig. 2g) only slightly changes in the presence of probenecid, demonstrating that tubular reabsorption or secretion is a minor factor in half-life alterations. This result also indicated that both anionic and cationic compounds are more likely to be secreted by the kidney tubules than zwitterionic compounds are. In addition, a mixture of ABZWCY and HPβCD was studied, with the clearance curves (Fig. 2e) and half-life (54.2 ± 7.4 min) being similar to those of ABZWCY only, indicating that free HPβCD has no effect on the excretion of ABZWCY. Surprisingly, compared to anionic agents and free zwitterionic dye, a much faster clearance decay and a much shorter half-life were observed for ABZWCY-HPβCD and AAZWCY-HPβCD either with or without probenecid treatment (Table 2 and Fig. 3).

**Table 2:** Urinary recovery and clearance half-life with and without probenecid treatment

Entry	Urinary recovery (%) in 24 h	*T*_1/2_ ^*c*^ without probenecid (min)	*T*_1/2_ with probenecid (min)
ABZWCY	99.4 ± 4.8 (3)^*b*^	52.6 ± 8.8 (6)	48.7 ± 5.4 (6)
ABZWCY mixed with HPβCD (1 : 9)^*a*^	96.5 ± 5.8 (3)	54.2 ± 7.4 (3)	ND^*d*^
ABZWCY-HPβCD	97 ± 3.9 (3)	30.1 ± 2.7 (8)	28.3 ± 2.7 (8)
AAZWCY-HPβCD	103.3 ± 4.2 (3)	30.6 ± 3.1 (6)	27 ± 1.7 (6)
ABANCY-HPβCD	ND^*d*^	55.2 ± 5.6 (3)	110.5 ± 5.4 (3)

^*a*^The weight ratio of ABZWCY to HPβCD: 1/9.

^*b*^Number of rats.

^*c*^ *T*_1/2_: clearance half-life.

^*d*^Not determined.

Those results indicated that zwitterionic HPβCD based agents have no skin accumulation. Their shorter clearance half-life (∼30 min) was attributed to their evenly distributed charges, higher hydrophilicity and lower PPB (<7%). Importantly, a negligible difference in the clearance half-life (Fig. 3f) with and without probenecid treatment was observed with both zwitterionic HPβCD based agents, indicating that they have no severe tubular reabsorption or secretion in the kidneys. We also found that the half-life of the mixture of ABZWCY and HPβCD is much longer than that of ABZWCY-HPβCD and AAZWCY-HPβCD, therefore, ABZWCY and its HPβCD mixture are not superior to ABZWCY-HPβCD and AAZWCY-HPβCD as suitable candidates for kidney function assessment. Considering that ABZWCY-HPβCD and AAZWCY-HPβCD possess a positively charged quaternary ammonium group, it is necessary to determine whether these zwitterionic HPβCD based agents can be reabsorbed or secreted by organic cation transporter (OCT) proteins in kidney tubules.^18^ AAZWCY-HPβCD was injected into healthy rats and elimination curves and clearance half-life values were measured in the presence of an OCT inhibitor, cimetidine. The results show that the OCT inhibitor has no effect on its clearance half-life (30.9 ± 4.1 min, Fig. 3e and f). Based on all of the above results, we conclude that zwitterionic HPβCD based agents can be filtrated *via* the glomerulus efficiently, and exhibit slight differences in the clearance half-life by both tubule OAT and OCT proteins in kidneys.

### Urinary recovery

**Fig. 4:**
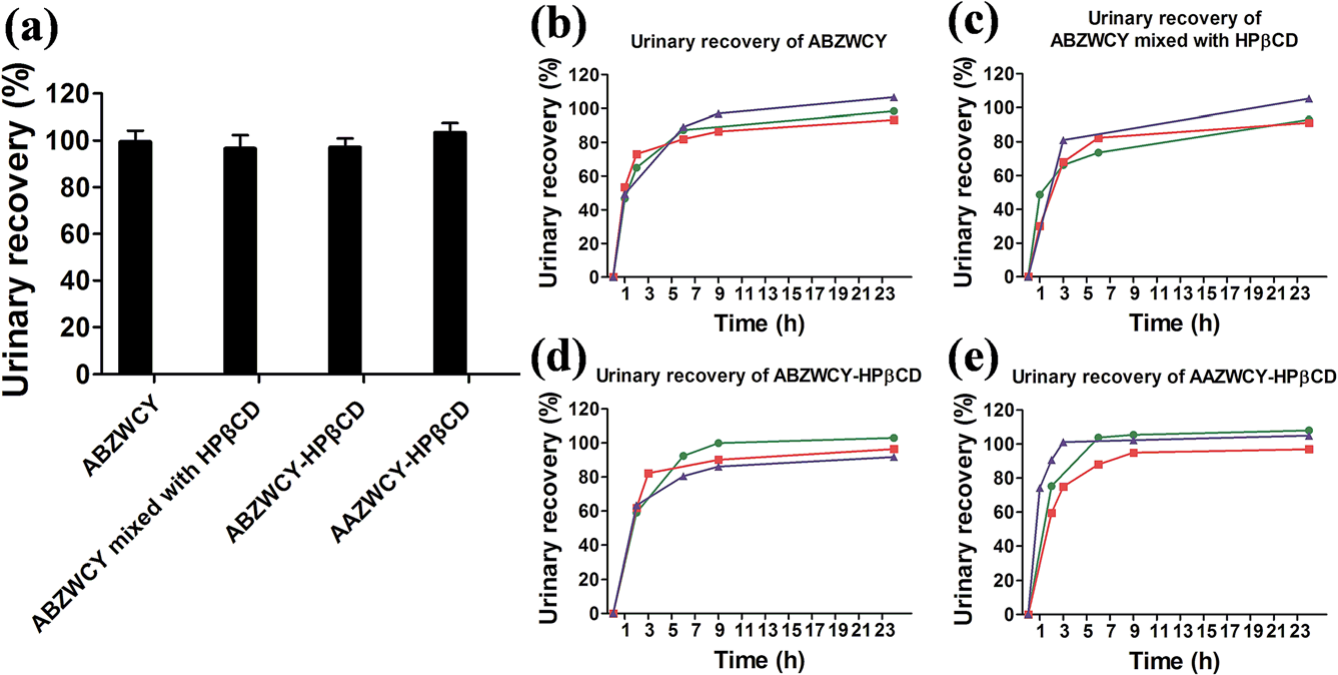
(a) Urinary recovery for each marker in healthy rats within 24 h. Urinary recovery-time curves of (b) ABZWCY (*n* = 3), (c) ABZWCY mixed with HPβCD (*n* = 3), (d) ABZWCY-HPβCD (*n* = 3) and (e) AAZWCY-HPβCD (*n* = 3), *n* means the number of rats.

An ideal kidney function agent should have no metabolism *in vivo* and be recovered completely in urine. With this in mind, we investigated the recoveries of injected doses using *in vivo* experiments with metabolic cages. High urinary recoveries were determined for the free dye ABZWCY and its mixture with HPβCD (99.4 ± 4.8% and 96.5 ± 5.8%, respectively, Fig. 4 and Tables 2 and S6†). Similar tendencies of urinary recoveries of ABZWCY-HPβCD and AAZWCY-HPβCD with values of 97 ± 3.9% and 103.3 ± 4.2%, respectively, were also observed (Fig. 4, and Tables 2 and S7†). Indeed, urinary recoveries of the given dose were almost completed at 9 h post-injection for ABZWCY-HPβCD and AAZWCY-HPβCD, which is faster than the free dye ABZWCY and in agreement with their shorter clearance half-life. The results demonstrated that all of the injected zwitterionic agents are excreted rapidly into urine. However, ABANCY-HPβCD has a relatively high PPB and long clearance half-life and exhibits severe kidney tubular secretion, so it is not considered for further urinary recovery studies.

### Biodistribution

**Fig. 5:**
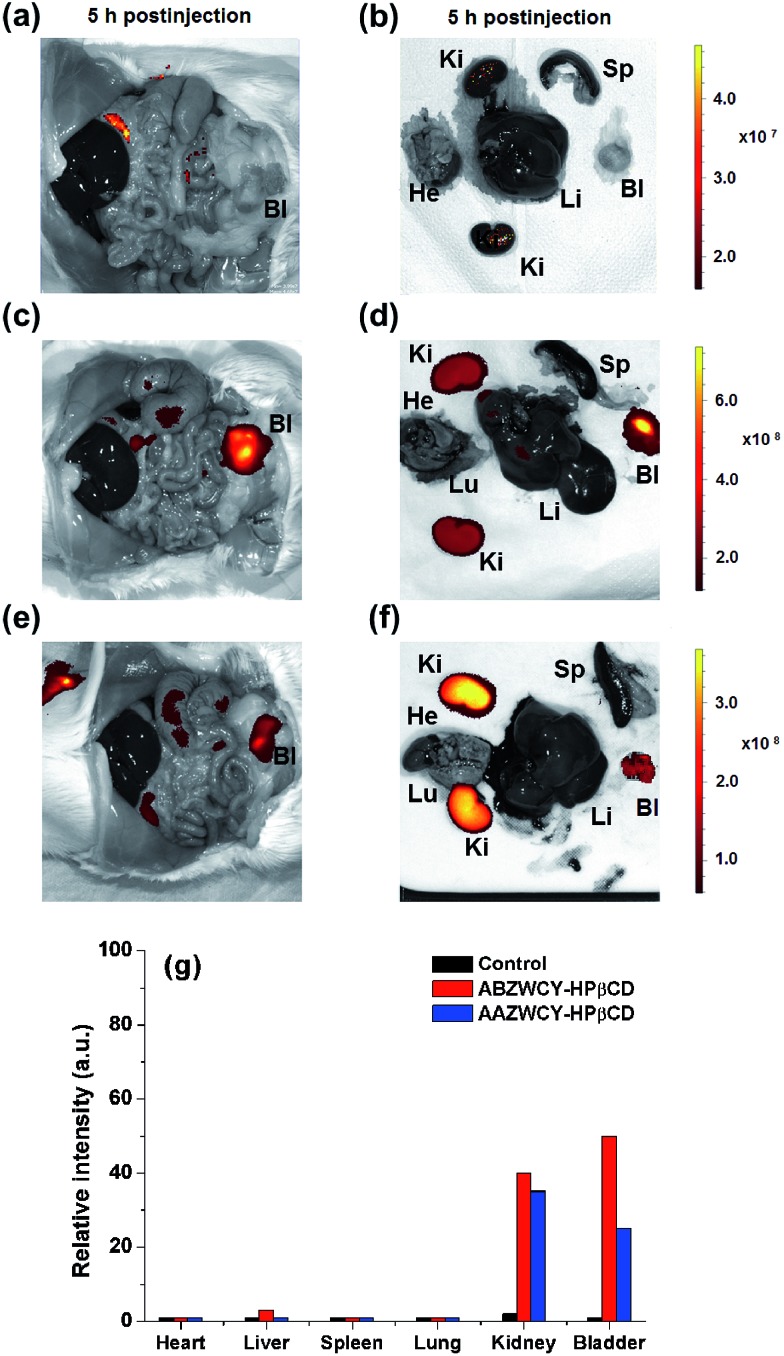
*In vivo* biodistribution and clearance of zwitterionic HPβCD based agents in healthy rats. Saline (a and b), ABZWCY-HPβCD (c and d) and AAZWCY-HPβCD (e and f) were injected intravenously into rats 5 h prior to imaging. Abbreviations: Bl, bladder; He, heart; Ki, kidney; Li, liver; Lu, lung; Sp, spleen. (g) Relative fluorescence intensity of organs in panels b, d and f.

To confirm the distribution of the zwitterionic HPβCD based agents after intravenous injection in healthy rats, we investigated the fluorescence distribution of ABZWCY-HPβCD and AAZWCY-HPβCD using small animal imaging. As expected, the fluorescence images of the organs obtained from the control rat displayed almost no fluorescence signal (Fig. 5a and b). Notably, fluorescence signals can be mainly observed in the kidneys and bladder from the rats that were administered with these two zwitterionic HPβCD based agents, with only slight signals in the intestines and no significant nonspecific uptake in the other organs and tissues (Fig. 5c–g). These experiments further confirm that these two zwitterionic HPβCD based agents were excreted through the kidneys to the urine.

### Metabolism studies

To determine whether these agents can be metabolized *in vivo*, urine samples were collected and investigated by HPLC. The results (Fig. S12†) indicated that no metabolites were found from the HPLC curves. To better understand these results, we performed an additional experiment using MALDI-TOF. The obtained MALDI data (Fig. S12†) show that the mass distribution of ABZWCY-HPβCD and AAZWCY-HPβCD recovered from urine samples is the same as that before injection. These results further confirmed that these two zwitterionic HPβCD based agents have no metabolism *in vivo*.

### Transcutaneous measurement of kidney function in a nephropathic rat model

**Fig. 6:**
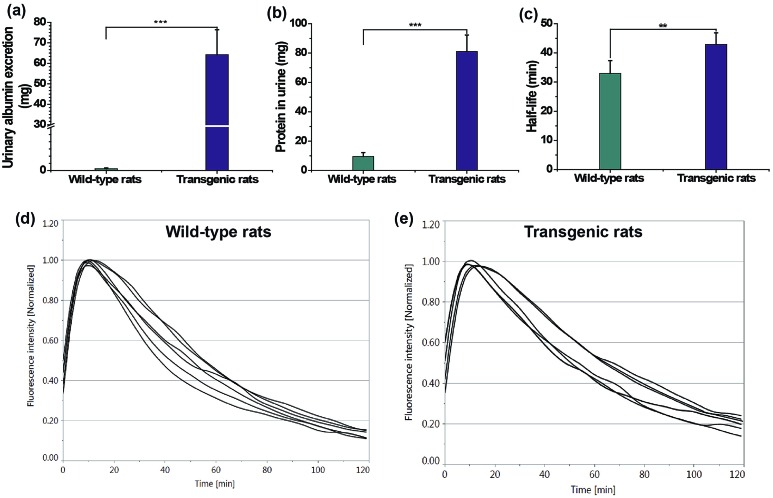
Urinary albumin excretion (a, ***P < 0.001), protein excretion (b, ***P < 0.001) and clearance half-life of ABZWCY-HPβCD (c, **P < 0.01) in wild-type rats and AT1R transgenic rats. Elimination curves of ABZWCY-HPβCD by transcutaneous measurements in wild-type rats (d) and AT1R transgenic rats (e).

Finally, transcutaneous assessments of kidney function were conducted in a transgenic rat (TGR) model (24 weeks) and age-matched wild-type rats. A TGR model with overexpression of the human Ang II type 1 receptor (hAT1R) in podocytes was used.^19^ In this TGR based nephropathic model, the damage progressed to nephron loss *via* focal segmental glomerulosclerosis, leading to the degeneration of both the glomerulus and tubules, which is associated with a reduction in the glomerular filtration rate, tubular necrosis and protein leakage in urine.^19^ The loss of kidney function in the nephropathic model was testified by the urinary parameters including protein and albumin excretion in Table 3 and Fig. 6a and b, consistent with previous findings.^19*a*^ The clearance half-life of ABZWCY-HPβCD in AT1R transgenic rats (42.88 ± 3.97 min, Table 3 and Fig. 6e) is much longer than that observed in the wild-type rats (32.98 ± 4.35 min, Table 3 and Fig. 6d), which is consistent with the fact that urinary protein and albumin excretion of AT1R transgenic rats are much higher than those in the wild-type rats. Additionally, we found that the clearance half-life of wild-type rats (32.98 ± 4.35 min) is slightly longer than that of the afore-measured rats (30.1 ± 2.7 min), which is attributed to older rats and an age-related decline in kidney function. These results show the potential of NIR zwitterionic agents as exogenous markers for evaluating kidney function in a kidney disease model.

**Table 3:** Urinary protein, albumin excretion and clearance half-life of ABZWCY-HPβCD in wild-type and AT1R transgenic rats

Entry	Urinary protein excretion (mg)	Urinary albumin excretion (mg)	Half-life (min)
Wild-type rats	9.5 ± 2.7 (4)^*a*^	0.4 ± 0.18 (4)	32.98 ± 4.35 (6)
Transgenic rats	81.1 ± 11.2 (4)	64.2 ± 12.2 (4)	42.88 ± 3.97 (6)

^*a*^Number of rats.

## Conclusions

In summary, zwitterionic NIR GFR agents ABZWCY-HPβCD and AAZWCY-HPβCD were rationally designed and synthesized. They show favourable fluorescence properties, deeper penetration depths, high hydrophilicity and extremely low PPB. Furthermore, they also exhibit high stability in esterase and non-toxicity. By taking advantage of the above properties, we have demonstrated that these zwitterionic NIR agents outperform the existing commercial NIR dye (IRDye800CW) in the context of skin accumulation and PPB. More importantly, these two zwitterionic HPβCD based agents can be excreted efficiently through the kidneys into urine without severe reabsorption and secretion in the kidney tubules. Urinary recovery and fluorescence distribution investigations by small animal imaging experiments further demonstrated that they could be completely and rapidly excreted through the kidneys without *in vivo* metabolism. Studies in rat models of both healthy models and those with kidney disease showed that zwitterionic HPβCD based agents are promising markers for evaluating kidney function. To the best of our knowledge, this is the first report on assessing kidney function based on near infrared GFR agents and the first example of using zwitterionic charge characteristics for developing GFR agents. Relying on these zwitterionic NIR agents and transcutaneous fluorescence detection techniques, we demonstrate a rapid, robust and convenient approach for non-invasive real-time assessment of kidney function, without the need for time-consuming blood/urine sample preparation. We believe that this work represents a significant progress towards highly efficient kidney function assessment, and holds great promise in kidney disease diagnosis in the future. Formal preclinical development studies are in progress.
