# Supplementary material for: Zwitterionic near infrared fluorescent agents for noninvasive real-time transcutaneous assessment of kidney function
Source: Chem Sci. 2017 Jan 11;8(4):2652–60. doi: 10.1039/c6sc05059j (PMC5431684; doi:10.1039/c6sc05059j)
Supplement: Supplementary file 1 [file SC-008-C6SC05059J-s001.pdf]

Electronic Supplementary Material (ESI) for Chemical Science.
This journal is © The Royal Society of Chemistry 2017

Supporting Information

Zwitterionic Near Infrared Fluorescent Agents for Noninvasive Real-time Transcutaneous Assessment of Kidney Function

*Jiaguo Huang, † Stefanie Weinfurter, † Cristina Daniele, † Rossana Perciaccante,‡ Rodeghiero Federica,‡ Leopoldo Della Ciana, ‡ Johannes Pill,† Norbert Gretz †, **

†Medical Research Center, Medical Faculty Mannheim, University of Heidelberg, Theodor-Kutzer-Ufer 1-3, 68167, Mannheim, Germany

‡Cyanagen S.r.l., Via degli Stradelli Guelfi 40/C 40138 Bologna BO, Italy

**Corresponding author*

E-mail: Norbert.Gretz@medma.uni-heidelberg.de

List of Contents

1. General procedures
2. Synthesis and chemical characterization
3. Optical properties characterization
4. Degree of labeling measurements
5. Plasma protein binding and dynamic light scanner analysis
6. Stability studies in porcine liver esterase and purity investigation in HPLC
7. Cell viability evaluated by MTT assay
8. Principle and methods for transcutaneous measurements of kidney function in a healthy rat model
9. Biodistribution studies by small animal imaging
10. Urinary recovery of injected dose in urine studies
11. Determination of urinary metabolites
12. Transcutaneous measurements of kidney function in a nephropathy rat model
13. NMR and mass spectrum

1. General Procedures

All reagents and deuterated solvents were purchased from Sigma Aldrich or Carl Roth and used as received. Silica gel (Silicycle, 230-400 mesh) was used for column chromatography. NMR spectra were recorded on a Bruker 300 MHz NMR instrument. Chemical shifts are reported in ppm relative to residual protic solvent resonances. Mestre Nova LITE v5.2.5-4119 software (Mestre lab Research S.L.) was used to analyze the NMR spectra. Matrix-assisted laser desorption/ionization time-of-flight (MALDI-TOF) analyses were collected on a Bruker ultraflex TOF/TOF instrument. UV-vis and fluorescence spectra were acquired using a microplate reader (Tecan Infinite M200) and an Eppendorf biospectrometer kinetic device using quartz cuvettes (1 cm path length). HPLC analysis and separations were carried out on a Thermo scientific ultimate 3000 liquid chromatography using Ascentis® C18 columns. The pH of samples solution was tested by Mettler Toledo FiveEasy™ FE20pH bench meter. Fluorescent bio-distribution was conducted by using small animal imaging (PerkinElmer). IUPAC names of all compounds are provided and were determined using CS ChemBioDraw Ultra 12.0. Dynamic light scattering studies were conducted using a Malvern Zetasizer Nano S90 equipment. The near infrared transcutaneous devices are available from Mannheim Pharma & Diagnostics, Mannheim, Germany.

2. Synthesis and chemical characterization

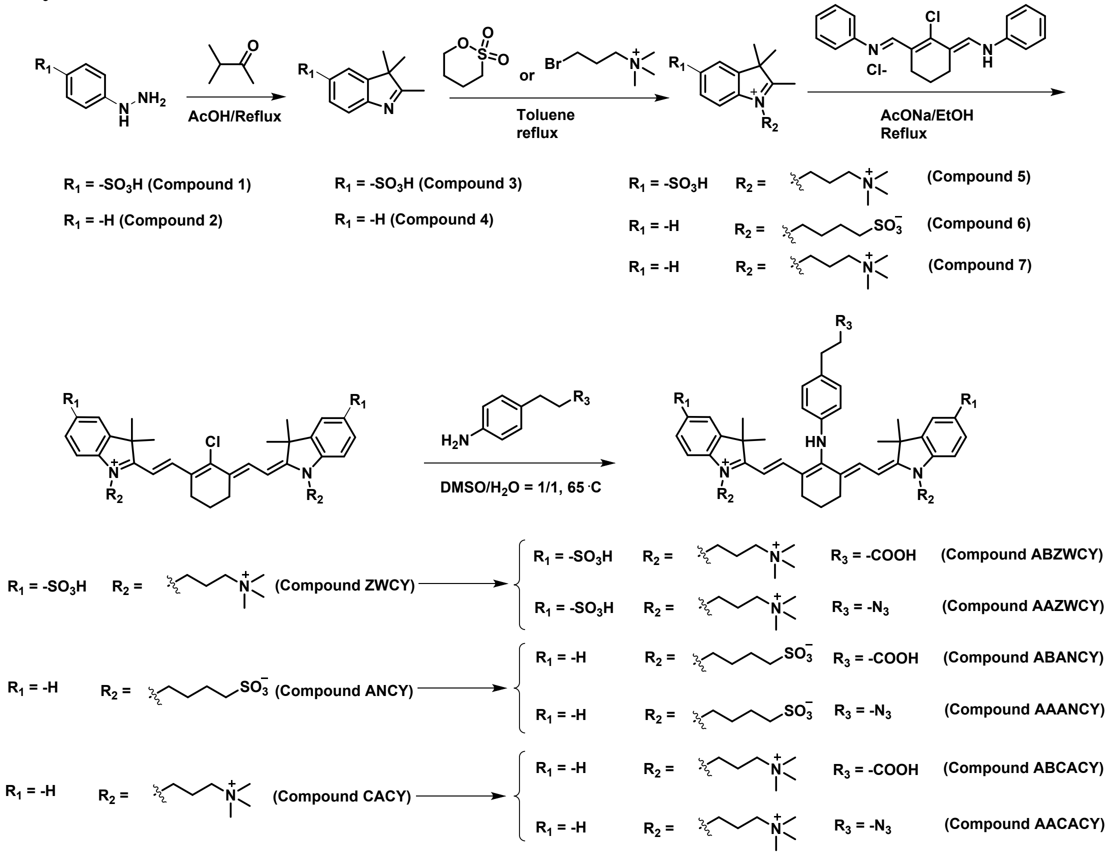

Figure S1. Synthesis of three different charged cyanine dyes.

Synthesis of compound 3 (Figure S1)

To a 50 mL round-bottom flask, glacial acetic acid (30 mL) was added to a mixture of 4-hydrazino-benzenesulfonic acid (compound **1**, 3.76 g, 20 mmol), methyl isopropyl ketone (2.56 g, 30 mmol) and sodium acetate (3.20 g, 38 mmol). The suspension was refluxed under 110 °C for

24 hours, the hot solution was cooled to room temperature and concentrated, the residues were purified by silica gel column chromatography with methanol/ethyl acetate = 1/2. A pink solid (compound **3**, 4.20 g, yield 87.87%) was obtained. TLC (silica gel, EtOAc/MeOH, 2:1) $R_f = 0.4$. ^{1}H NMR (300MHz, DMSO-d_6): δ 1.24 (s, 6*H*), 2.21(s, 3*H*), 7.36 (d, *J*=9.0 Hz, 1*H*), 7.57 (d, *J*=9.0 Hz, 1*H*), 7.64 (s, 1*H*). ^{13}C NMR (75MHz, DMSO-d_6): δ 15.1, 22.4, 53.2, 118.1, 125, 145.1, 153.6, 172.5, 188.9. LRMS (*m/z*): calcd: 238.05, found: 238.97.

Synthesis of compound 4 (Figure S1)

To a 50 mL round-bottom flask, glacial acetic acid (30 mL) was added to a mixture of phenylhydrazine (compound **2**, 2.16 g, 20 mmol), methyl isopropyl ketone (2.56 g, 30 mmol) and sodium acetate (3.20 g, 38 mmol). The suspension was refluxed under 110°C for 24 hours, the hot solution was cooled to room temperature and concentrated, the residues were purified by silica gel column chromatography with ethyl acetate/petroleum ether = 1/2. A brown oil acetated salt (compound **4**, 3.26g, yield 75%) was obtained. ^{1}H NMR (300MHz, DMSO-d_6): δ 1.3 (s, 6H), 2.08 (s, 3H), 2.30 (s, 3H), 7.22 (d, *J*=9.0 Hz, 1*H*), 7.32 (m, 2*H*), 7.64 (d, *J*=6.0 Hz, 1H), 10.34 (s, 1H). ^{13}C NMR (75MHz, DMSO-d_6): δ 14.99, 23.53, 53.57, 121.33, 127.69, 145.30, 152.58, 175.62, 188.64. LRMS (*m/z*): calcd: 159.10, found: 159.06.

Synthesis of compound 5 (Figure S1)

A mixture of 2,3,3-trimethyl-3*H*-indole-5-sulfonic acid (compound **3,** 1.20 g, 5 mmol) and (3-bromopropyl)trimethyl ammonium bromide (1.56 g, 6.0 mmol) in 1,2-dichlorobenzene (16 mL) was heated at 130°C for 72 hours under argon. The mixture was cooled to room temperature and the solvent was decanted. The crude product was washed with CH_2Cl_2, dissolved in acetone and re-precipitated into a large volume of ethyl acetate to obtain a red solid (compound **5**, 1.36 g, yield 80%), which was used in the next step without further purification. NMR data was reported in a previous reference.[1a]

Synthesis of compound 6 (Figure S1)

A mixture of 2,3,3-trimethyl-3H-indole (compound **4,** 1.59 g, 10 mmol) and 1,2-oxathiane 2,2-dioxide (1.56 g, 11.50 mmol) in 1,2-dichlorobenzene (20 mL) was heated at 130°C for 48 hours under argon. The mixture was cooled to room temperature and the solvent was decanted. The crude product was extensively washed with ethyl acetate/petroleum ether = 1/2 to obtain a pink solid (compound **6**), which was used in the next step without further purification. TLC (silica gel, EtOAc/MeOH, 2:1) $R_f = 0.5$. NMR data was reported in a previous reference.[1b-c]

Synthesis of compound 7 (Figure S1)

A mixture of 2,3,3-trimethyl-3H-indole (compound **4,** 1.59 g, 10 mmol) and (3-bromopropyl) trimethyl ammonium bromide (3.0 g, 9.5 mmol) in 1,2-dichlorobenzene (20 mL) was heated at 130°C for 72 hours under argon. The mixture was cooled to room temperature and the solvent was decanted. The crude product was extensively washed with ethyl acetate/petroleum ether = 1/2 to obtain a pink solid (compound **7**), which was used in the next step without further purification. NMR data was reported in a previous reference.[1a]

Synthesis of compound ZWCY (Figure S1)

A mixture of bromide salt (compound **5**, 0.50 g, 1.48 mmol), Vilsmeier-Haack reagent 2 (0.27 g, 0.73 mmol) and anhydrous sodium acetate (0.25 g, 3.0 mmol) in 10 mL of absolute ethanol was refluxed for 6 hours under argon. The reaction mixture was cooled to room temperature, and

then concentrated under reduced pressure to yield a brownish green residue. The crude product was washed with dichloromethane. The residue was suspended in methanol/ dichloromethane (1/4, 200 mL), filtered and dried in vacuum to yield a golden-green solid (compound **ZWCY**, 505 mg, yield 84.9%). ^{1}H NMR (300 MHz, D_2O): δ 1.72 (s, 12H), 1.88 (m, 2H), 2.18 (m, 4H), 2.74 (m, 4H), 3.08 (s, 18H), 3.49 (m, 4H), 4.18 (m, 4H) m 6.37 (d, J=15 Hz, 2H), 7.45 (d, J=6 Hz, 2H), 7.69 (d, J=6 Hz, 2H) 7.85 (s, 2H), 8.31 (d, J=15 Hz, 2H). LRMS (*m/z*): calcd: 815.36, found: 815.30.

Synthesis of compound ANCY (Figure S1)

A mixture of sodium salt (compound **6**, 0.59 g, 2.0 mmol), Vilsmeier-Haack reagent (0.36 g, 1.0 mmol) and anhydrous sodium acetate (0.25 g, 3.0 mmol) in 15 mL of absolute ethanol was refluxed for 6 hours under argon. The reaction mixture was cooled to room temperature, and then concentrated under reduced pressure to yield a brownish green residue. The crude product was washed with ethyl acetate/petroleum ether = 1/2. The residues were purified by silica gel column chromatography with methanol/ethyl acetate = 1/2, a green solid (compound **ANCY**, 1.08 g, yield 75%) was yield. ^{1}H NMR (300 MHz, D_2O): δ 1.28 (s, 12H), 1.84 (m, 10H), 2.46 (s, 4H), 2.87 (s, 4H), 4.12 (s, 4H), 6.12 (d, J=12 Hz, 2H), 7.06 (m, 4H), 7.40 (s, 4H) 7.87 (d, J=15 Hz, 2H). LRMS (*m/z*): [M+2Na] calcd: 771.23, found: 771.16.

Synthesis of compound CACY (Figure S1)

A mixture of bromide salt (compound **7**, 0.52 g, 2.0 mmol), Vilsmeier-Haack reagent (0.36 g, 1.0 mmol) and anhydrous sodium acetate (0.25 g, 3.0 mmol) in 15 mL of absolute ethanol was refluxed for 6 hours under argon. The reaction mixture was cooled to room temperature, and then concentrated under reduced pressure to yield a brownish green residue. The crude product was washed with ethyl acetate/petroleum ether = 1/2. The residues were suspended in dichloromethane, filtered and dried in vacuum to yield a green solid (compound **CACY**, 0.294 g, yield 20%). ^{1}H NMR (300 MHz, D_2O): δ 1.30 (s, 12H), 2.30 (m, 4H), 2.58 (m, 4H), 3.12 (s, 18H), 3.40 (m, 2H), 3.70 (m, 4H), 4.14 (m, 4H), 6.28 (d, J=12 Hz, 2H), 7.06 (m, 2H), 7.14 (d, J=6 Hz, 2H), 7.23 (m, 4H), 8.50 (d, J=15 Hz, 2H). LRMS (*m/z*): calcd: 734.37, found: 734.96.

Synthesis of compound ABZWCY (Figure S1)

A mixture of compound ZWCY (163 mg, 0.20 mmol) and 3-(4-aminophenyl)propanoic acid (165 mg, 1.0 mmol) in DMSO was heated at 65 °C overnight. The reaction mixture was cooled to room temperature and precipitated in dichloromethane. The crude product was purified by RP C18 chromatography to yield a blue solid (compound **ABZWCY**, 104 mg, yield 55%). ^{1}H NMR (300 MHz, D_2O): δ 1.26 (s, 12H), 1.75 (m, 2H), 2.17(m, 4H), 2.38 (m, 2H), 2.51 (m, 3H), 2.80 (m, 2H), 3.14 (s, 18H), 3.48 (m, 4H), 4.01 (s, 4H), 5.98 (d, J= 18 Hz, 2H), 7.11 (m, 6H), 7.76 (m, 4H), 7.99 (d, J=15 Hz, 2H). LRMS (*m/z*): calcd: 943.25, found: 943.12.

Synthesis of compound AAZWCY (Figure S1)

A mixture of compound ZWCY (160 mg, 0.20 mmol) and 4-(2-azidoethyl) aniline (162 mg 1.0 mmol) in DMSO was heated at 65 °C overnight. The reaction mixture was cooled to room temperature and precipitated in 300 mL acetone. The crude product was filtered and purified by RP C18 chromatography to yield a blue solid (compound **AAZWCY**, 110 mg, yield 58%). ^{1}H NMR (300 MHz, D_2O): δ 1.11 (s, 12H), 1.65 (m, 3H), 2.26(m, 4H), 2.42 (m, 2H), 2.51 (m, 3H), 2.74 (m, 2H), 3.16 (s, 18H), 3.54 (m, 4H), 4.04 (s, 4H), 5.98 (s, 2H), 7.14 (m, 6H), 7.57 (m, 2H), 7.74 (m, 2H), 7.91 (d, J=12 Hz, 2H). LRMS (*m/z*): calcd: 939.46, found: 939.21.

Synthesis of compound ABANCY (Figure S1)

A mixture of compound ANCY (150 mg, 0.2 mmol) and 3-(4-aminophenyl) propanoic acid (96 mg, 0.6 mmol) in DMSO was heated at 65 °C overnight. The reaction mixture was cooled to room temperature and precipitated in ethyl acetate and washed with dichloromethane. The residues were purified by silica gel column chromatography and washed with methanol/dichloromethane= 1/2. A blue solid was obtained (compound **ABANCY**, 122 mg, yield 71%). LRMS (*m/z*): calcd: 856.37, found: 856.53.

Synthesis of compound AAANCY (Figure S1)

A mixture of compound ANCY (150 mg, 0.2 mmol) and 4-(2-azidoethyl) aniline (200 mg, 1.2 mmol) in DMSO was heated at 65 °C overnight. The reaction mixture was cooled to room temperature and precipitated in ethyl acetate and washed with dichloromethane. The product was dried to yield a blue solid (compound **AAANY**) without further purification (60 mg, yield 35.3%). LRMS (*m/z*): [M+2Na] calcd: 897.34, found: 897.35.

Synthesis of compound ABCACY (Figure S1)

A mixture of compound CACY (215 mg, 0.29 mmol) and 3-(4-aminophenyl) propanoic acid (139 mg, 0.84 mmol) in DMSO was heated at 65 °C overnight. The reaction mixture was cooled to room temperature and precipitated in ethyl acetate and washed with dichloromethane. The product was dried to yield 145 mg (yield 58 %) of a blue solid (compound **ABCACY**) without further purification. ^{1}H NMR (300 MHz, D_2O): δ 1.27 (s, 4H), 1.72 (s, 2H), 2.00(m, 12H), 2.28 (m, 4H), 2.56 (m, 2H), 3.14 (m, 18H), 3.42 (m, 6H), 3.68(m, 2H), 4.11 (m, 2H), 6.00 (d, J= 15, 2H), 7.04 (d, J= 6, 2H), 7.10 (m, 4H), 7.16 (m, 4H), 7.58 (d, J= 6 Hz, 2H), 8.04 (d, J= 15 Hz, 2H). LRMS (*m/z*): calcd: 863.47, found: 863.71.

Synthesis of compound AACACY (Figure S1)

A mixture of compound CACY (310 mg, 0.38 mmol) and 4-(2-azidoethyl) aniline (320 mg, 2 mmol) in DMSO was heated at 65 °C overnight. The reaction mixture was cooled to room temperature and precipitated in ethyl acetate and washed with dichloromethane. The product was dried to yield 200 mg (yield 61 %) of a blue solid (compound **AACACY**) without further purification. ^{1}H NMR (300 MHz, D_2O): δ 1.36 (s, 4H), 1.75 (s, 2H), 2.15(m, 12H), 2.30 (m, 4H), 2.62 (m, 2H), 3.12 (m, 18H), 3.46 (m, 6H), 3.62(m, 2H), 4.16 (m, 2H), 6.04 (d, J= 15 Hz, 2H), 6.73 (d, J= 9 Hz, 2H), 7.16 (m, 6H), 7.22 (m, 4H), 8.06 (d, J= 15 Hz, 2H). LRMS (*m/z*): calcd: 860.48, found: 860.91.

Synthesis of compound Propynyl-HPβCD (Figure S2)

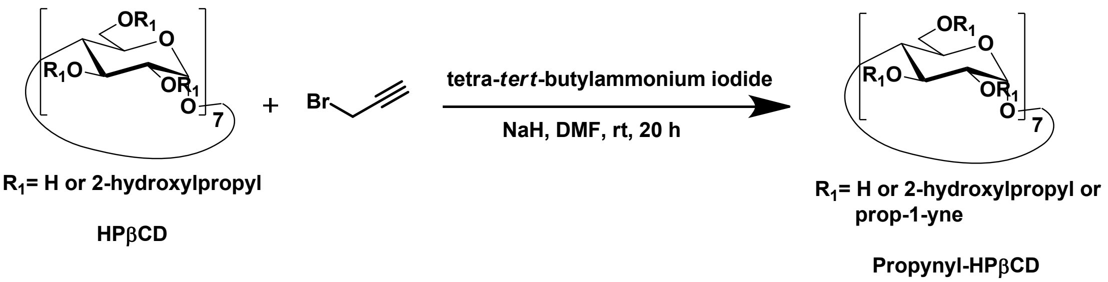

Figure S2. Schematic (A) and Synthesis route (B) of compound Propynyl-HPβCD.

A suspension of sodium hydride (0.8 g, 20 mmol, 60% dispersion in mineral oil) in anhydrous DMF (10 mL) was added dropwise to a solution of (2-hydroxypropyl)-β-cyclodextrin (3.08 g, 2.0 mmol) and tetra-tert-butylammonium iodide (0.16 g, 0.44 mmol) in anhydrous DMF (15 mL) at 0 °C. After being stirred for 0.5 hour at 0 °C, a solution of propargyl bromide (0.6 g, 4 mmol) in 1 mL of anhydrous DMF was added dropwise. The reaction mixture was allowed to stir at room temperature for 24 hours while the solution turned brown. The mixture was concentrated under reduced pressure. The crude product was purified by column chromatography (Methanol/ethylacetate = 1/2, Methanol/H_2O = 2/1,) to obtain 3.1 g of a grey solid compound Propynyl-HPβCD after freeze dry.

Synthesis of compound 4-(2-azidoethyl) aniline (Figure S3)

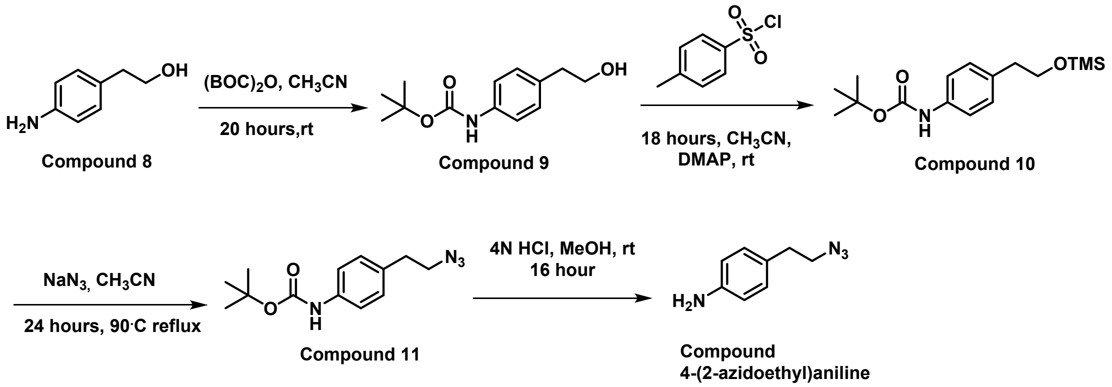

Figure S3. Synthesis of precursor compound 4-(2-azidoethyl) aniline.

Synthesis of compound 9 (Figure S3)

To a dry 50 mL round-bottom flask equipped with a stir bar under N_2 were added: $(BOC)_2O$ (4.4 g, 20.2 mmol), 2-(4-aminophenyl)ethanol (compound **8**, 2.74 g, 20 mmol) and 20 mL CH_3CN. The resulting mixture was stirred at room temperature overnight. The solvent is concentrated under reduced pressure in a rotary evaporator. The crude product was purified by gel silica chromatographic column with eluent (ethyl acetate/petroleum ether = 1/2, R_f =0.3), the eluent was collected concentrated under vacuum and freeze dried to yield a white solid (compound **9**, 4.62 g, yield 97.5%). ^1H NMR (300 MHz, $CDCl_3$): δ 1.51 (s, 9H), 2.81 (t, J=12 Hz, 2H), 3.80 (m, 2H), 6.51 (s, 1H), 7.15 (d, J=9 Hz, 2H), 7.31 (d, J=9 Hz, 2H). ^{13}C NMR (75 MHz, $CDCl_3$): δ 28.35, 38.50, 63.70, 80.49, 118.97, 129.52, 133.10, 136.80, 152.89.

Synthesis of compound 10 (Figure S3)

To a solution of (compound **9**, 2.37 g, 10 mmol) in CH_3CN (20 mL), 4-Toluenesulfonyl chloride (2.56 g, 13.4 mmol), 4-Dimethylaminopyridine (122 mg, 1 mmol) and triethylamine (1.5 mL) were added and stirred at room temperature for 48 hours. The solvent was concentrated under reduced pressure in a rotary evaporator. The residue was purified by gel silica chromatographic column with eluent (ethyl acetate/petroleum ether = 1/3, R_f =0.5), the eluent was collected and concentrated under vacuum to yield a colorless oil (compound **10**, 3.82 g, yield 96.7%). ^1H NMR (300 MHz, $CDCl_3$): δ 1.51 (s, 9H), 2.43 (s, 3H), 2.89 (t, J=15 Hz, 3H), 4.16 (t, J=12 Hz, 3H), 6.47 (s, 1H), 6.99 (d, J=9 Hz, 2H), 7.26 (m, 4H), 7.69 (d, J=9 Hz, 2H). ^{13}C NMR (75 MHz, CDCl3): δ 21.61, 28.34, 34.69, 50.87, 70.70, 80.57, 118.71, 127.83, 129.78, 132.97, 144.67, 152.73.

Synthesis of compound 11 (Figure S3)

To a solution of (compound **10**, 3.13 g, 8 mmol) in DMF (15 mL), NaN_3 (0.676 g, 10.4 mmol), was added and refluxed at 90°C for 20 hours. The solvent was concentrated under reduced pressure in a rotary evaporator. After cooling down, distilled water (75 mL) was added and a white solid (crude compound **11**) was precipitated. The mixture was extensively washed and extracted by using diethyl ether. The diethyl ether layer was dried with anhydrous magnesium sulphate and concentrated under reduced vacuum to yield purity product yellow oil (compound **11**) without further purification (2.12 g, yield 96%, ethyl acetate/petroleum ether = 1/3, R_f=0.67).

Synthesis of compound 4-(2-azidoethyl) aniline (Figure S3)

To a solution of compound **11** (2 g, 7.63 mmol) in CH_2Cl_2 (10 mL), trifluoroacetic acid (5 mL) was added and stirred at room temperature for 6 hour. The solvent was concentrated under reduced pressure in a rotary evaporator. The residue was purified by gel silica chromatographic column with eluent (ethyl acetate/ petroleum ether =2/1, R_f =0.3), the eluent was collected concentrated under vacuum to yield a colorless oil compound **4-(2-azidoethyl) aniline**. LRMS (*m/z*): calcd: 162.09, found: 162.05.

Synthesis of compounds cyanine-HPβCD (Figure S4)

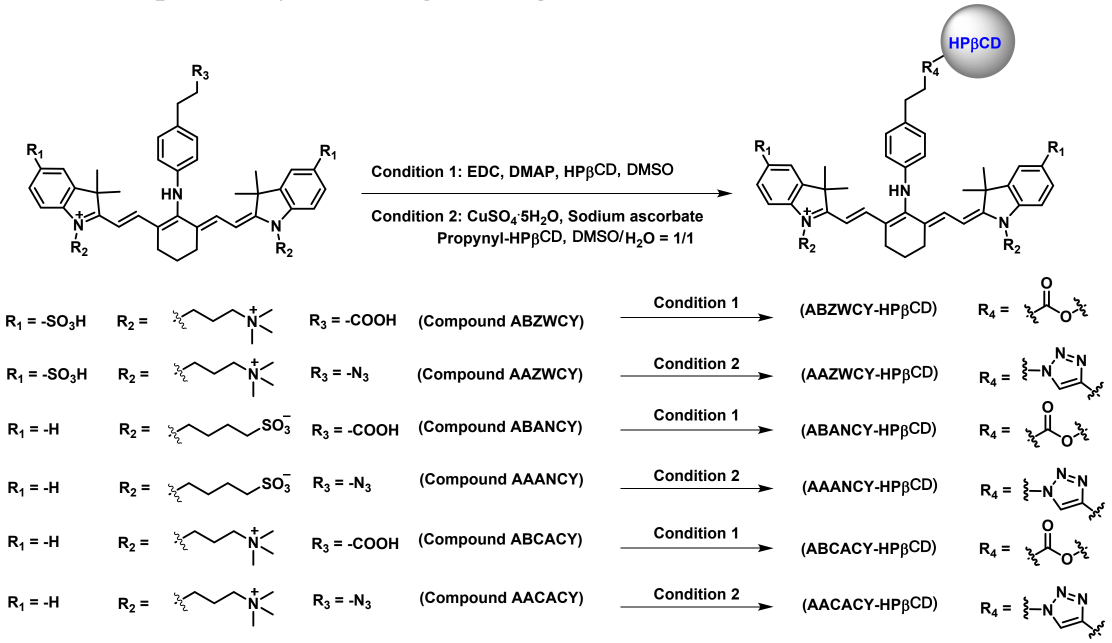

Figure S4. Synthesis of near infrared Cyanine-HPβCD agents.

Synthesis of compound ABZWCY-HPβCD (Figure S4)

A mixture of dye ABZWCY (47 mg, 0.05 mmol), 1-ethyl-3-(3-dimethyl- aminopropyl) carbodiimide (48 mg, 0.25 mmol), 4-dimethylaminopyridine (10 mg, 0.064 mmol) and HPβCD (2 g, 1.3 mmol) and DMSO (8 mL) was stirred under argon at room temperature for 12 hours. The reaction mixture was then precipitated in 350 ml ethyl acetate. The crude product was filtered and further purified by Sephadex G-25 to yield a blue solid (compound **ABZWCY-HPβCD**, 1.6 g). ^{1}H-NMR; ^{13}C-NMR and Mass data are available in Figure S41-43. Another three batches were performed in the same procedure. ^{1}H NMR (300 MHz, D_2O): δ 1.01 (m, 838H), 1.21 (s, 12H), 1.70 (m, 2H), 2.11 (m, 4H), 2.34 (m, 4 H), 2.62 (s, 2H), 2.84 (s, 2H), 2.98 (s, 18H),

3.26-4.0 (m, 3336H), 4.10 (t, 4H), 4.62 (m, 1510H), 4.92-5.08 (m, 180H), 5.86 (d, 2H), 6.80-7.20 (m, 6H), 7.50-7.70 (m, 4H), 7.84 (d, J=9 Hz, 2H). ^{13}C NMR (300 MHz, D_2O): δ 19.10, 19.18, 19.44, 24.37, 27.72, 30.10, 34.60, 43.75, 48.44, 54.09, 61.38, 67.31, 72.64, 72.91, 73.16, 74.01, 82.12, 100.69, 102.77, 109.81, 118.50, 120.02, 124.75, 126.65, 130.05, 133.89, 140.02, 142.46, 147.39, 160.70, 170.10, 173.97.

Synthesis of compound AAZWCY-HPβCD (Figure S4)
A mixture of AAZWCY (46 mg, 0.05 mmol) and the appropriate propynyl cyclodextrin (2 g, 1.25 mmol) was dissolved in a DMSO and water mixture (1:1). A solution of sodium ascorbate (228 mg, 1.1 mmol) in water, followed by a solution $CuSO_4 \cdot 5H_2O$ (100 mg, 0.6 mmol) in water was added. The mixture was stirred overnight at room temperature under argon gas protection and exclusion of light. After 12 hours, this mixture was then precipitated in 350 ml acetone. The crude product was filtered and further purified by Sephadex G-25 to yield a blue solid **(AAZWCY-HPβCD**, 1.56 g). ^{1}H-NMR; ^{13}C-NMR and Mass data are available in Figure S44-46. The other batch was performed in the same procedure. ^{1}H NMR (300 MHz, D_2O): δ 1.01 (m, 1103H), 1.21 (s, 12H), 1.59 (m, 2H), 2.12 (m, 4H), 2.39 (m, 4 H), 2.59 (s, 2H), 2.82 (s, 55H), 2.99 (s, 18H), 3.20-4.0 (m, 3856H), 4.10 (t, 67H), 4.25 (s, 95H), 4.61(m, 1703H), 4.92-5.09 (m, 174H), 5.89 (s, 2H), 6.71 (s, 1H), 6.80-7.20 (m, 6H), 7.50 (m, 2H), 7.54 (m, 2H), 7.82 (d, J=9 Hz, 2H). ^{13}C NMR (300 MHz, D_2O): 19.13, 19.21, 19.48, 24.73, 26.82, 31.55, 34.12, 45.80, 54.06, 57.50, 61.36, 67.26, 72.53, 77.22, 77.76, 79.14, 81.17, 100.14, 101.38, 102.81, 110.40, 119.03, 121.36, 123.57, 126.55, 131.51, 133.59, 141.84, 143.21, 147.23, 159.10, 170.07.

Synthesis of compound ABANCY-HPβCD (Figure S4)
A mixture of dye ABANCY (45 mg, 0.052 mmol), 1-ethyl-3- (3-dimethylaminopropyl)carbodiimide (35 mg, 0.18 mmol), 4-dimethyl-amino pyridine (10 mg, 0.08 mmol) and HPβCD (600 mg, 0.39 mmol) and DMSO (6 mL) was stirred at room temperature under argon gas protection and exclusion of light. After 12 hours the reaction mixture was then precipitated in ethyl acetate. The crude product was further purified by Sephadex G-25 to yield a blue solid (**ABANCY-HPβCD**, 350 mg). ^{1}H-NMR and Mass data are available in Figure S47-48. ^{1}H NMR (300 MHz, D_2O): δ 1.01 (m, 420H), 1.21 (s, 12H), 1.72-1.77 (m, 10H), 2.42 (t, 2H), 2.59 (m, 4 H), 2.73-2.76 (t, 6H), 3.21-4.0 (m, 1295H), 4.08 (t, 4H), 4.64 (m, 527H), 4.95-5.11 (m, 134H), 5.84 (s, 2H), 6.78-7.20 (m, 12H), 7.84 (d, J=9 Hz, 2H).

Synthesis of compound AAANCY-HPβCD (Figure S4)
A mixture of AAANCY (44 mg, 0.05 mmol) and the appropriate propynyl cyclodextrin (710 mg, 0.44 mmol) was dissolved in a DMSO and water mixture (1:1). A solution of sodium ascorbate (60 mg, 0.3 mmol) in water, followed by a solution of $CuSO_4 \cdot 5H_2O$ (16 mg, 0.1 mmol) in water was added. The mixture was stirred overnight at room temperature under argon gas protection and exclusion of light. After overnight this mixture was then precipitated in 300 mL acetone. The crude product was filtered and further purified by Sephadex G-25 to yield a blue solid (**AAANCY-HPβCD**, 500 mg). ^{1}H-NMR and Mass data are available in Figure S49-50. ^{1}H NMR (300 MHz, D_2O): δ 1.01 (m, 238H), 1.19 (s, 12H), 1.72-1.77 (m, 10H), 2.49 (t, 4H), 2.69 (m, 2 H), 2.75-2.83 (m, 13H), 3.21-4.0 (m, 848H), 4.08-4.13 (m, 11H), 4.26 (s, 22H), 4.64 (m, 460H), 4.94-5.10 (m, 93H), 5.78 (s, 2H), 6.80-7.70 (m, 13H), 7.73 (d, J=9 Hz, 2H).

Synthesis of compound ABCACY-HPβCD (Figure S4)

A mixture of dye ABCACY (45 mg, 0.052 mmol), 1-ethyl-3- (3-dimethylaminopropyl)carbodiimide (35 mg, 0.18 mmol), 4-dimethylaminopyridine (10 mg, 0.08 mmol) and HPβCD (600 mg, 0.39 mmol) and DMSO (6 mL) was stirred at room temperature under argon gas protection and exclusion of light. The reaction mixture was then precipitated in ethyl acetate. The crude product was further purified by Sephadex G-25 to yield a blue solid (**ABCACY-HPβCD**, 350 mg). ^{1}H-NMR and Mass data are available in Figure S51-52. ^{1}H NMR (300 MHz, D_2O): δ 1.01 (m, 26H), 1.21 (s, 12H), 1.75 (m, 2H), 1.94 (m, 4H), 2.60 (m, 4H), 2.74 (t, 2H), 2.83 (t, 2H), 3.09 (s, 18H), 3.25-3.9 (m, 122H), 4.08 (t, 2H), 4.61(m, 58H), 4.95-5.11 (m, 11H), 5.87 (d, J=9 Hz, 2H), 6.52-7.30 (m, 12H), 7.88 (d, J=9 Hz, 2H).

Synthesis of compound AACACY-HPβCD (Figure S4)

A mixture of AACACY (77 mg, 0.09 mmol) and the appropriate propynyl cyclodextrin (1 g, 0.625 mmol) was dissolved in a DMSO and water mixture (1:1). A solution of sodium ascorbate (80 mg, 0.4 mmol) in water, followed by a solution $CuSO_4 \cdot 5H_2O$ (32 mg, 0.2 mmol) in water was added. The mixture was stirred overnight at room temperature under argon gas protection and exclusion of light. After 12 hours, this mixture was then precipitated in 300 mL acetone. The crude product was filtered and further purified by Sephadex G-25 to yield a blue solid (**AACACY-HPβCD**, 440 mg). ^{1}H-NMR and Mass data are available in Figure S53-54.
^{1}H NMR (300 MHz, D_2O): δ 1.01 (m, 896H), 1.21 (s, 12H), 1.90 (m, 2H), 2.04 (m, 4H), 2.56 (m, 4 H), 2.81 (s, 20H), 2.96 (t, 2H), 3.04 (s, 18H), 3.24-4.0 (m, 3510H), 4.09 (t, 37H), 4.24 (s, 71H), 4.61 (m, 2123H), 4.92-5.08 (m, 385H), 5.89 (s, 2H), 6.50-7.30 (m, 13H), 7.90 (d, J=9 Hz, 2H).

3. Optical properties characterization

Stock solutions of all the compounds were prepared and stored at -20 °C. All the spectroscopic measurements were conducted in phosphate buffered saline (PBS). UV-vis and fluorescence spectra were acquired using a microplate reader (Tecan Infinite M200) or an Eppendorf biospectrometer kinetic device. All measurements were conducted at 25 °C. Extinction coefficients of fluorophores were determined by using 20 μM solutions in aqueous buffer and calculated based on the Lambert-Beer law. For fluorescence quantum yield (QY) measurements, oxazine 725 in ethylene glycol (QY = 19%) and ICG in DMSO (QY =13%) were used as a calibration standards, under conditions of matched absorbance at 655 and 765 nm.

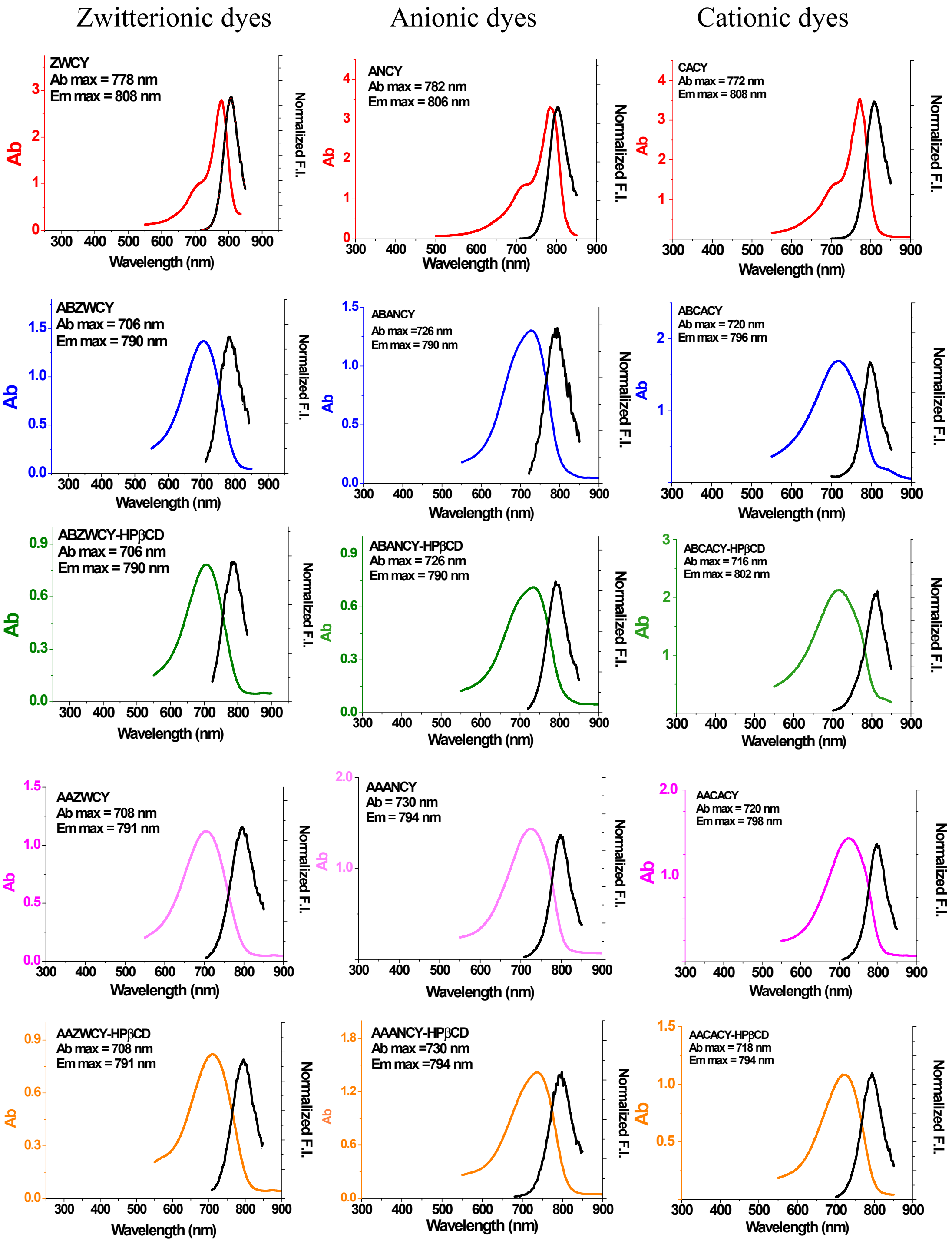

Figure S5. Absorption and emission spectra of each of the markers in 1xPBS; ZWCY, ANCY and CACY (0.05 mg/mL); ABZWCY, AAZWCY, ABANCY, AAANCY, ABCACY and AACACY (0.02 mg/mL); ABZWCY-HPβCD, AAZWCY-HPβCD, ABANCY-HPβCD, AAANCY-HPβCD, ABCACY-HPβCD and AACACY-HPβCD (2 mg/mL). Note: the peak sized of fluorescence spectra are arranged by the peak values of absorbance spectra to show together as a single pair for clear observation of the Stokes-shift.

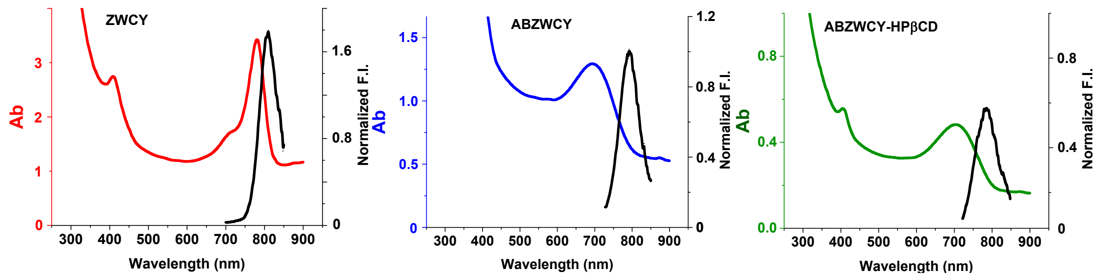

Figure S6. Absorption and emission spectra of ZWCY (0.05 mg/mL), ABZWCY (0.02 mg/mL) and ABZWCY-HPβCD (1 mg/mL) in plasma. Note: the peak sized of fluorescence spectra are arranged by the peak values of absorbance spectra to show together as a single pair for clear observation of the Stokes-shift.

4. Degree of labeling (DOL)[2]

The degree of labeling (DOL) is the average number of dye molecules coupled to HPβCD. The DOL can be determined from the absorption spectrum of a marker against the corresponding free dye standard solution of known concentration.[2] The calibration curves were performed in a series of known concentrations of free dyes. Dye stock solution 0.05 mg/ml ABZWCY were prepared in PBS. It was diluted in a concentration of 0.01, 0.009, 0.008, 0.007, 0.006, 0.005, 0.004, 0.003, 0.002 and 0.001 mg/mL. For AAZWCY, a dye stock solution of 0.15 mg/mL was prepared in PBS. It was diluted in a concentration of 0.015, 0.0135, 0.012, 0.0105, 0.009, 0.0075, 0.06, 0.0045, 0.003 and 0.0015 mg/mL. Their corresponding UV absorption was measured and calibration curves were constructed based on the UV absorption values. Subsequently, absorbances of ABZWCY-HPβCD and AAZWCY-HPβCD with corresponding concentration were measured, and their degree of labeling was calculated based on the calibration curves and the equation S1.

$$\text{DOL}=\frac{C1}{MW1}\times\frac{(MW2\ +MW1\times DOL)}{C2}\qquad\text{(Equation S1)}$$

Where C1 is the concentration of the dye labeled in HPβCD, MW1 is the molecular weight of dye, MW2 is the average molecular weight of HPβCD, C2 is the concentration of markers (dye conjugated with HPβCD), DOL is the value of degree of labeling.

Table S1. A_{uv} is the absorption value of free dye ABZWCY in the constant concentration.

**C (mg/ml)**	0.01	0.009	0.008	0.007	0.006	0.005	0.004	0.003	0.002	0.001
A_{uv}	0.23	0.205	0.1857	0.1669	0.1476	0.1280	0.1093	0.0816	0.069	0.0479

Table S2. A_{uv} is the absorption value of free dye AAZWCY in the constant concentration.

**C (mg/ml)**	0.015	0.0135	0.012	0.0105	0.009	0.0075	0.006	0.0045	0.003	0.0015
A_{uv}	0.4184	0.3658	0.3304	0.2922	0.2678	0.236	0.1925	0.1590	0.1163	0.0795

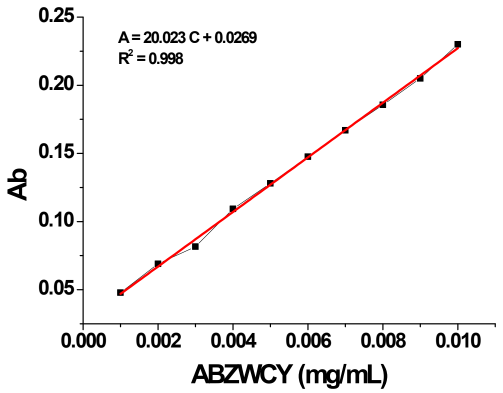

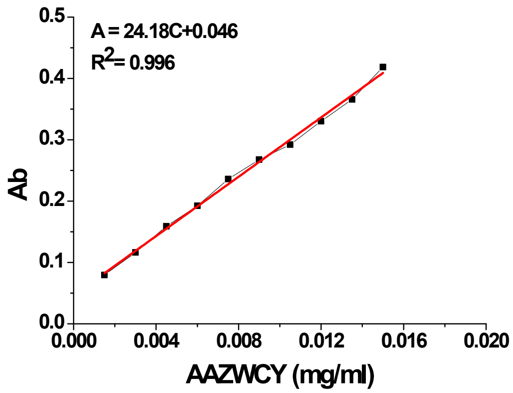

Figure S7. Linear calibration curve of ABZWCY and AAZWCY.

Table S3a. Degree of labeling for different batches of ABZWCY-HPβCD and AAZWCY-HPβCD.

**Marker (1 mg/mL)**	**ABZWCY-HPβCD**				**AAZWCY-HPβCD**	
Batch	Batch 1	Batch 2	Batch 3	Batch 4	Batch 1	Batch 2
Absorbance	0.46	0.43	0.51	0.44	0.65	0.60
DOL	0.037	0.034	0.04	0.033	0.042	0.039

To ensure the reliability of the DOL measurement, the absorbance and fluorescent intensity were measured for HPβCD based agents (ABZWCY-HPβCD and AAZWCY-HPβCD), free dye mixed with free HPβCD (with the same ratio of DOL), and only free dye (with the same ratio of DOL). Absorbance values are closed in all the three measurements either in PBS or in plasma. Similar results were also observed in their fluorescent intensity. Those results indicated the conjugated HPβCD doesn't contribute the absorbance of ABZWCY-HPβCD and AAZWCY-HPβCD, demonstrate the DOL measurement is reliability.

Table S3b. Absorbance at 708 nm was measured on HPβCD based agents (ABZWCY-HPβCD and AAZWCY-HPβCD), free dye mixed with free HPβCD with the same ratio of DOL, and only free dye.

Marker	ABZWCY-HPβCD (DOL= 0.033) 0.75 mg/mL	The mixture of ABZWCY and HPβCD (0.025/0.725 mg/mL, 3.3%/96.7%)	ABZWCY (0.025mg/mL, 3.3%)	AAZWCY-HPβCD (DOL= 0.042) 0.75 mg/mL	The mixture of AAZWCY and HPβCD (0.03/0.72mg/mL, 4%/96%)	AAZWCY (0.03mg/mL, 4%)
Absorbance in **PBS**	0.308	0.312	0.316	0.448	0.456	0.461
Absorbance in **Plasma**	0.459	0.472	0.476	0.559	0.572	0.578

Table S3c. Fluorescent intensity at 790 nm was measured on HPβCD based agents (ABZWCY-HPβCD and AAZWCY-HPβCD), free dye mixed with free HPβCD with the same ratio of DOL, and only free dye. (ex = 708 nm)

Marker	ABZWCY-HPβCD (DOL= 0.033) 0.75 mg/mL	The mixture of ABZWCY and HPβCD (0.025/0.725 mg/mL, 3.3%/96.7%)	ABZWCY (0.025mg/mL, 3.3%)	AAZWCY-HPβCD (DOL= 0.042) 0.75 mg/mL	The mixture of AAZWCY and HPβCD (0.03/0.72mg/mL, 4%/96%)	AAZWCY (0.03mg/mL, 4%)
Fluroescent intensity in **PBS**	397	400	411	717	727	734
Fluroescent intensity in **Plasma**	458	459	451	741	754	757

5. Plasma protein binding (PPB)[3, 4, 5] and dynamic light scattering analysis

A dye-protein stock solution was prepared by incubating 2 mL of the corresponding dye or agent (1 mg/mL) with 8 mL Sprague Dawley rat plasma in Li-Heparin (Innovative Research, Novi, MI, USA) in PBS at 37°C, while 2 mL PBS was incubated with 8 mL rat plasma as control. PPB measurements were performed by equilibrium dialysis of PBS against dye-protein stock solution (or control stock solution) using a two-chamber dialysis set-up. After 24 hours the absorption of each of the dyes or agents in PBS and plasma were determined in three independent measurements by both absorption spectroscopy and fluorescence spectroscopy in a microplate reader. The concentrations of each of the dyes or agents were calculated on the basis of the corresponding molar absorption coefficients. All experiments were performed in triplicate. PPB in percent [%] was determined by averaging three independent measurements and following the equation of Lambert Beer law:

PPB= [A(plasma) - A(PBS)]/[A(plasma) + A(PBS)] x 100% (Equation S2),

where A is corresponding to UV absorption.

ABZWCY-HPβCD (1 mg/mL in PBS), SD rat plasma (50% in PBS), ABZWCY-HPβCD (1 mg/mL) mixed with SD rat plasma (50% in PBS) were prepared and filtered through a sterile 0.22 μm filter before analysis. Dynamic light scattering (DLS) studies were conducted. The Zetasizer Nano S90 uses a 633 nm helium-neon laser. The measurements were carried out in quadratic cells and analyzed the scattered light at an angle of 90° at a controlled temperature (25 °C). The intensity of scattering of a particle is assumed to be proportional to the sixth power of its diameter. The apparent hydrodynamic radius was calculated according to the Stokes-Einstein equation.

PPB of ABZWCY

UV-vis absorption measurement

	Control group			Sample group			Result
	Plasma+ PBS	Plasma+ PBS	Plasma+ PBS	Plasma + ABZWCY	Plasma + ABZWCY	Plasma + ABZWCY	Sample-Control
Plasma	0.2077	0.1905	0.1907	0.2695	0.2492	0.2488	
Average		0.1963			0.2558		0.0595
PBS	0.0564	0.0491	0.0495	0. 0836	0.0961	0.0966	
Average		0.0516			0.0921		0.0405
PPB			(0.0595-0.0405)/(0.0595+0.0405) x100%				19%

Fluorescent intensity measurement

	Control group			Sample group		
	Plasma+ PBS	Plasma+ PBS	Plasma+ PBS	Plasma + ABZWCY	Plasma + ABZWCY	Plasma + ABZWCY
Plasma	2	8	6	343	370	359
PBS	1	5	4	113	112	84

PPB of the mixture of ABZWCY and HPβCD (5%:95%)

UV-vis absorption measurement

	Control group			Sample group			Result
	Plasma+ PBS	Plasma+ PBS	Plasma+ PBS	Plasma + ABZWCY Mixed HPβCD	Plasma + ABZWCY Mixed HPβCD	Plasma + ABZWCY Mixed HPβCD	Sample-Control
Plasma	0.0733	0.0748	0.0719	0.250	0.249	0.248	
Average		0.073			0.249		0.176
PBS	0.0406	0.040	0.0407	0.165	0.164	0.161	
Average		0.040			0.163		0.123
PPB			(0.176-0.123)/(0.176+0.123) x100%				17.7 %

Fluorescent intensity measurement

	Control group			Sample group		
	Plasma+ PBS	Plasma+ PBS	Plasma+ PBS	Plasma + ABZWCY Mixed HPβCD	Plasma + ABZWCY Mixed HPβCD	Plasma + ABZWCY Mixed HPβCD
Plasma	8	5	5	2762	2719	2670
PBS	0	3	1	781	735	704

PPB of ABZWCY-HPβCD

UV-vis absorption measurement

	Control group			Sample group			Result
	Plasma+ PBS	Plasma+ PBS	Plasma+ PBS	Plasma + ABZWCY-HPβCD	Plasma + ABZWCY-HPβCD	Plasma + ABZWCY-HPβCD	Sample-Control
Plasma	0.2077	0.1905	0.1907	0.2559	0.2626	0.2619	
Average		0.1963			0.2622		0.0613
PBS	0.0564	0.0491	0.0495	0. 1024	0.1103	0.1123	
Average		0.0516			0.1113		0.0586
PPB			(0.0613-0.0586)/(0.0613+0.0586) x100%				3.7 %

Fluorescent intensity measurement

	Control group			Sample group		
	Plasma+ PBS	Plasma+ PBS	Plasma+ PBS	Plasma + ABZWCY-HPβCD	Plasma + ABZWCY-HPβCD	Plasma + ABZWCY-HPβCD
Plasma	1	8	9	1429	1713	1726
PBS	0	-1	-1	1124	1472	1409

PPB of AAZWCY

UV-vis absorption measurement

	Control group			Sample group			Result
	Plasma+ PBS	Plasma+ PBS	Plasma+ PBS	Plasma + AAZWCY	Plasma + AAZWCY	Plasma + AAZWCY	Sample-Control
Plasma	0.1608	0.160	0.159	0.443	0.447	0.444	
Average		0.160			0.445		0.0285
PBS	0.0501	0.0503	0.0507	0. 257	0.258	0.258	
Average		0.0504			0.258		0.208
PPB			(0.0285-0.0208)/(0.0285+0.0208) x100%				15.7%

Fluorescent intensity measurement

	Control group			Sample group		
	Plasma+ PBS	Plasma+ PBS	Plasma+ PBS	Plasma + AAZWCY	Plasma + AAZWCY	Plasma + AAZWCY
Plasma	12	10	5	304	384	359
PBS	3	3	4	94	96	89

PPB of the mixture of AAZWCY and HPβCD (5%:95%)

UV-vis absorption measurement

	Control group			Sample group			Result
	Plasma+ PBS	Plasma+ PBS	Plasma+ PBS	Plasma + AAZWCY Mixed HPβCD	Plasma + AAZWCY Mixed HPβCD	Plasma + AAZWCY Mixed HPβCD	Sample-Control
Plasma	0.0733	0.0748	0.0719	0.233	0.237	0.233	
Average		0.073			0.234		0.161
PBS	0.0406	0.040	0.0407	0.161	0.163	0.162	
Average		0.040			0.162		0.122
PPB			(0.161-0.122)/(0.161+0.122) x100%				13.7 %

Fluorescent intensity measurement

	Control group			Sample group		
	Plasma+ PBS	Plasma+ PBS	Plasma+ PBS	Plasma + AAZWCY Mixed HPβCD	Plasma + AAZWCY Mixed HPβCD	Plasma + AAZWCY Mixed HPβCD
Plasma	8	5	5	2365	2481	2354
PBS	0	3	1	654	713	654

PPB of AAZWCY-HPβCD

UV-vis absorption measurement

	Control group			Sample group			Result
	Plasma+ PBS	Plasma+ PBS	Plasma+ PBS	Plasma + AAZWCY-HPβCD	Plasma + AAZWCY-HPβCD	Plasma + AAZWCY-HPβCD	Sample-Control
Plasma	0.2320	0.2288	0.2270	0.2794	0.2747	0.2749	
Average		0.2290			0.276		0.047
PBS	0.0468	0.0457	0.0460	0. 0859	0.0916	0.0841	
Average		0.0461			0.0873		0.0412
PPB			(0.047-0.0412)/(0.047+0.0412) x100%				6.5 %

Fluorescent intensity measurement

	Control group			Sample group		
	Plasma+ PBS	Plasma+ PBS	Plasma+ PBS	Plasma + AAZWCY-HPβCD	Plasma + AAZWCY-HPβCD	Plasma + AAZWCY-HPβCD
Plasma	7	5	7	1423	1516	1521
PBS	4	3	2	1233	1372	1342

PPB of ABANCY

UV-vis absorption measurement

	Control group			Sample group			Result
	Plasma+ PBS	Plasma+ PBS	Plasma+ PBS	Plasma + ABANCY	Plasma + ABANCY	Plasma + ABANCY	Sample-Control
Plasma	0.216	0.2031	0.211	0.3118	0.3173	0.3315	
Average		0.210			0.3202		0.1102
PBS	0.0418	0.0438	0.0409	0.0469	0.0443	0.0423	
Average		0.0421			0.0445		0.0024
PPB			(0.1102-0.0024)/(0.1102+0.0024) x100%				95 %

Fluorescent intensity measurement

	Control group			Sample group		
	Plasma+ PBS	Plasma+ PBS	Plasma+ PBS	Plasma + ABANCY	Plasma + ABANCY	Plasma + ABANCY
Plasma	-1	1	-1	1136	1178	1139
PBS	0	-2	1	1	2	1

PPB of ABANCY-HPβCD

UV-vis absorption measurement

	Control group			Sample group			Result
	Plasma+ PBS	Plasma+ PBS	Plasma+ PBS	Plasma + ABANCY-HPβCD	Plasma + ABANCY-HPβCD	Plasma + ABANCY-HPβCD	Sample-Control
Plasma	0.217	0.226	0.226	0.269	0.286	0.286	
Average		0.223			0.280		0.057
PBS	0.0441	0.0467	0.0456	0.0988	0.0758	0.077	
Average		0.0455			0.0837		0.0382
PPB			(0.057-0.0382)/(0.057+0.0382) x100%				19.7 %

Fluorescent intensity measurement

	Control group			Sample group		
	Plasma+ PBS	Plasma+ PBS	Plasma+ PBS	Plasma + ABANCY-HPβCD	Plasma + ABANCY-HPβCD	Plasma + ABANCY-HPβCD
Plasma	1	0	2	1243	1378	1344
PBS	3	0	8	202	215	205

PPB of AAANCY

UV-vis absorption measurement

	Control group			Sample group			Result
	Plasma+ PBS	Plasma+ PBS	Plasma+ PBS	Plasma + AAANCY	Plasma + AAANCY	Plasma + AAANCY	Sample-Control
Plasma	0.236	0.231	0.221	0.301	0.290	0.277	
Average		0.230			0.289		0.0594
PBS	0.0443	0.0436	0.0429	0.0496	0.0501	0.0510	
Average		0.0436			0.0502		0.0066
PPB			(0.0594-0.0066)/(0.0594+0.0066) x100%				80 %

Fluorescent intensity measurement

	Control group			Sample group		
	Plasma+ PBS	Plasma+ PBS	Plasma+ PBS	Plasma + AAANCY	Plasma + AAANCY	Plasma + AAANCY
Plasma	5	7	-4	1136	1168	1237
PBS	0	4	3	10	20	11

PPB of AAANCY-HPβCD

UV-vis absorption measurement

	Control group			Sample group			Result
	Plasma+ PBS	Plasma+ PBS	Plasma+ PBS	Plasma + AAANCY-HPβCD	Plasma + AAANCY-HPβCD	Plasma + AAANCY-HPβCD	Sample-Control
Plasma	0.1735	0.1894	0.1912	0.323	0.305	0.279	
Average		0.184			0.280		0.118
PBS	0.0432	0.0422	0.0416	0.108	0.1114	0.1072	
Average		0.042			0.11		0.068
PPB				(0.118-0.068)/(0.118+0.068) x100%			26.8 %

Fluorescent intensity measurement

	Control group			Sample group		
	Plasma+ PBS	Plasma+ PBS	Plasma+ PBS	Plasma + AAANCY-HPβCD	Plasma + AAANCY-HPβCD	Plasma + AAANCY-HPβCD
Plasma	0	0	1	2168	2154	2032
PBS	2	-1	-1	283	292	283

PPB of ABCACY

UV-vis absorption measurement

	Control group			Sample group			Result
	Plasma+ PBS	Plasma+ PBS	Plasma+ PBS	Plasma + ABCACY	Plasma + ABCACY	Plasma + ABCACY	Sample-Control
Plasma	0.142	0.144	0.145	0.163	0.161	0.158	
Average		0.143			0.160		0.017
PBS	0.0407	0.0402	0.0405	0.044	0.0462	0.0458	
Average		0.0405			0.0455		0.005
PPB				(0.017-0.005)/(0.017+0.005) x100%			54.5 %

Fluorescent intensity measurement

	Control group			Sample group		
	Plasma+ PBS	Plasma+ PBS	Plasma+ PBS	Plasma + ABCACY	Plasma + ABCACY	Plasma + ABCACY
Plasma	3	5	6	568	563	560
PBS	2	1	3	29	25	20

PPB of ABCACY-HPβCD

UV-vis absorption measurement

	Control group			Sample group			Result
	Plasma+ PBS	Plasma+ PBS	Plasma+ PBS	Plasma + ABCACY-HPβCD	Plasma + ABCACY-HPβCD	Plasma + ABCACY-HPβCD	Sample-Control
Plasma	0.142	0.144	0.145	0.2247	0.2205	0.2219	
Average		0.143			0.2223		0.0793
PBS	0.0407	0.0402	0.0405	0.089	0.0892	0.0906	
Average		0.0405			0.0896		0.0491
PPB			(0.0793-0.0491)/(0.0793+0.0491) x100%				23.5 %

Fluorescent intensity measurement

	Control group			Sample group		
	Plasma+ PBS	Plasma+ PBS	Plasma+ PBS	Plasma + ABCACY-HPβCD	Plasma + ABCACY-HPβCD	Plasma + ABCACY-HPβCD
Plasma	3	5	6	892	824	877
PBS	2	1	3	660	635	626

PPB of AACACY

UV-vis absorption measurement

	Control group			Sample group			Result
	Plasma+ PBS	Plasma+ PBS	Plasma+ PBS	Plasma + AACACY	Plasma + AACACY	Plasma + AACACY	Sample-Control
Plasma	0.142	0.144	0.145	0.1664	0.1638	0.1612	
Average		0.143			0.1638		0.0208
PBS	0.0407	0.0402	0.0405	0.0474	0.0462	0.0436	
Average		0.0405			0.0457		0.0052
PPB			(0.0208-0.0052)/(0.0208+0.0052) x100%				60 %

Fluorescent intensity measurement

	Control group			Sample group		
	Plasma+ PBS	Plasma+ PBS	Plasma+ PBS	Plasma + AACACY	Plasma + AACACY	Plasma + AACACY
Plasma	3	5	6	665	615	590
PBS	2	1	3	36	40	26

PPB of AACACY-HPβCD

UV-vis absorption measurement

	Control group			Sample group			Result
	Plasma+ PBS	Plasma+ PBS	Plasma+ PBS	Plasma + AACACY-HPβCD	Plasma + AACACY-HPβCD	Plasma + AACACY-HPβCD	Sample-Control
Plasma	0.142	0.144	0.145	0.2232	0.2264	0.2279	
Average		0.143			0.2258		0.0828
PBS	0.0407	0.0402	0.0405	0.0872	0.0884	0.0873	
Average		0.0405			0.0876		0.0471
PPB				(0.0828-0.0471)/(0.0828+0.0471) x100%			27.4 %

Fluorescent intensity measurement

	Control group			Sample group		
	Plasma+ PBS	Plasma+ PBS	Plasma+ PBS	Plasma + AACACY-HPβCD	Plasma + AACACY-HPβCD	Plasma + AACACY-HPβCD
Plasma	3	5	6	994	953	926
PBS	2	1	3	578	631	606

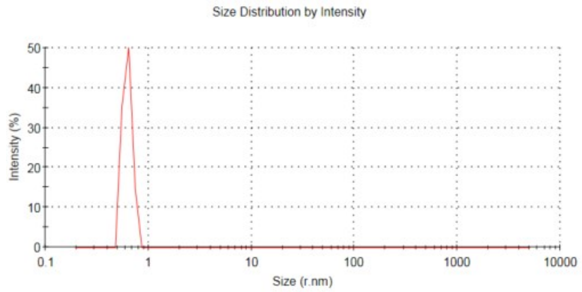

ABZWCY-HPβCD (1 mg/mL)

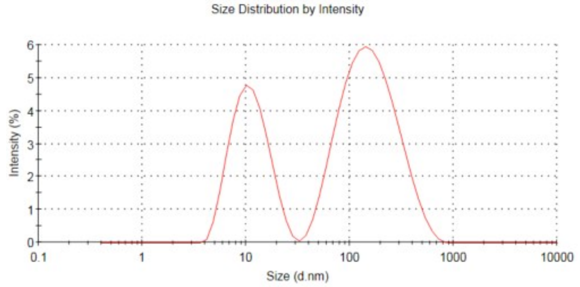

SD rat plasma (50% in PBS)

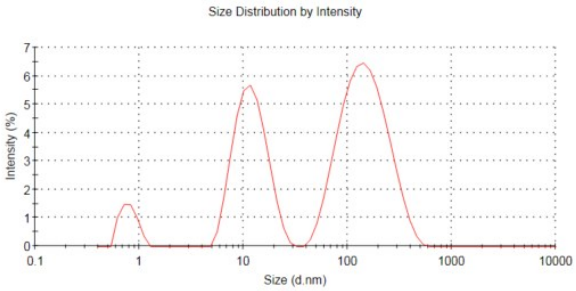

ABZWCY-HPβCD (1 mg/mL) mixed with SD rat plasma (50% in PBS)

Figure. S8. Size distribution by intensity.

Table S4 Results of DLS measurements.

Substance	PDI	Peak 1 [nm]	Peak 2 [nm]	Peak 3 [nm]
ABZWCY-HPβCD	0.37	0.63	0	0
Plasma protein	0.94	0	11.60	178.2
ABZWCY-HPβCD mixed with Plasma protein	0.89	0.80	12.31	159.7

PDI, polydispersity index.

6. Stability studies in porcine liver esterase and purity investigation in HPLC

Porcine liver esterase (PLE, 10 mg/mL, 150 unit/mL) was added to ABZWCY-HPβCD (10 mg/mL) or AAZWCY-HPβCD (5 mg/mL) in sodium phosphate buffer solution (pH 7.5-8.0). This mixture was incubated at 37 °C for 24 hours. After incubation it was filtered via sterile syringe filter with a membrane pore size of 0.22 µm and transferred to a proper vial. Samples of three control groups such as PLE, native fluorophore and the corresponding marker were prepared according to the same procedure. Esterase enzymatic degradation was monitored by injecting 50 µL of each sample into HPLC. The gradient program is described in Table S5.

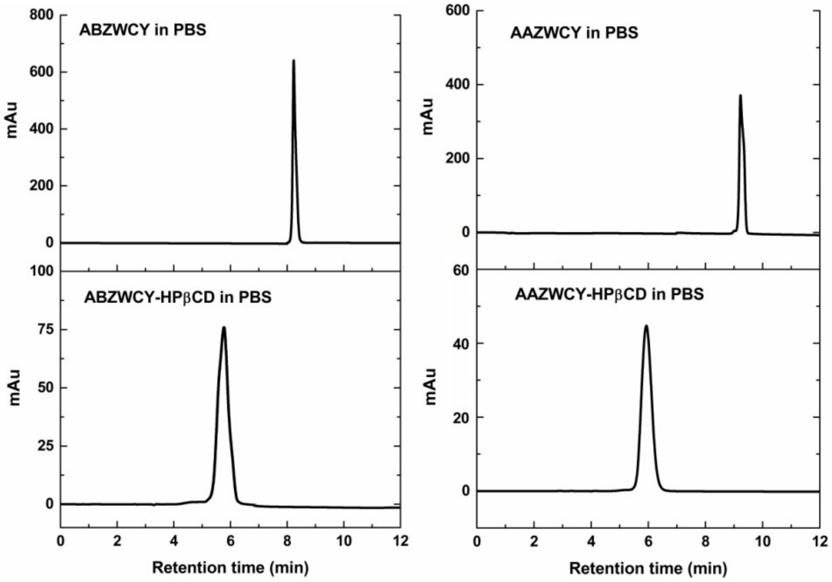

Figure. S9. High performance liquid chromatography (monitored at 254 nm) of ABZWCY, ABZWCY-HPβCD, AAZWCY, and AAZWCY-HPβCD in PBS.

Table S5 Gradient program for HPLC.

Instrument	Thermo scientific ultimate 3000 liquid chromatography
Column	Ascentis® C18 columns, 5 µm, 250 x 21.2 mm, 25 °C
Needle wash	0.1% concentrated H_3PO_4 in MeOH
Seal wash solution	H_2O:CH_3CN (1 : 9)
Detector	A photodiode array detector (UltiMate™ DAD 3000 detector)
Flow rate	1 mL/min
Injection volume	50 µL
Absorbance	254 nm and 710 nm
Gradient program	Solution A: 0.1% formic acid in H_2O;<br>Solution B: 0.1% formic acid in acetonitile<br>0 - 10 min, 90% to 10% H_2O<br>10 - 12 min, 10% to10% H_2O

7. Cell viability evaluated by MTT assay.

The cytotoxicity of ABZWCY-HPβCD and AAZWCY-HPβCD were evaluated by using standard (4,5-dimethyl-2-thiazolyl)-2,5-diphenyltetrazolium bromide (MTT) assay in a 96-well plate set up to assess the viability of cultured cells. HK-2 human proximal tubular cell is an immortalized proximal tubule cell line from normal adult human kidney. Cells were cultured and incubated for 3 hours at 37 °C with various concentrations of ABZWCY-HPβCD and AAZWCY-HPβCD (0.0125, 0.025, 0.05, 0.1 mg/mL) in Hank's balanced salt solution (HBSS; Gibco). After the treatment cells were washed 2 times with HBSS and incubated with 5 mg/mL MTT, freshly prepared in HBSS for 3 hours. After MTT incubation cells were washed 2 times with HBSS and 100 µL of dimethyl sulfoxide (DMSO) was added to the wells (in order to dissolve MTT crystals). The plates were then placed in an orbital shaker for a minimum of 30 min and subsequently absorbance was measured at 570 nm. The cell viability was calculated as the ratio of the absorbance of the sample to that of the control cells and expressed as a percentage. All experiments were performed in triplicate.

8. Principle and methods for transcutaneous measurements of kidney function

The transcutaneous assessment of renal function is based on the measurement of the fluorescence signal of a marker through the skin. A miniaturized transcutaneous device is comprised of two light emitting diodes (LEDs) and one photodiode (Figure S10a and 10b). The LEDs can blink every few seconds to excite a fluorescent marker using excitation wavelength of 700 nm. The fluorescent signal will be recorded by detecting the emission intensity of a fluorescent marker at 790 nm. Data are stored in the device and can be read out after measurement.[6] The clearance half-life of fluorescent agents was calculated by software, which was developed by the Institute of Medical Technology of the University of Heidelberg.[7] For this a 3-exponential function was fitted to the measured elimination curve, the peak of the curve was supposed to be 100%. Also a 1-exponential function was applied from 50% to 15% of the peak height.

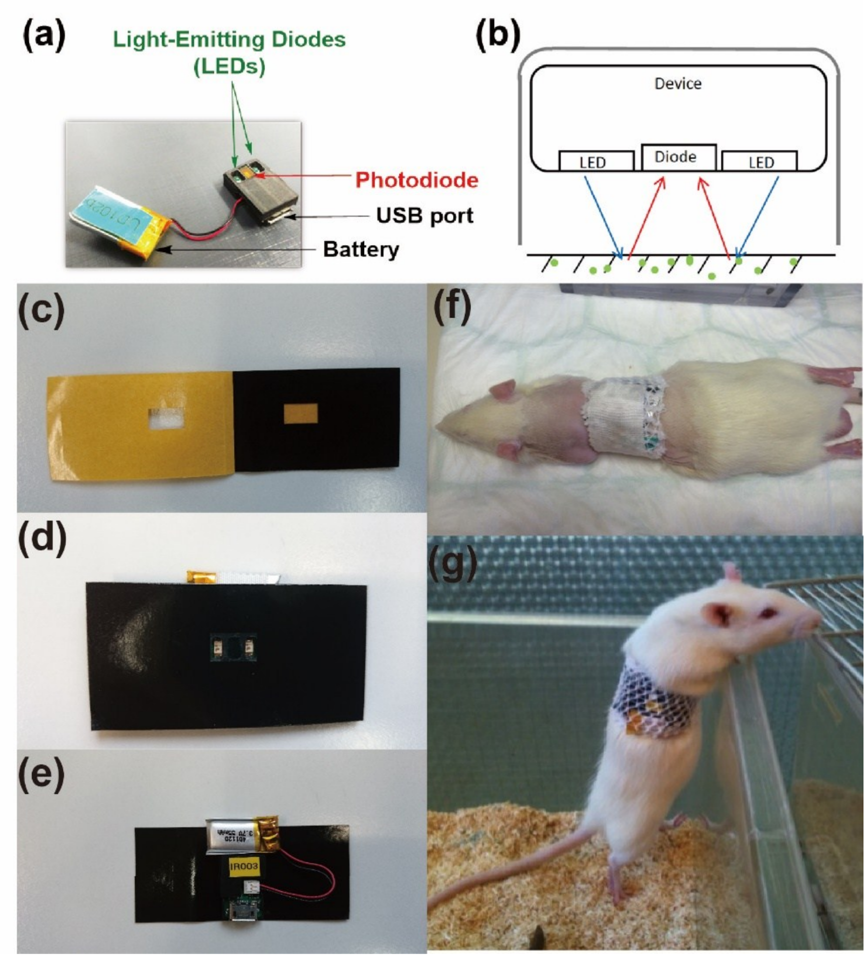

Figure. S10. (a) Transcutaneous device and battery used for the transcutaneous measurement, the photodiodes and LEDs in the device. (b) A schematic overview of the excitation and emission light between a transcutaneous device and fluorescent agents in capillary vessel after injection. (c-g) The fixation procedures of a transcutaneous device on the skin and measurements in a conscious rat.

Healthy Sprague Dawley rats (Body weight: 250-300 g, 8-10 weeks) were anesthetized for a short period with Isoflurane (Forene®, AbbVie, Illinois, USA; Dosage: 5 %; Flow(O_2): 5 L/min) in order to fix the transcutaneous device on the back of rats. The back of the animals was depilated with an electric shaver (Figure 10c-g). After a baseline measurement for around 5 min, a fluorescent agent in saline (DeltaSelect, GmbH, Rimbach, Germany) was injected as a bolus by tail vein injection. The dosages of fluorescent agents are depended on fluorescent quantum yield and degree of labeling of each agent (IRDye800CW: 0.1 mg/kg, ABZWCY: 2.5 mg/kg, ABZWCY mixed with HPβCD: 2.5 mg/kg mixed with 22.5 mg/kg (HPβCD), ABZWCY-HPβCD and ABZWCY-HPβCD: 50 mg/kg). Rats were conscious during the measurement and housed in separate cages. The devices were removed and the data were read out after 120 min of transcutaneous measurement. The clearance half-life of fluorescent agents was calculated by software. In probenecid or cimetidine inhibition studies, Sprague Dawley rats were treated in the same manner as described above. The rats group received 50 mg/kg probenecid or cimetidine intraperitoneally injection 30 min prior to injection of the test agents. Conversion of clearance half-life into GFR can be performed if needed. The method is based on our previous studies.[8, 9]

All experiments were conducted in accordance with the German Animal Protection Law and approved by the local authority (Regierungspräsidium Nordbaden, Karlsruhe Germany in agreement with EU guideline 2010/63/EU).

9. Bio-distribution studies by small animal imaging

Three healthy Sprague Dawley rats (Body weight 550 g) were anesthetized for a short period with Isoflurane (Forene®, AbbVie, Illinois, USA; Dosage: 5 %; Flow(O_2): 5 L/min). For the control rat, saline with the same volume of agents was injected intravenously. ABZWCY-HPβCD and AAZWCY-HPβCD (dosage, 50 mg/kg) were injected intravenously. After 5 hours, rats were scarified and fluorescence imaging was performed by using small animal imaging (PerkinElmer, excitation light at 700 nm, emission wavelength at 780 nm). For each experiment, camera exposure time and image normalization were held constant.

10. Urinary recovery of injected dose in urine studies

Recovery of the injected dose in urine studies were conducted in conscious, healthy Sprague Dawley rats (Body weight: 250-300 g). The corresponding test agents with corresponding dosage (ABZWCY: 2.5 mg/kg, ABZWCY mixed with HPβCD: 2.5 mg/kg mixed with 22.5 mg/kg (HPβCD), ABZWCY-HPβCD and AAZWCY-HPβCD: 50 mg/kg) were administered by tail vein injection. Urine was collected using metabolic cages in intervals of 1, 2, 3, 6, 9, 24 h after intravenous injection of agents into rats. The samples were stored at -20°C until they were analyzed. The urine samples were centrifuged for 8 min at 13000 ×g and then filtered by 0.22 µm syringe filter. A series of working solution of each agent with concentrations between 0.02 mg/mL and 2 mg/mL were prepared. Appropriate volumes of the working solutions were added to blank urine and fluorescent intensity of those mixtures were measured in order to obtain an external calibration curve. Quantification of each of the agents in urine at each time point was performed via HPLC analysis and fluorescence intensity detection. The concentration in urine at each time point was calculated based on the external calibration standards curve between fluorescence intensity and the concentration of each of the agents.

Considering biological components within urine may interfere with the fluorescent intensity of agents, the fluorescent intensity of agent were tested to study whether there is difference in urine and PBS, the emission intensity and calibration curve are compared (Figure S11). The results indicated that the value in urine is a little bit higher than in PBS due to solvent effect, and exhibited a slightly difference from 3% to 4%, while it is acceptable. This slightly difference can explained the urinary recoveries have some trial a little over 100% (Table S6 and S7), which can be found in other literatures studies.[10]

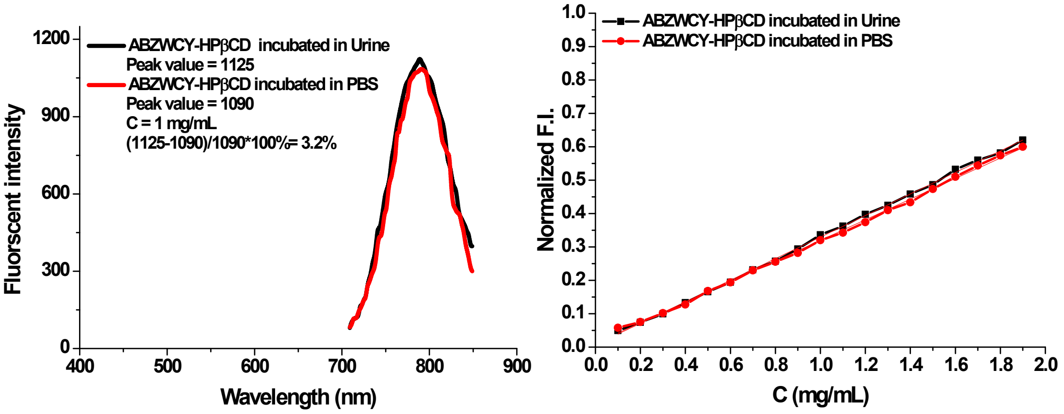

Figure S11. Fluorescent intensity and calibration curve of ABZWCY-HPβCD in Urine and PBS.

Table S6 Urinary recovery of ABZWCY (n=3) and ABZWCY mixed with HPβCD (n=3) within 24 hours.

Time (h)	Urinary recovery of ABZWCY [%]			Urinary recovery of ABZWCY mixed with HPβCD [%]		
**0**	0	0	0	0	0	0
**1**	46.67	53.33	49.29	48.7	30	
**2**	65	73.06				
**3**				66.2	68	81
**6**	87.22	81.95	89.05	73.5	82.3	
**9**		86.39	96.94			
**24**	98.33	93.3	106.6	93	91.2	105.3
**Mean±SD**		**99.4 ± 4.8**			**96.5 ± 5.8**	

Table S7 Urinary recovery of ABZWCY-HPβCD (n=3) and AAZWCY-HPβCD (n=3) within 24 hours.

Time (h)	Urinary recovery of ABZWCY-HPβCD [%]			Urinary recovery of AAZWCY-HPβCD [%]		
**0**	0	0	0	0	0	0
**1**						74.2
**2**	59	61.8	63.3	75.23	59.5	90.65
**3**		82.2			75	101.3
**6**	92.4		80.5	103.83	88	
**9**	99.9	90.10	86.1	105.45	95	102.33
**24**	103	96.4	91.7	108	97	105
**Mean±SD**		**97 ± 3.8**			**103.3 ± 4.2**	

11. Determination of urinary metabolites

Urine samples were collected and stored at -20 °C until analysis. Urine samples were centrifuged for 8 min at 13000×g and then filtered through a 0.22 μm syringe filter. The filtered urine samples were determined by HPLC on a Thermo scientific ultimate 3000 liquid chromatography using Ascentis® C18 columns (5 μm, 250 x 21.2 mm). The injection volume was 50 μL. The gradient program is described in Table S5. Select portions of the eluent were collected based on the processed HPLC signal and measured by a MALDI-TOF mass spectrometer.

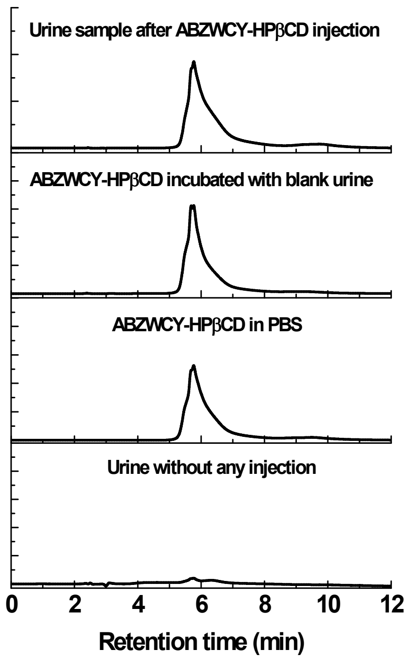
Urine sample after ABZWCY-HPβCD injection
ABZWCY-HPβCD incubated with blank urine
ABZWCY-HPβCD in PBS
Urine without any injection
0 2 4 6 8 10 12
Retention time (min)

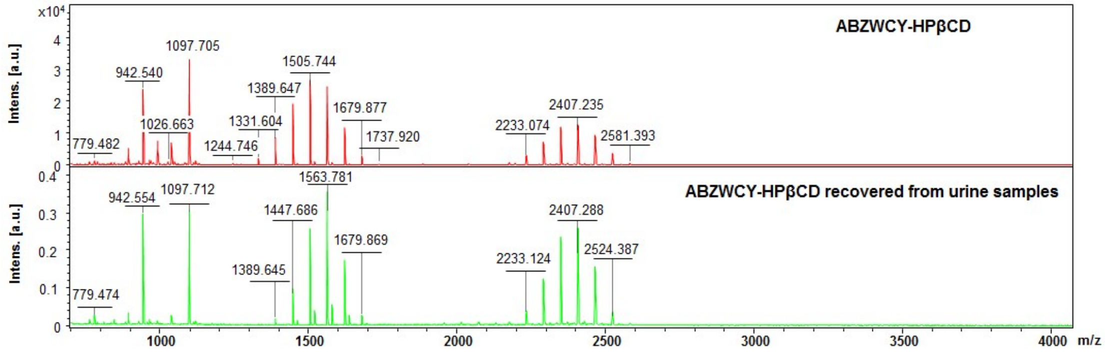
ABZWCY-HPβCD
x10^4
Intens. [a.u.]
1097.705
942.540
1505.744
1389.647
1679.877
1026.663
1331.604
1244.746
779.482
1737.920
2233.074
2407.235
2581.393
ABZWCY-HPβCD recovered from urine samples
Intens. [a.u.]
942.554
1097.712
1563.781
1447.686
1679.869
1389.645
779.474
2233.124
2407.288
2524.387
1000 1500 2000 2500 3000 3500 4000 m/z

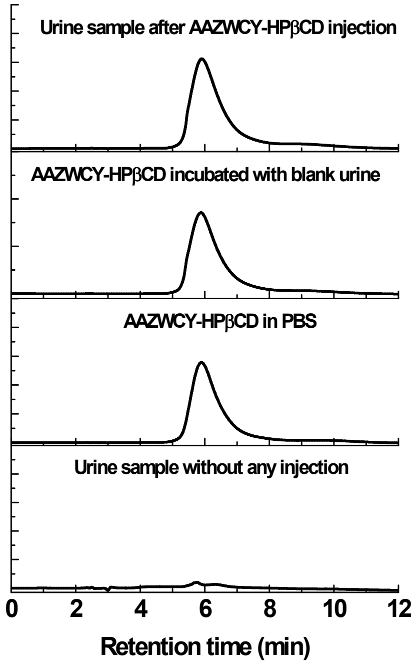
Urine sample after AAZWCY-HPβCD injection
AAZWCY-HPβCD incubated with blank urine
AAZWCY-HPβCD in PBS
Urine sample without any injection
0 2 4 6 8 10 12
Retention time (min)

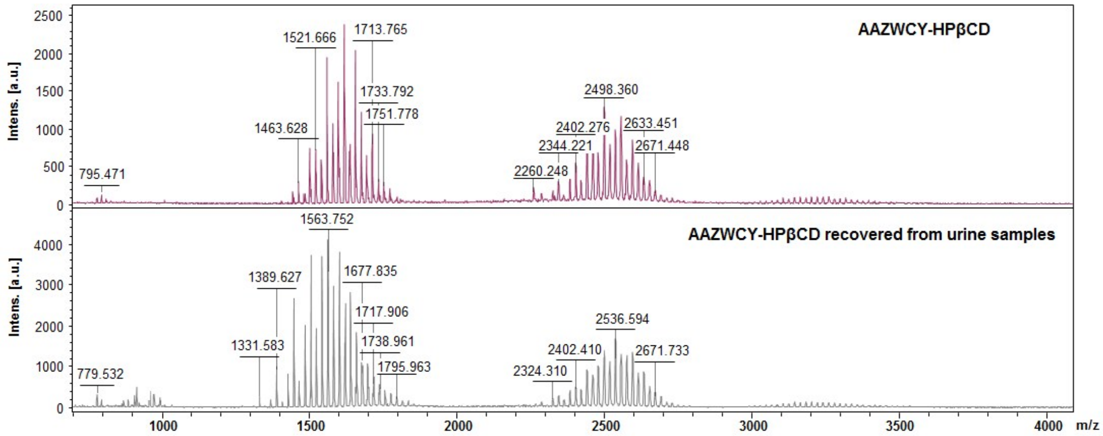

Figure S12. Evaluation of metabolites of ABZWCY-HPβCD and AAZWCY-HPβCD (monitored at 710 nm) in urine samples using HPLC assay (gradient program in table S5) and MALDI-TOF.

12. Transcutaneous measurements of kidney function in a nephropathy rat model

A transgenic rats (TGR) model with overexpression of the human Ang II type 1 receptor (hAT1) in podocytes was used.[11] In this nephropathy model, the damage progressed to nephron loss via focal segmental glomerulosclerosis, leading to the degeneration of both glomerulus and tubule.[11] Urine was collected using metabolic cages in 16 hours. The urine samples were centrifuged for 8 min at 13000 ×g and stored at -20°C until they were analyzed. The method of transcutaneous measurement of kidney function in transgenic rats is the same with that in healthy rats aforementioned (Content 8 and Figure S10). Briefly, transgenic rats or age-matched wild type rats (Body weight: 550-600 g, 24-25 weeks) were anesthetized for a short period with Isoflurane (Forene®, AbbVie, Illinois, USA; Dosage: 5 %; Flow(O_2): 5 L/min) in order to fix the transcutaneous device on the back of rats. After a baseline measurement for around 5 min, NIR agent ABZWCY-HPβCD (50 mg/kg) in saline was injected as a bolus by tail vein injection. Rats were conscious during the measurement and housed in separate cages. The devices were removed and the data were read out after 120 min of transcutaneous measurement. The clearance half-life of fluorescent agents was calculated by software. Conversion of clearance half-life into GFR can be performed if needed, the method is based on our previous studies.[8, 9] All experiments were conducted in accordance with the German Animal Protection Law and approved by the local authority (Regierungspräsidium Nordbaden, Karlsruhe Germany in agreement with EU guideline 2010/63/EU).

13. NMR and mass spectra

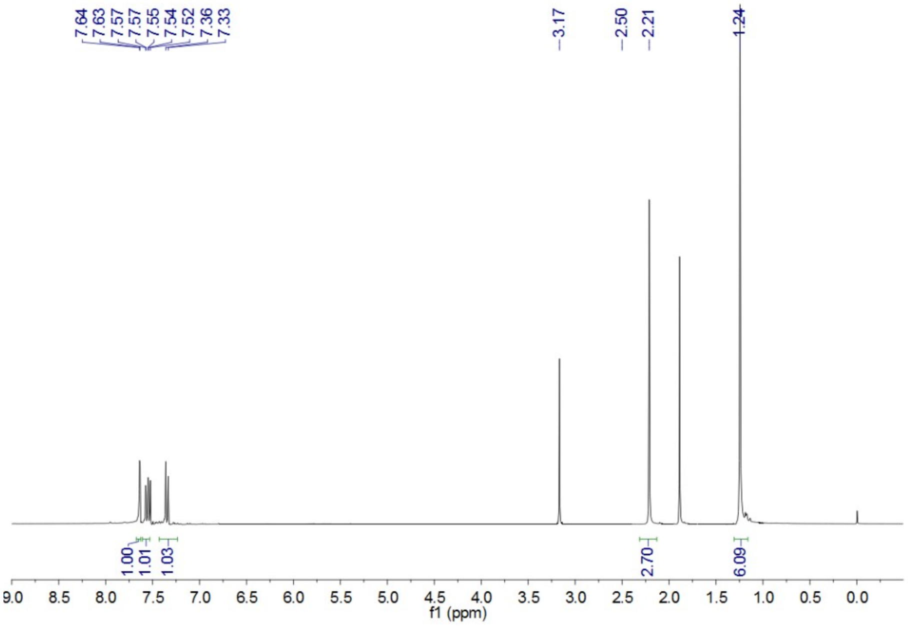

Figure S13. ^{1}H NMR of Compound 3 in DMSO-d_6

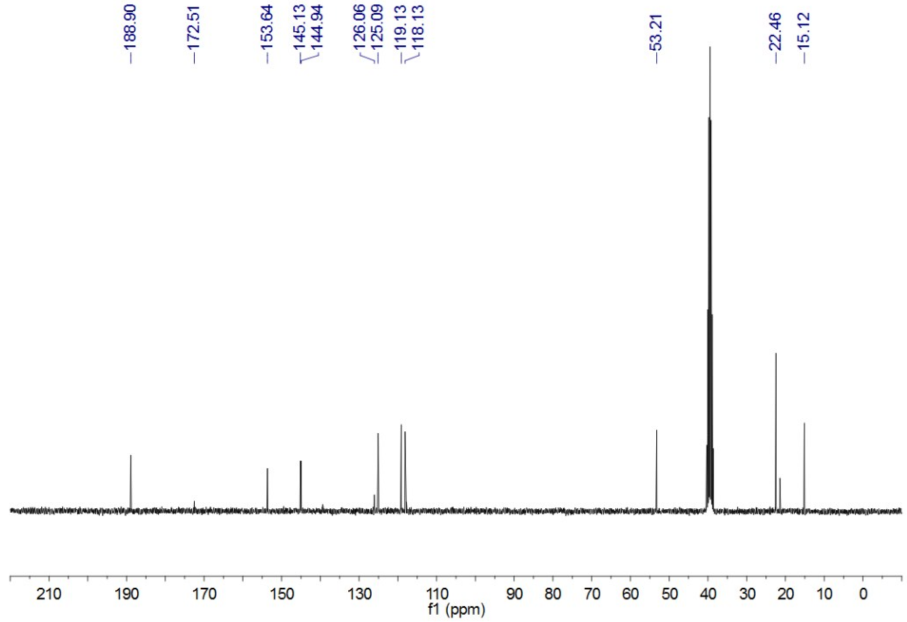

Figure S14. ^{13}C NMR of Compound 3 in DMSO-d_6

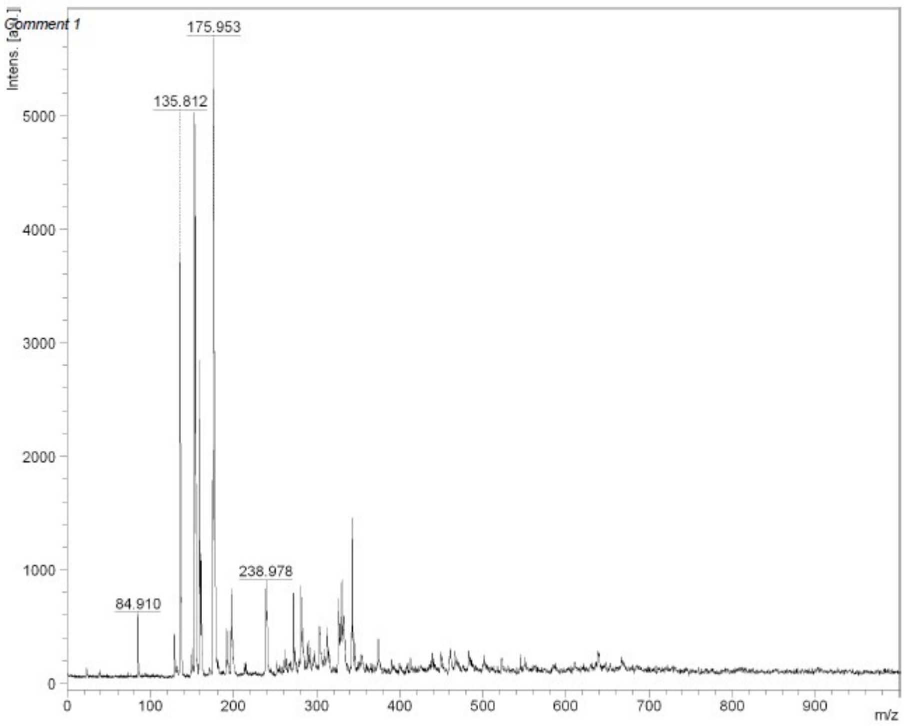

Figure S15. Mass spectra of Compound 3

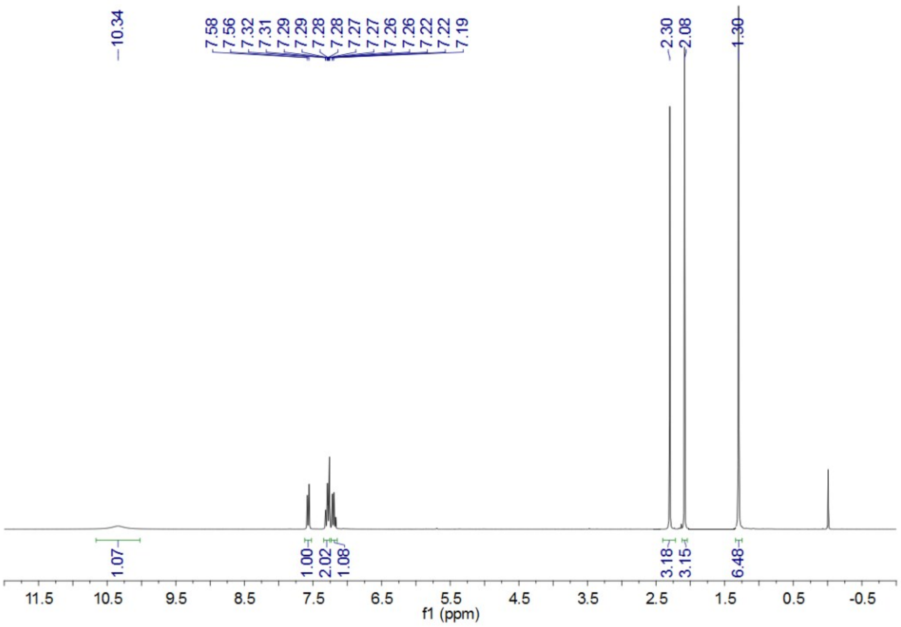

Figure S16. ^{1}H NMR of Compound 4 in DMSO-d_6

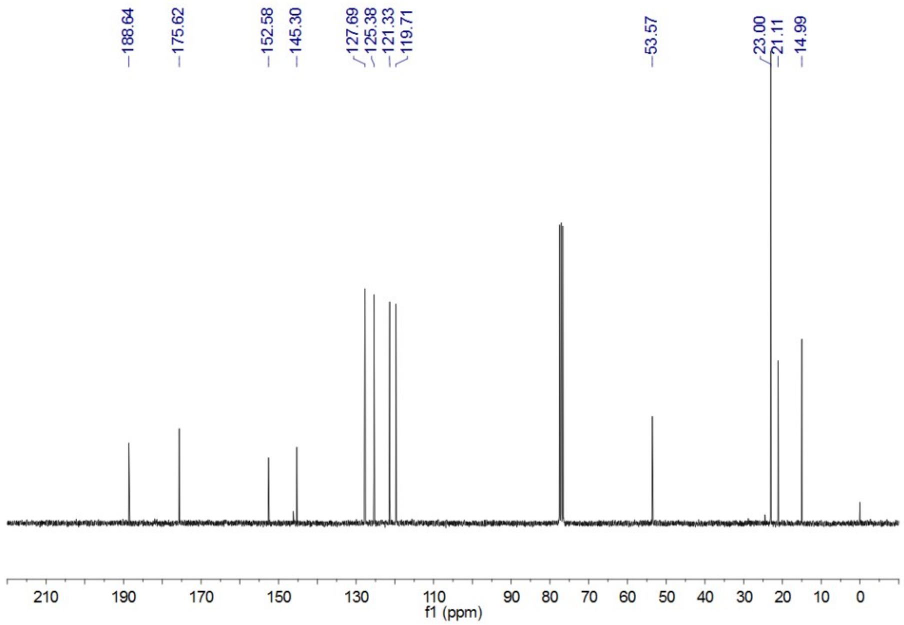

Figure S17. ^{13}C NMR of Compound 4 in DMSO-d_6

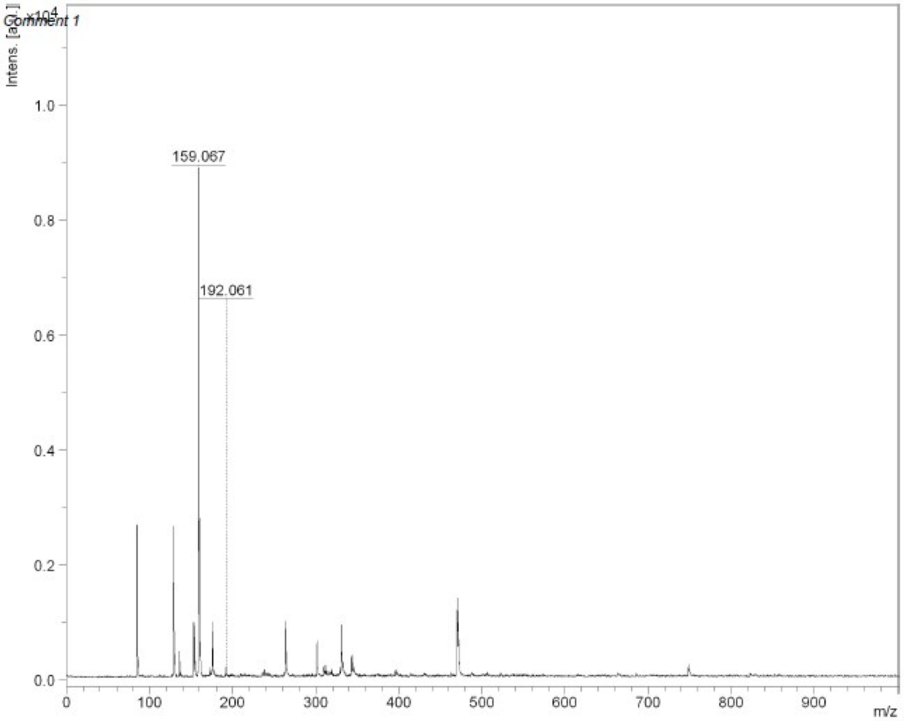

Figure S18. Mass spectra of Compound 4

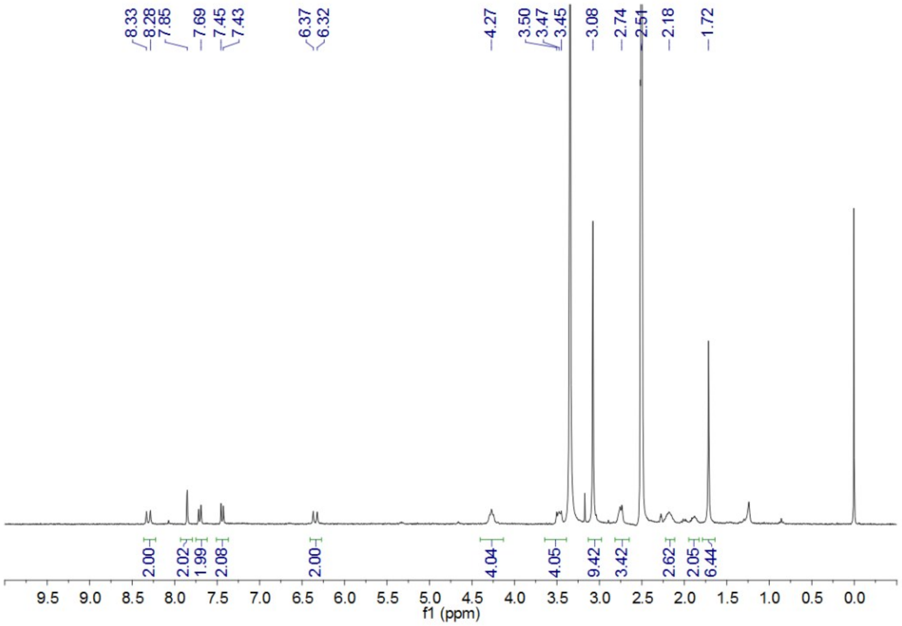

Figure S19. ^{1}H NMR of ZWCY in D_2O

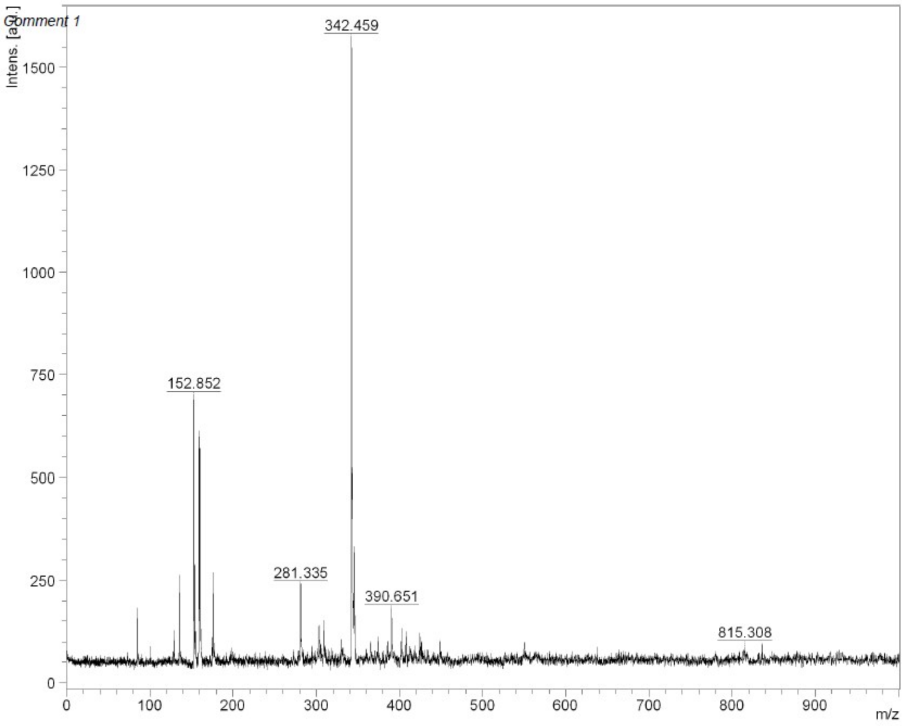

Figure S20. Mass spectra of ZWCY

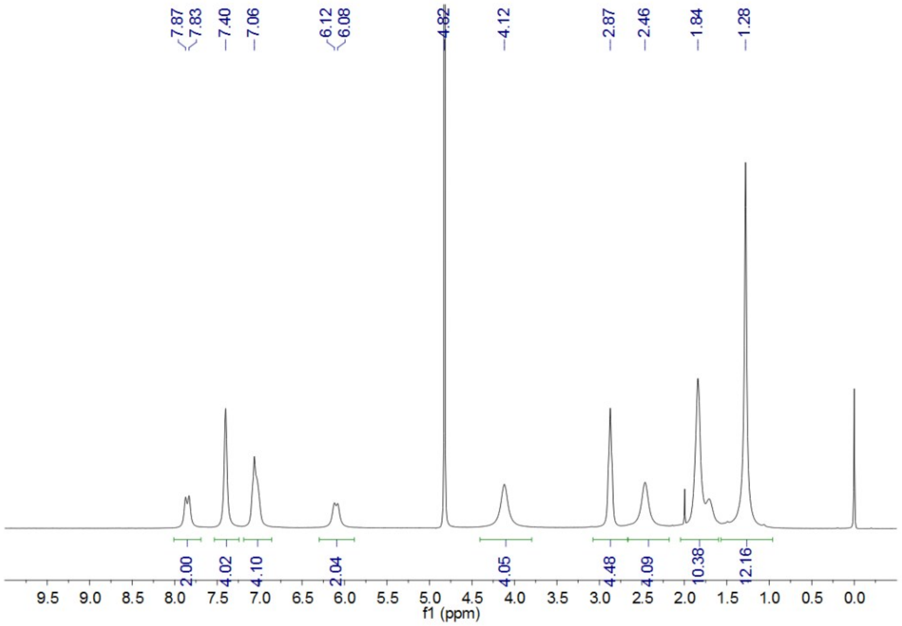

Figure S21. ^{1}H NMR of ANCY in D_2O

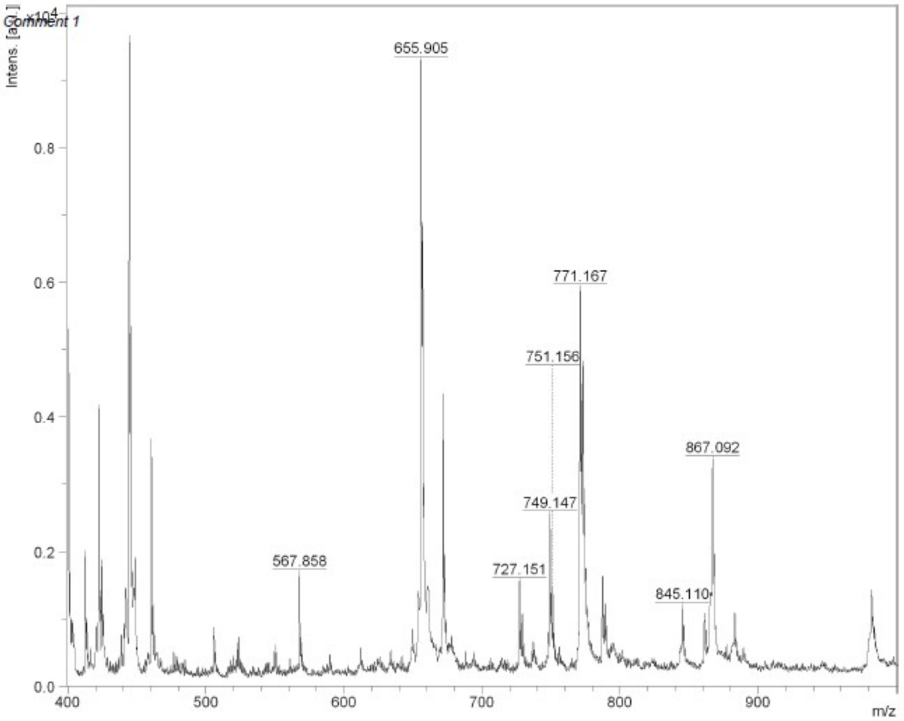

Figure S22. Mass spectra of ANCY

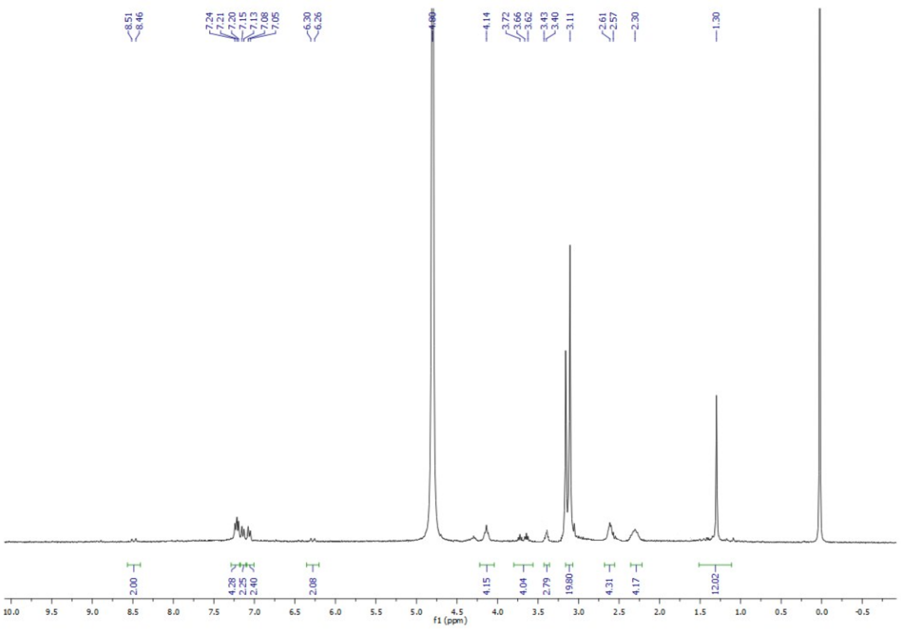

Figure S23. ^{1}H NMR of CACY in D_2O

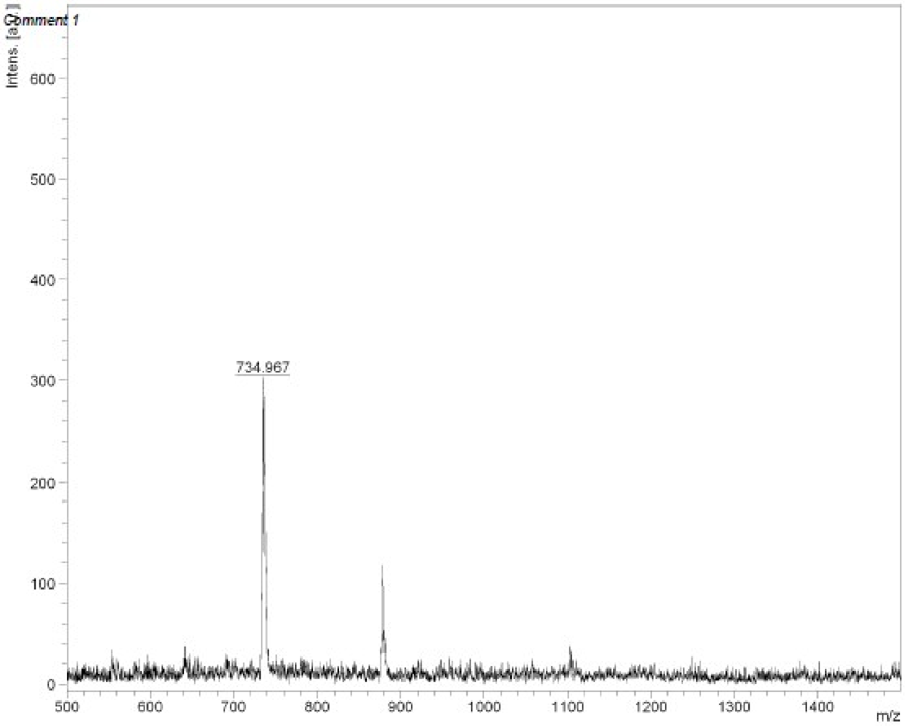

Figure S24. Mass spectra of CACY

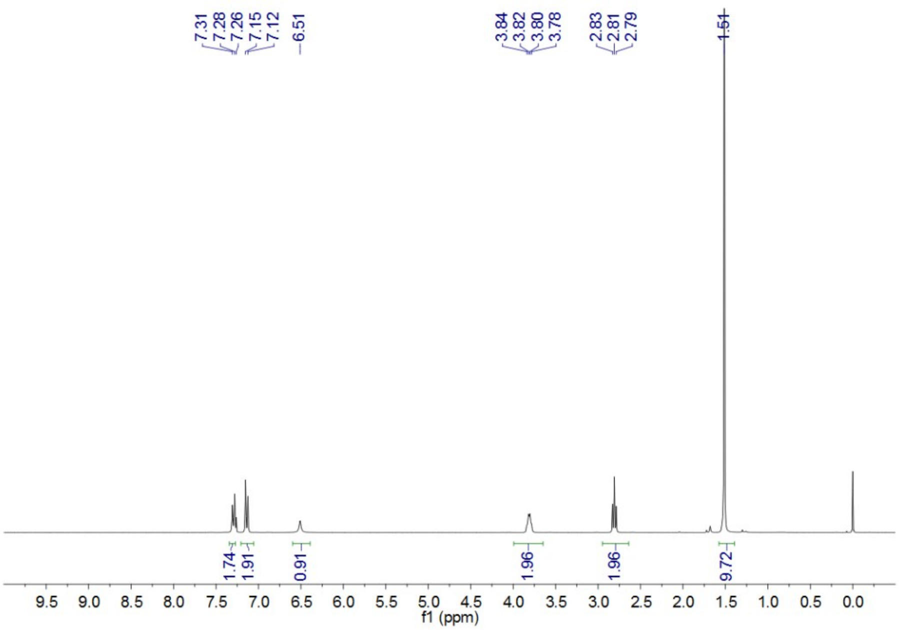

Figure S25. ^{1}H **NMR of Compound 9 in** $CDCl_3$

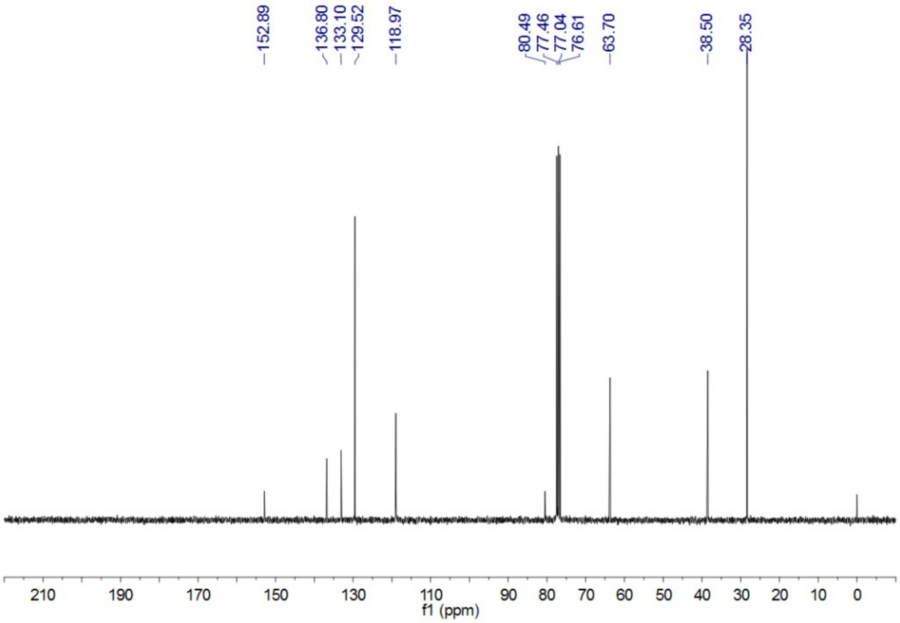

Figure S26. ^{13}C **NMR of Compound 9 in** $CDCl_3$

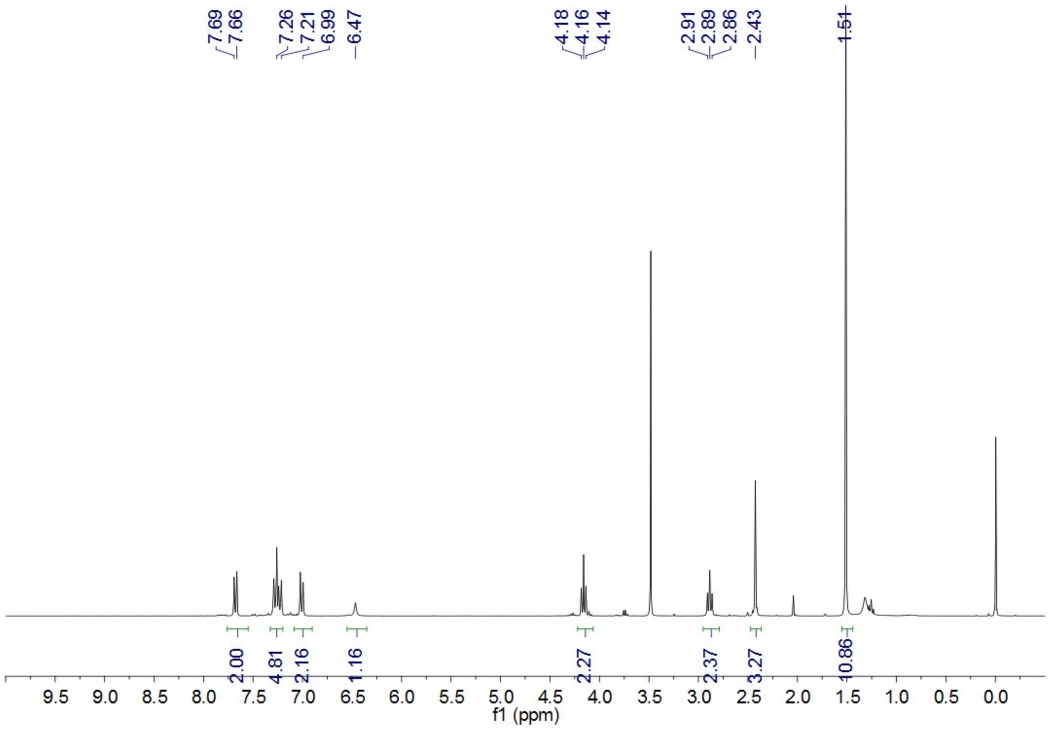

Figure S27. ^{1}H NMR of Compound 10 in $CDCl_3$

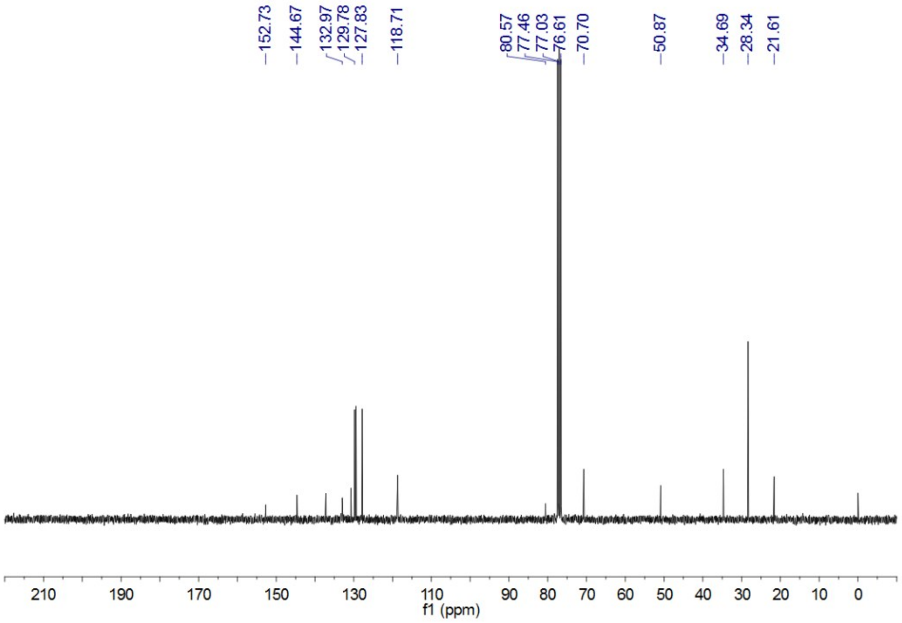

Figure S28. ^{13}C NMR of Compound 10 in $CDCl_3$

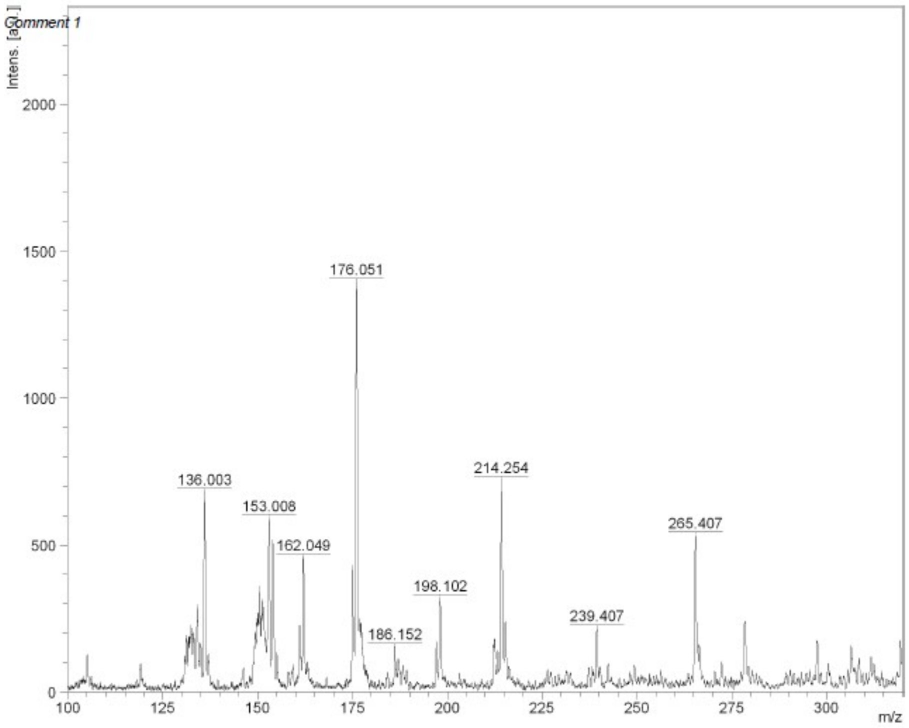

Figure S29. Mass spectra of 4-(2-azidoethyl) aniline

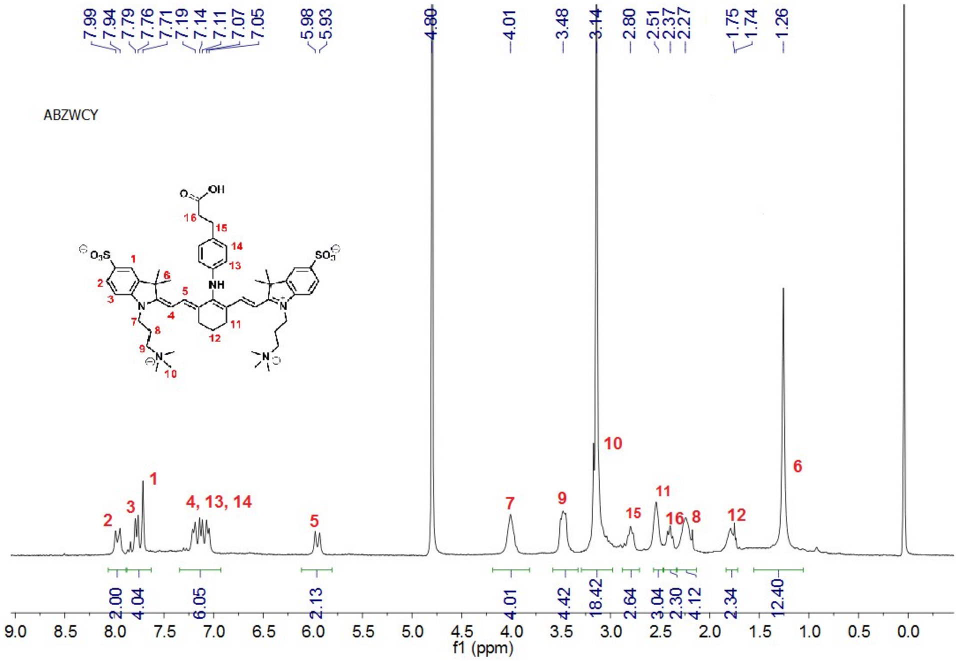

Figure S30. ^{1}H NMR of ABZWCY in D_2O

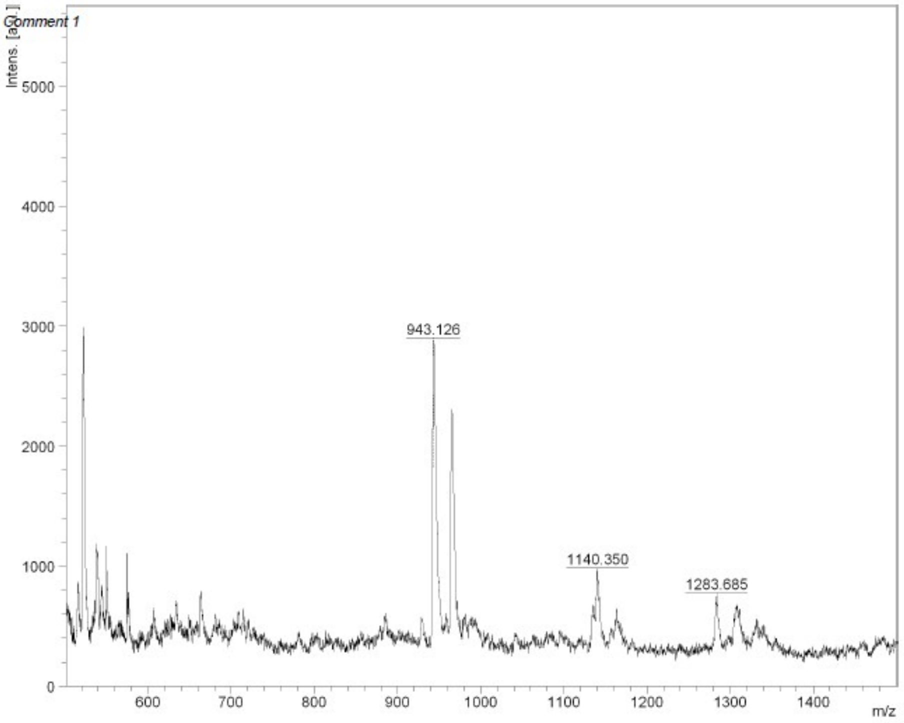

Figure S31. Mass spectra of ABZWCY

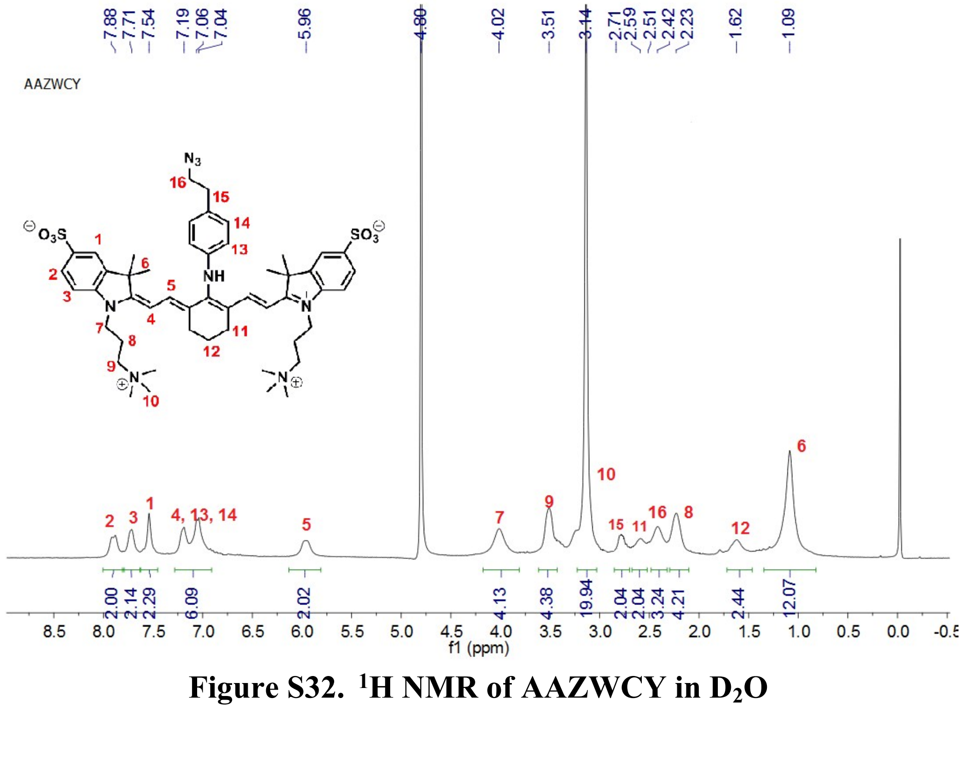

Figure S32. ^{1}H NMR of AAZWCY in D_2O

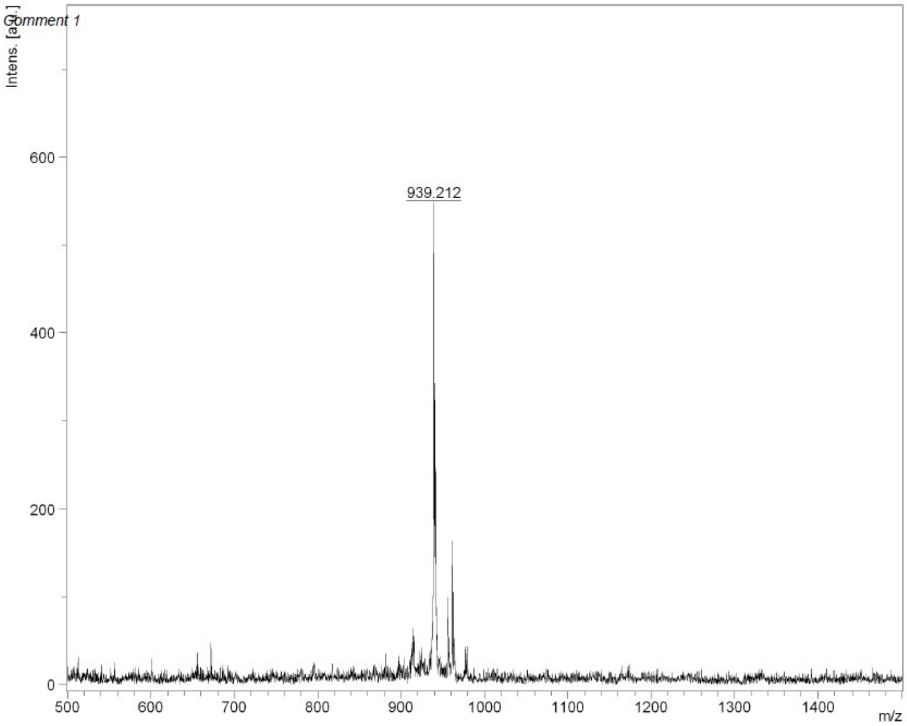

Figure S33. Mass spectra of AAZWCY

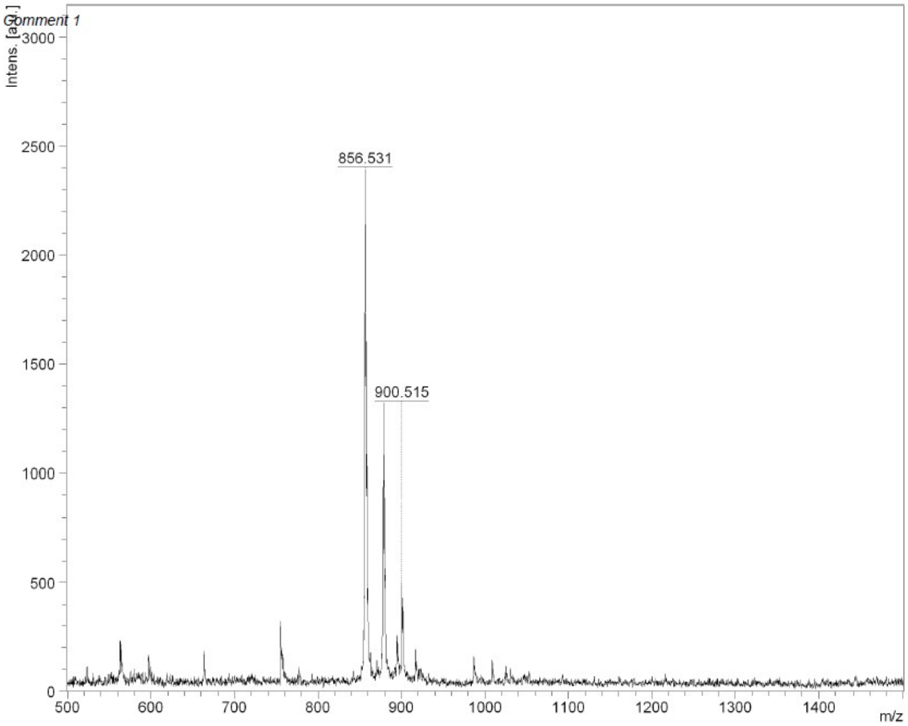

Figure S34. Mass spectra of ABANCY

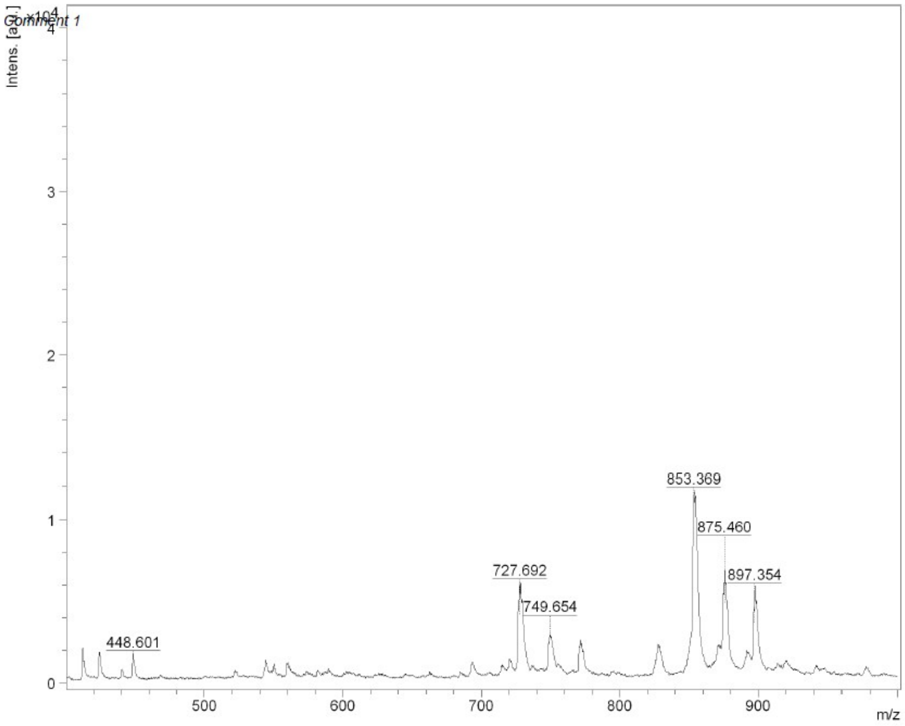

Figure S35. Mass spectra of AAANCY

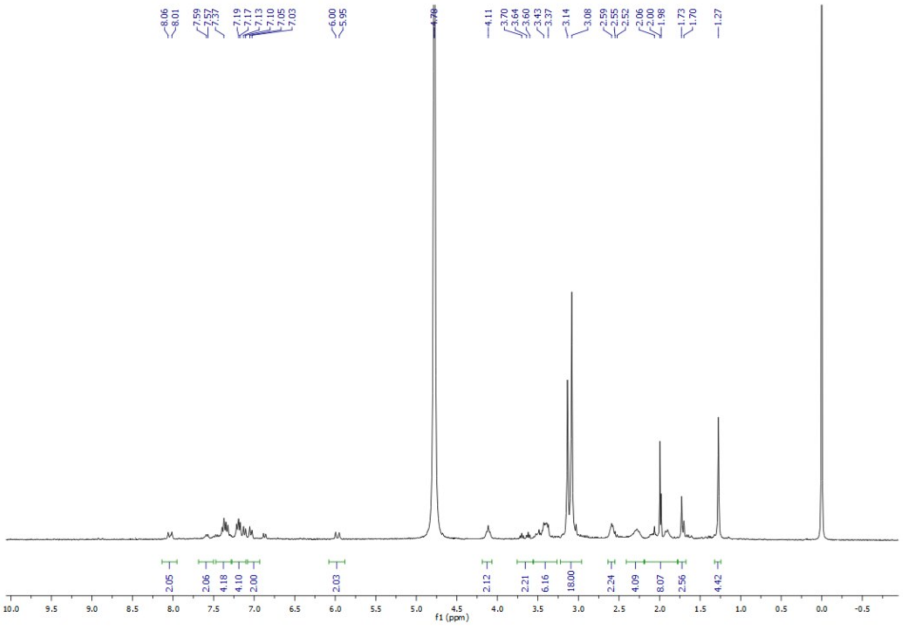

Figure S36. ^{1}H NMR of ABCACY in D_2O

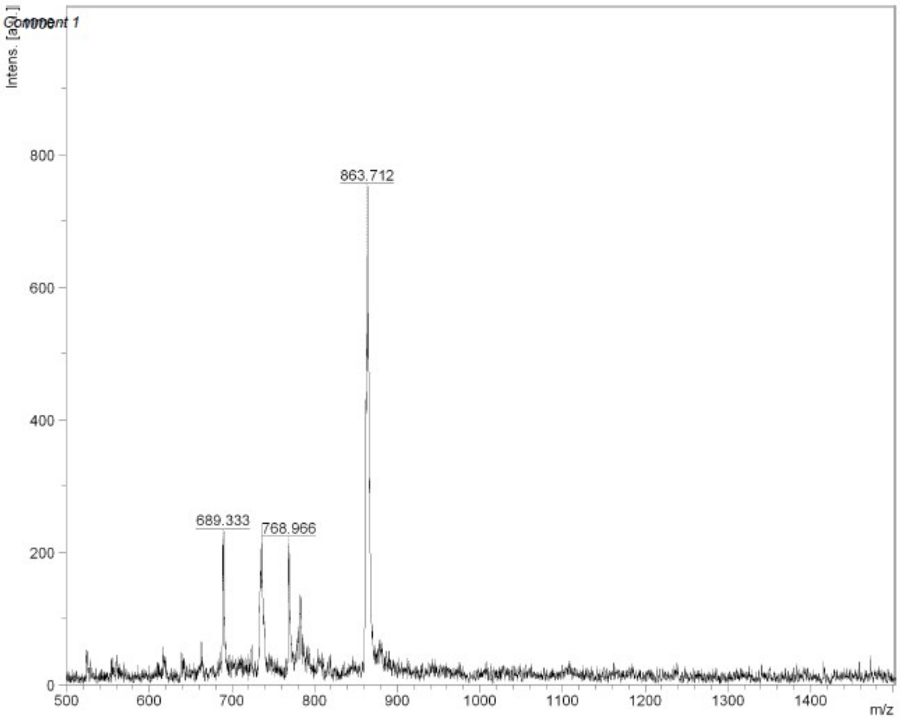

Figure S37. Mass spectra of ABCACY

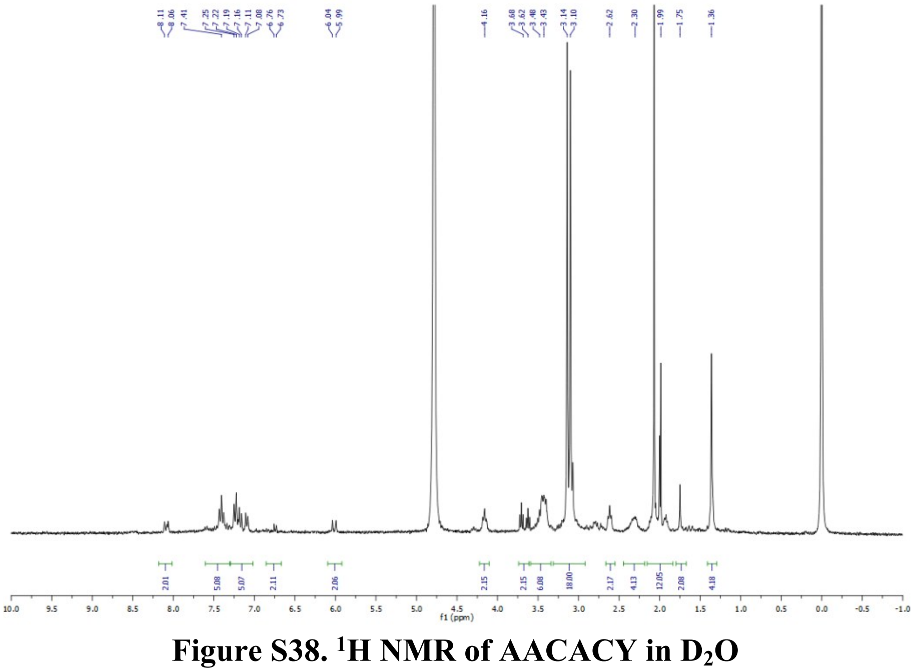

Figure S38. ^{1}H NMR of AACACY in D_2O

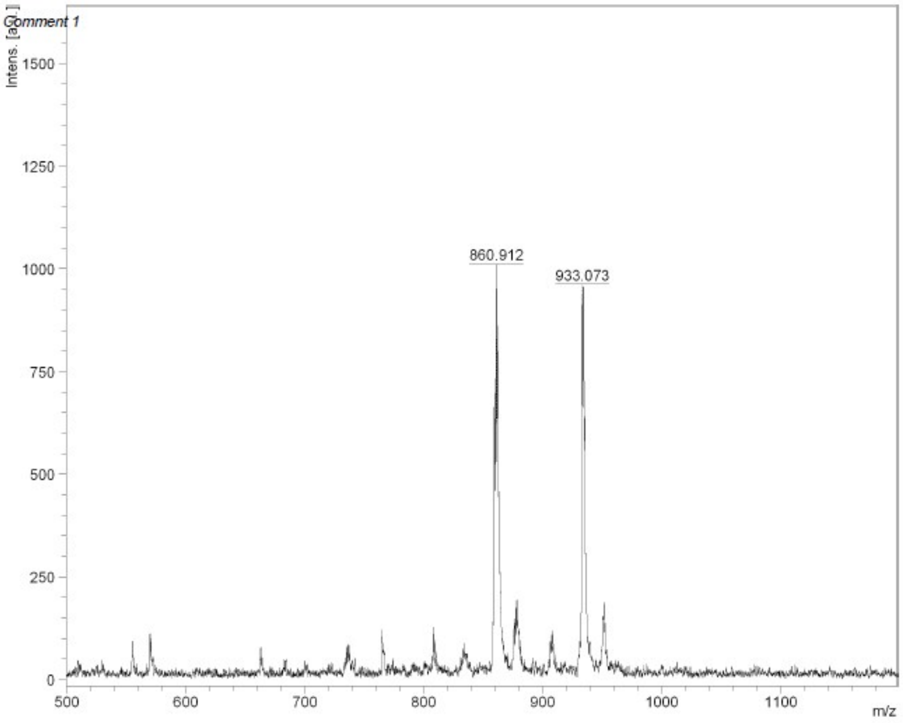

Figure S39. Mass spectra of AACACY

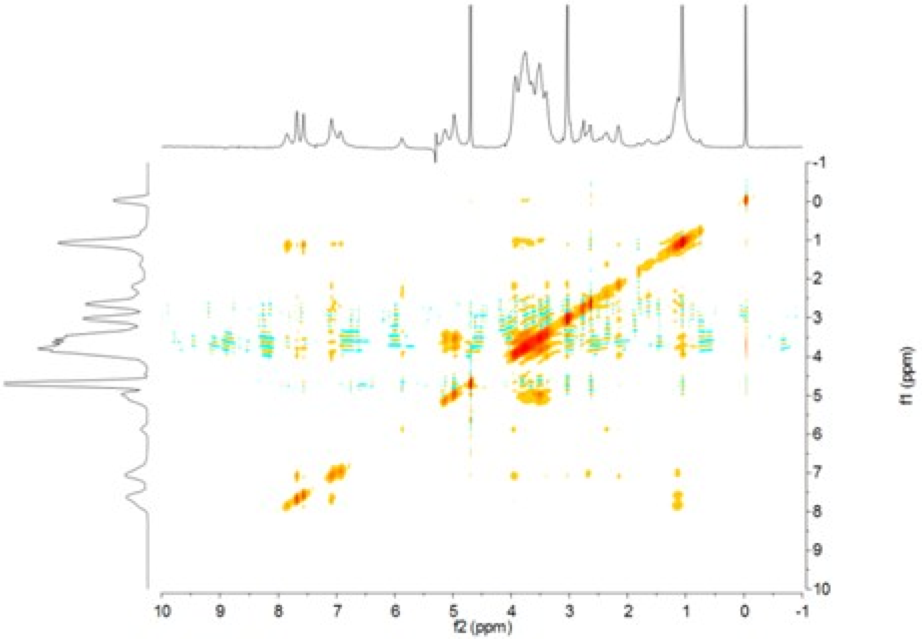

Figure S40. 2D-NOESY spectra of ABZWCY-HPβCD in D_2O at 25°C with a mixing time of 500 ms.

ABZWCY-HPβCD product contains labeled part (ABZWCY-HPβCD) and unlabeled part (HPβCD)

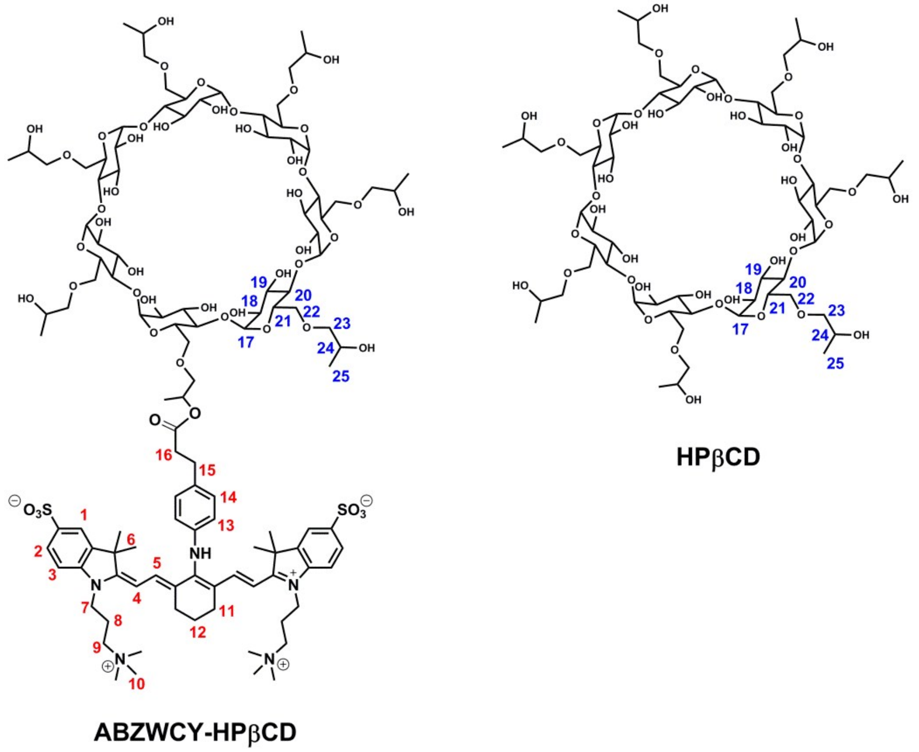

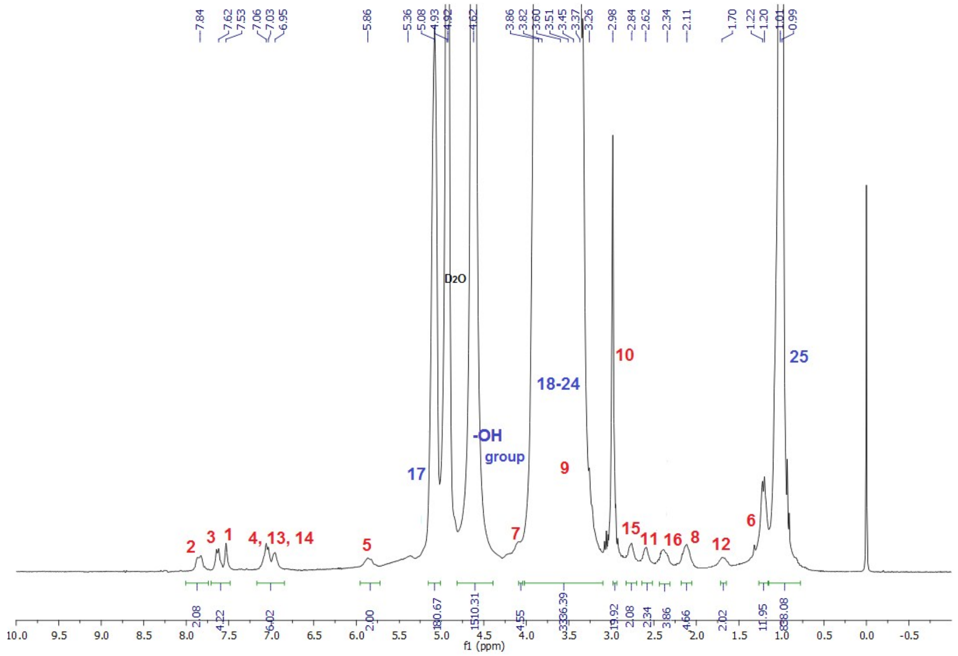

Figure S41. ^{1}H NMR of ABZWCY-HPβCD in D_2O

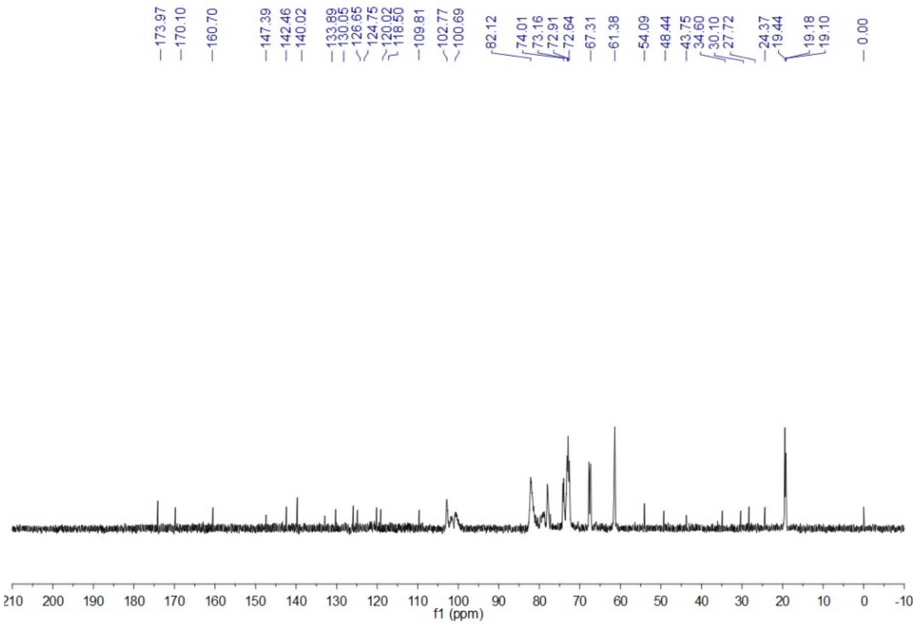

Figure S42. ^{13}C NMR of ABZWCY-HPβCD in D_2O

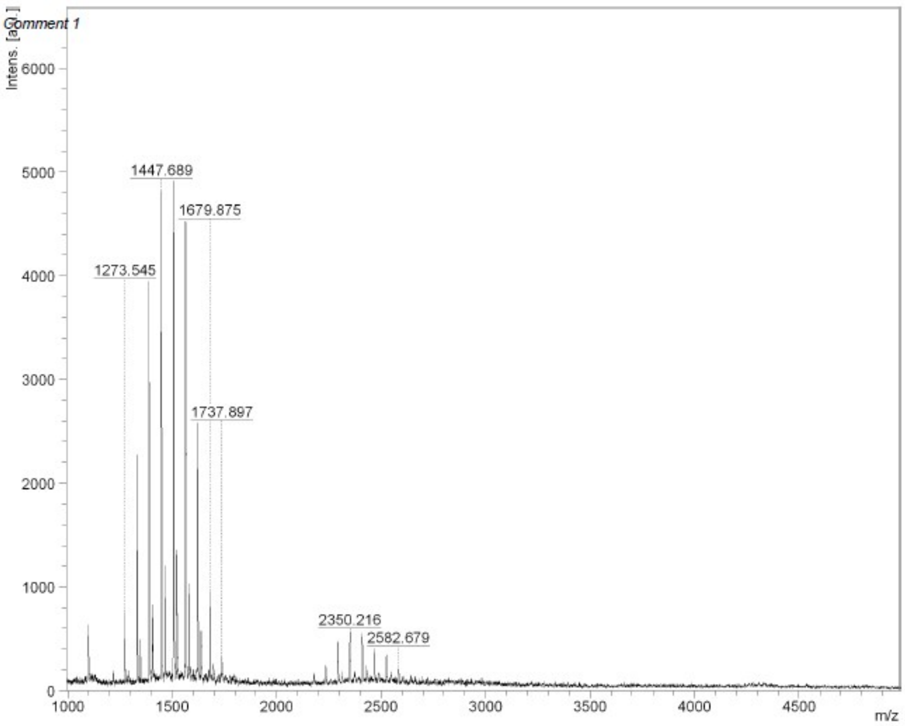

Figure S43. Mass spectra of ABZWCY-HPβCD

AAZWCY-HPβCD product contains labeled part (AAZWCY-HPβCD) and unlabeled part (HPβCD/propynyl-HPβCD)

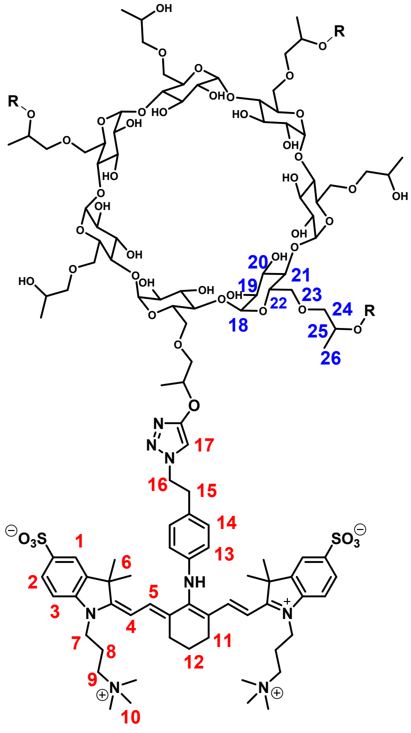

AAZWCY-HPβCD

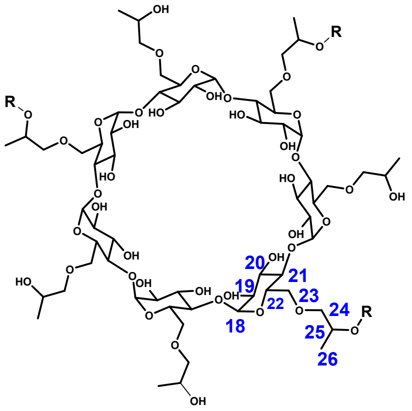

HPβCD or Propynyl-HPβCD

R = H or 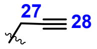

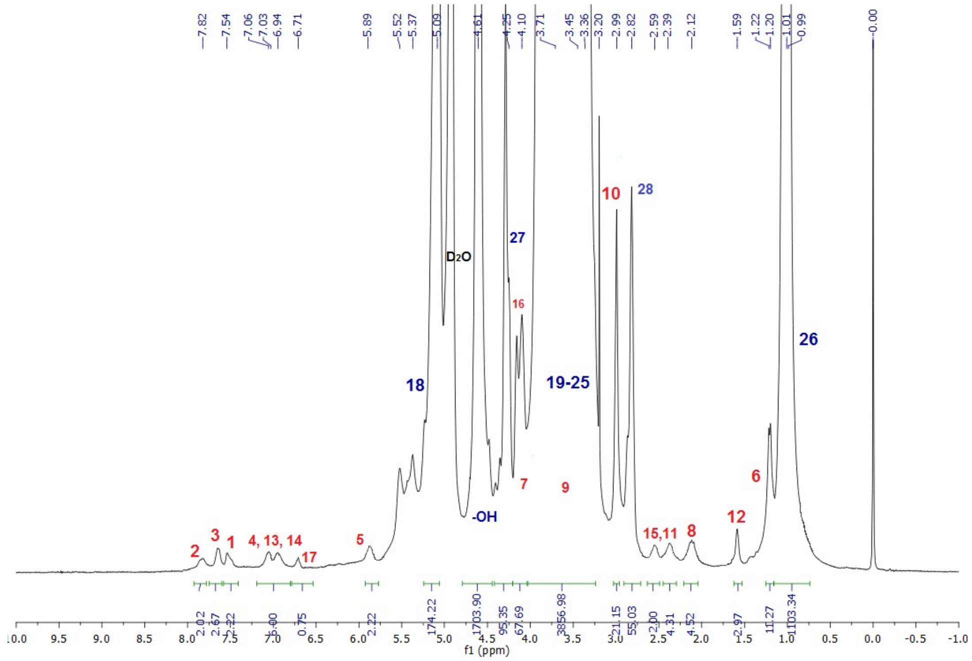

Figure S44. ^{1}H NMR of AAZWCY-HPβCD in D_2O

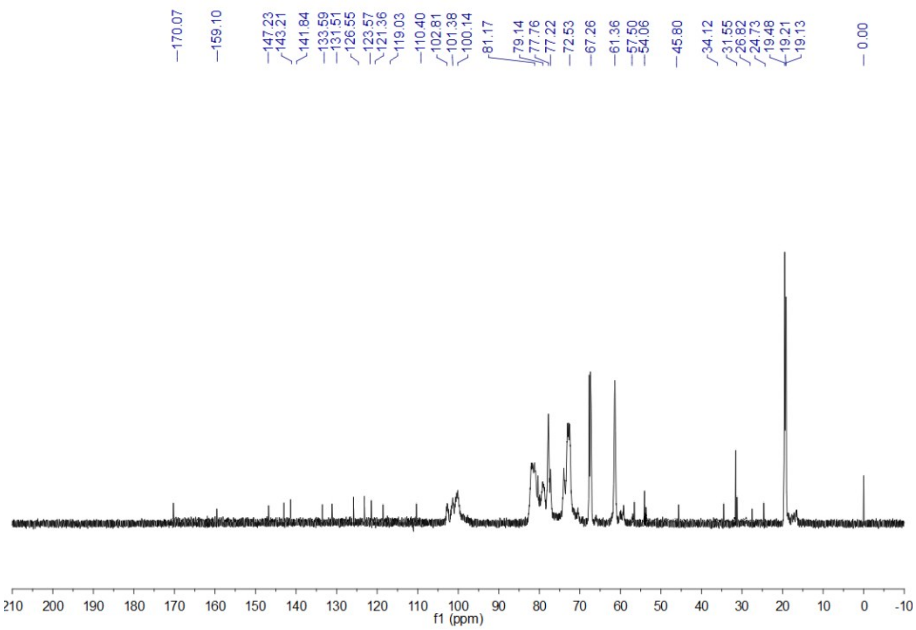

Figure S45. ^{13}C NMR of AAZWCY-HPβCD in D_2O

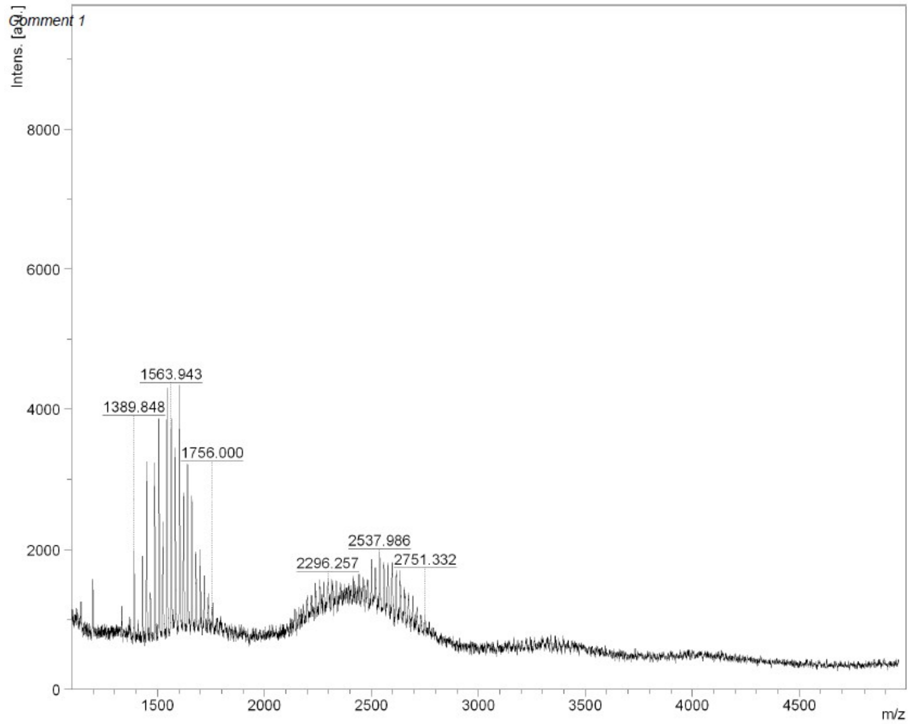

Figure S46. Mass spectra of AAZWCY-HPβCD

ABANCY-HPβCD product contains labeled part (ABANCY-HPβCD) and unlabeled part (HPβCD)

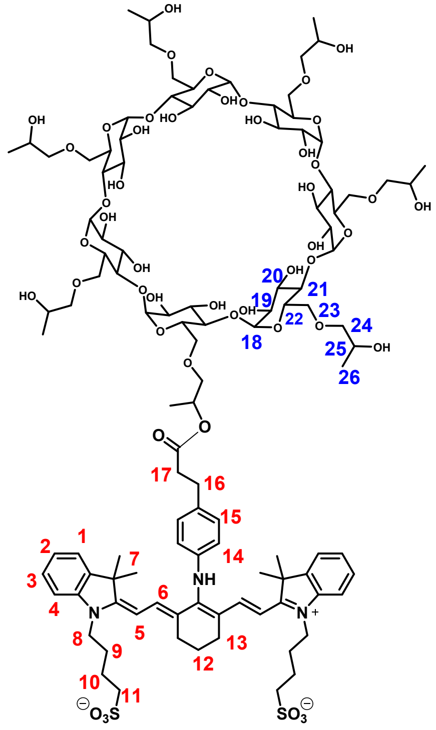

ABANCY-HPβCD

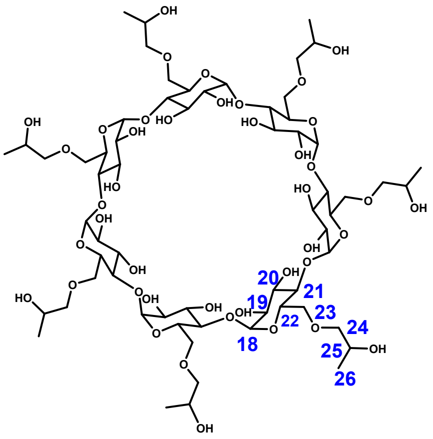

HPβCD

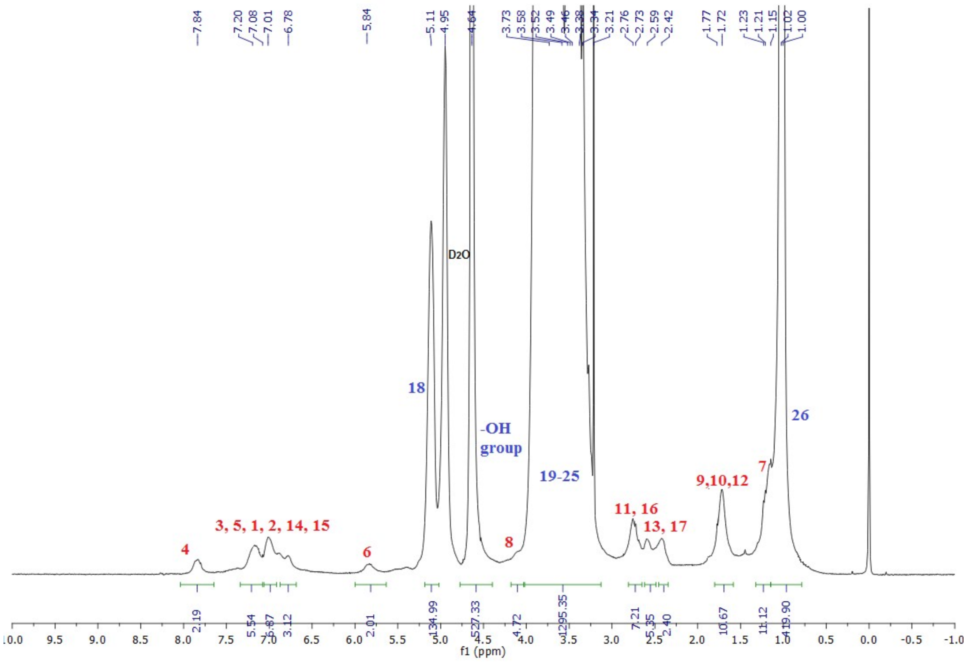

Figure S47. ^{1}H NMR of ABANCY-HPβCD in D_2O

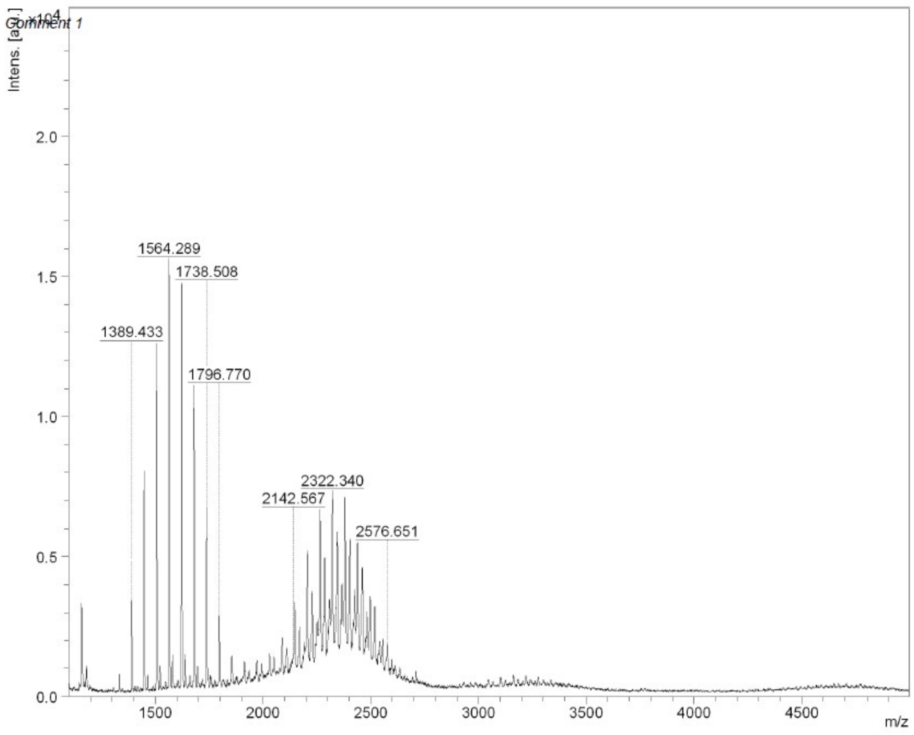

Figure S48. Mass spectra of ABANCY-HPβCD

AAANCY-HPβCD product contains labeled part (AAANCY-HPβCD) and unlabeled part (HPβCD/propynyl-HPβCD)

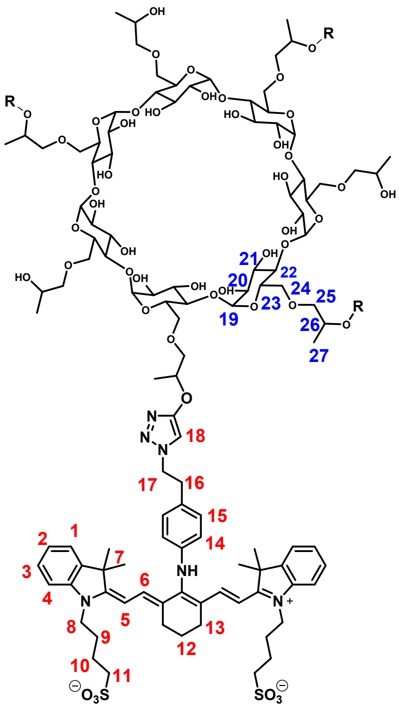

AAANCY-HPβCD

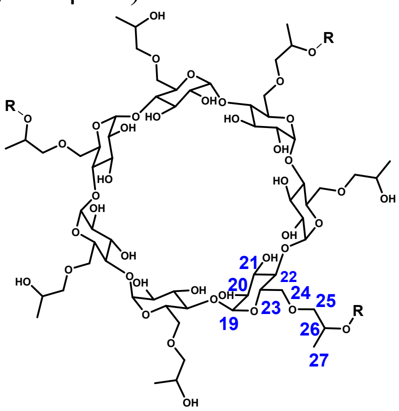

HPβCD or Propynyl-HPβCD

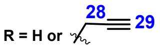

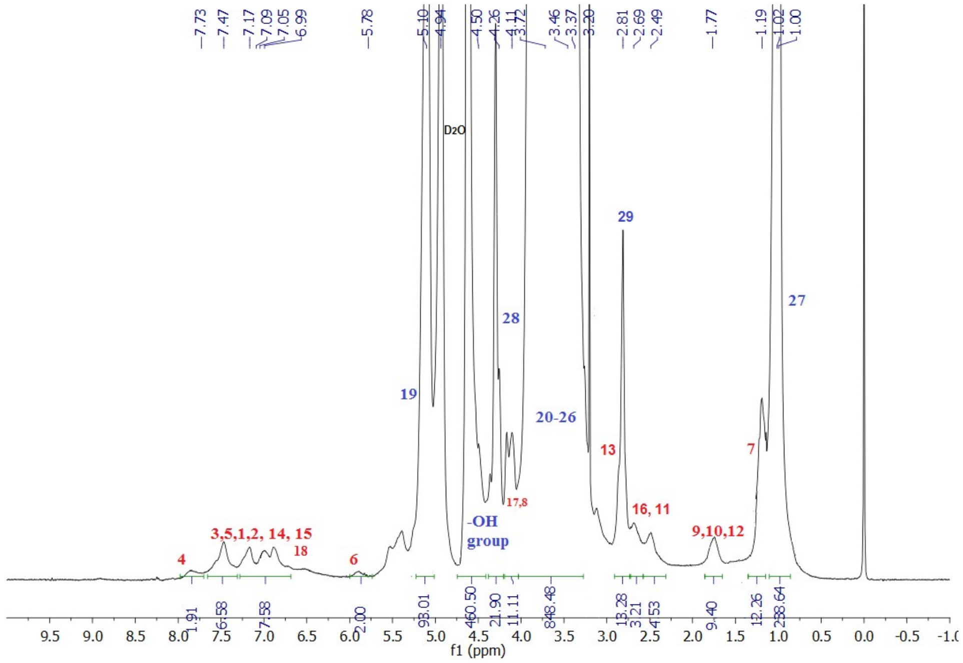

Figure S49. ^{1}H NMR of AAANCY-HPβCD in D_2O

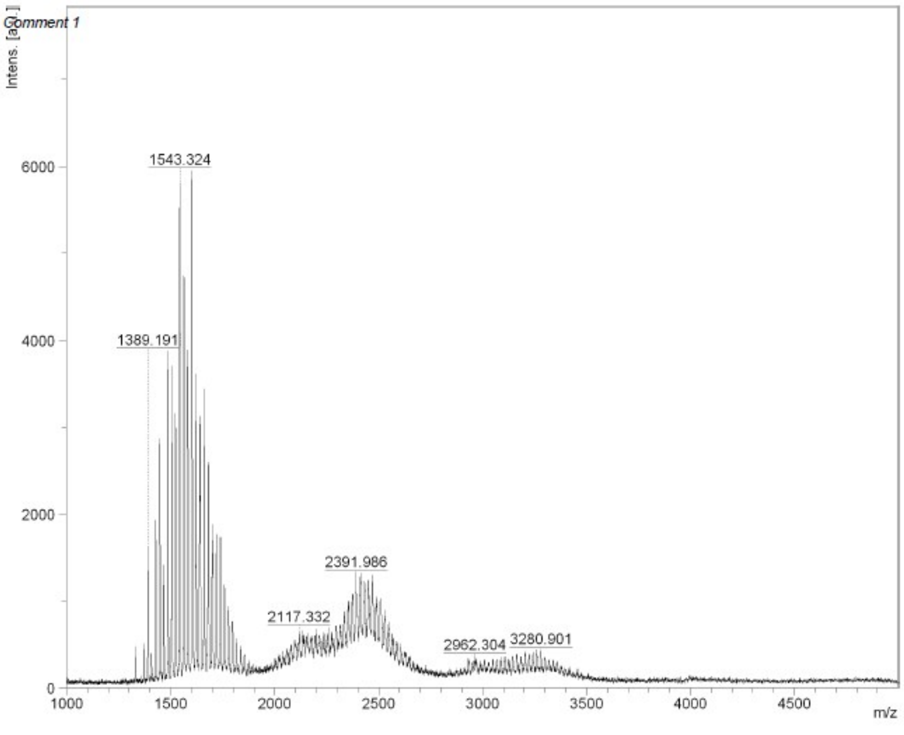

Figure S50. Mass spectra of AAANCY-HPβCD

ABCACY-HPβCD product contains labeled part (ABCACY-HPβCD) and unlabeled part (HPβCD)

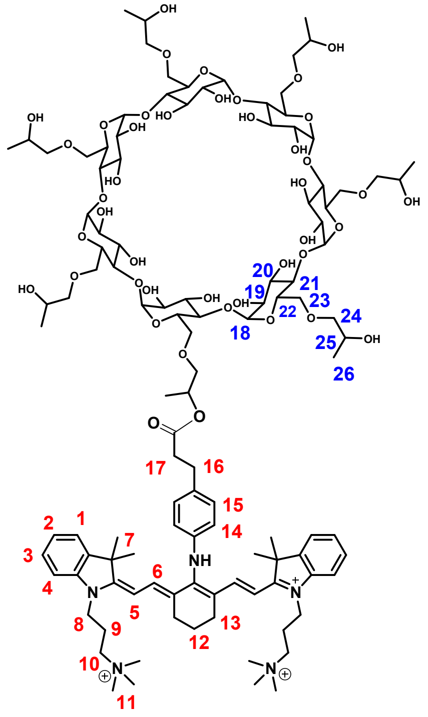

ABCACY-HPβCD

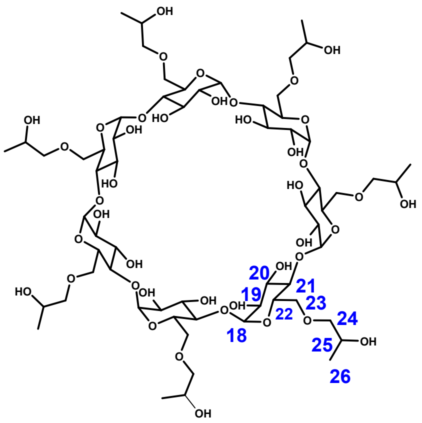

HPβCD

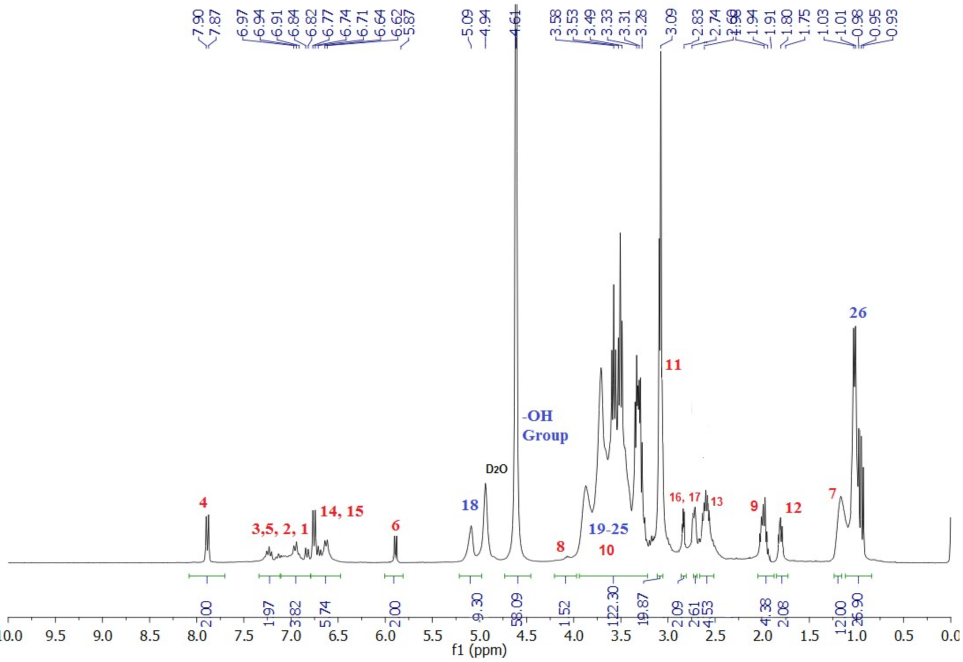

Figure S51. ^{13}C NMR of ABCACY-HPβCD in D_2O

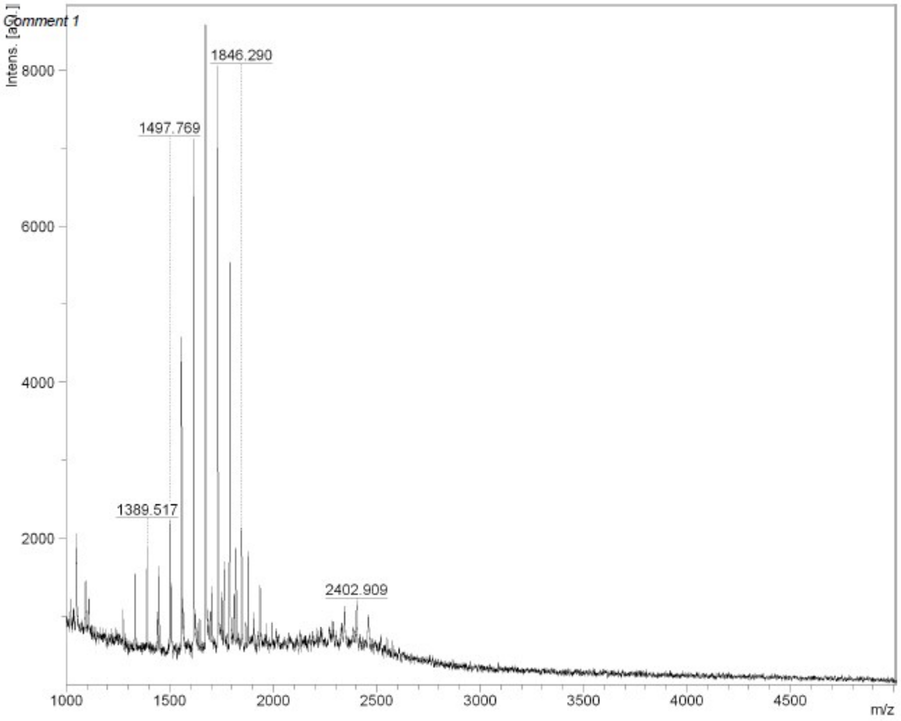

Figure S52. Mass spectra of ABCACY-HPβCD

AACACY-HPβCD product contains labeled part (AACACY-HPβCD) and unlabeled part (HPβCD/propynyl-HPβCD)

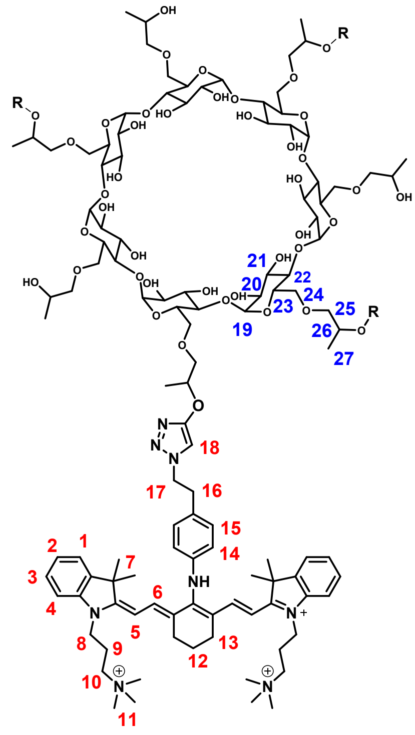

AACACY-HPβCD

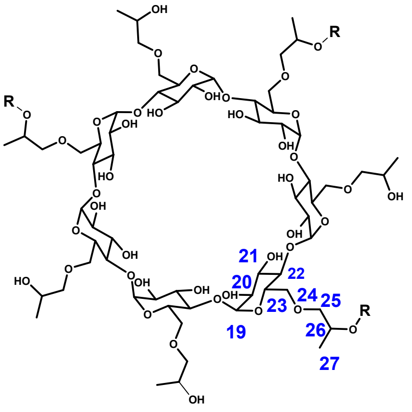

HPβCD or Propynyl-HPβCD

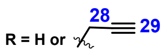

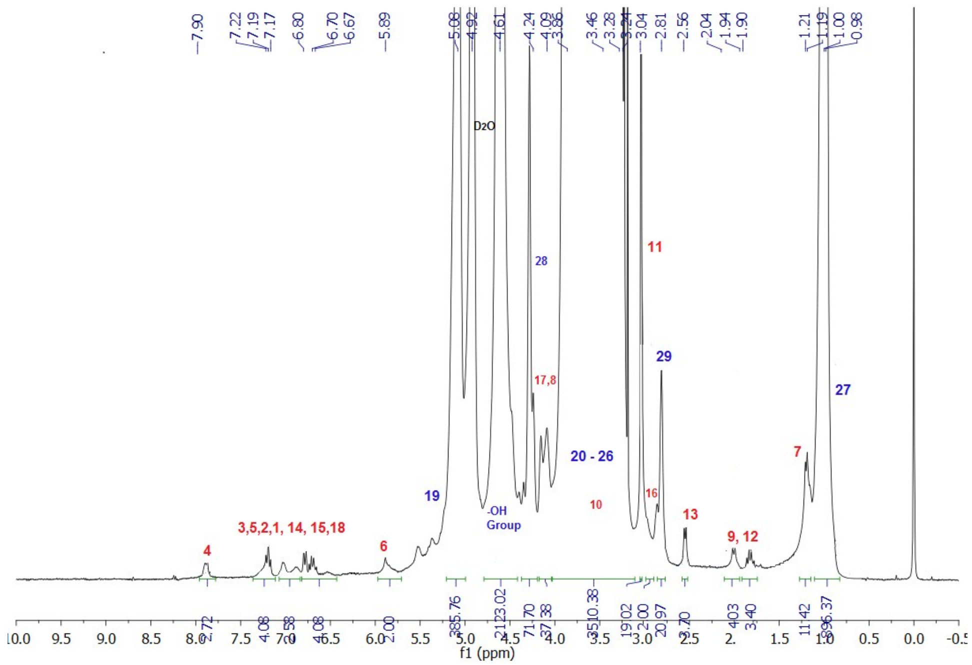

Figure S53. ^{1}H NMR of AACACY-HPβCD in D_2O

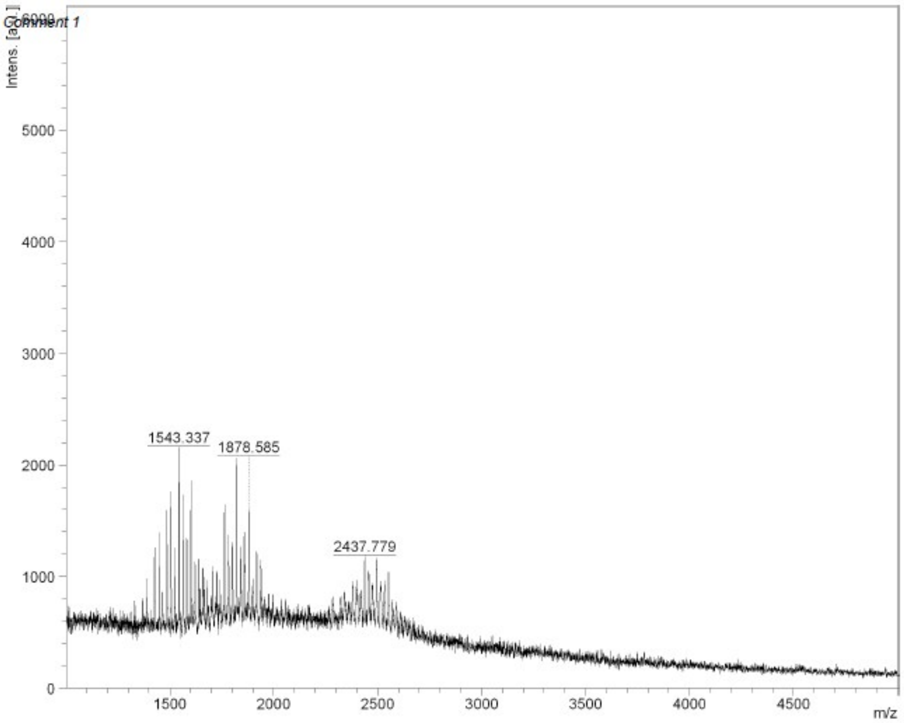

Figure S54. Mass spectra of AACACY-HPβCD

Reference:

1. (a) H. S. Choi, K. Nasr, S. Alyabyev, D. Feith, J. H. Lee, S. H. Kim, Y. Ashitate, H. Hyun, G. Patonay, L. Strekowski, M. Henary and J. V. Frangioni, *Angew Chem Int Ed Engl,* 2011, **50,** 6258-6263. (b) M. V. Kvach, S. V. Gontarev, I. A. Prokhorenko, I. A. Stepanova, V. V. Shmanai and V. A. Korshun, *Russ. Chem. Bull. Int.,* 2006, **55,** 159-163. (c) A. Petrova, D. Darin, A. Ivanov, L. Moskvin, R. Ishimatsu, K. Nakano, T. Imato and A. Bulatov, *Talanta,* 2016, **159,** 300-306.
2. S. Vira, E. Mekhedov, G. Humphrey and P. S. *Blank, Anal Biochem,* 2010, **402,** 146-150.
3. L. Scarfe, A. Rak-Raszewska, S. Geraci, D. Darssan, J. Sharkey, J. Huang, N. C. Burton, D. Mason, P. Ranjzad, S. Kenny, N. Gretz, R. Levy, B. Kevin Park, M. Garcia-Finana, A. S. Woolf, P. Murray and B. Wilm, *Sci. Rep.,* 2015, **5,** 13601.
4. K. Licha, B. Riefke, V. Ntziachristos, A. Becker, B. Chance and W. Semmler, *Photochem Photobiol,* 2000, **72,** 392-398.
5. F. M. Hamann, R. Brehm, J. Pauli, M. Grabolle, W. Frank, W. A. Kaiser, D. Fischer, U. Resch-Genger and I. Hilger, *Mol Imaging,* 2011, **10,** 258-269.
6. D. Schock-Kusch, Q. Xie, Y. hulhevich, J. Hesser, D. Stsepankou, M. Sadick, S. Koenig, F. Hoecklin, J. Pill and N. Gretz, *Kidney. Int.,* 2011, **79,** 1254-1258.
7. A. Shmarlouski, Y. Shulhevich, S. Geraci, J. Friedemann, N. Gretz, S. Neudecker, J. Hesser and D. Stsepankou, *Biomed Signal Process Control,* 2014, **14,** 30-41.
8. A. Schreiber, Y. Shulhevich, S. Geraci, J. Hesser, D. Stsepankou, S. Neudecker, S. Koenig, R. Heinrich, F. Hoecklin, J. Pill, J. Friedemann, F. Schweda, N. Gretz and D. Schock-Kusch, *Am J Physiol Renal Physiol.,* 2012, **303,** F783-788.
9. D. Schock-Kusch, M. Sadick, N. Henninger, B. Kraenzlin, G. Claus, H. M. Kloetzer, C. Weiss, J. Pill and N. Gretz, *Nephrol Dial Transplant.,* 2009, **24,** 2997-3001.
10. (a) E. C. Hopmans, *J. Agric. Food Chem.,* 1997, **45,** 2618-2625. (b) J. P. Danaceau, Thesis: A Simplified, Mixed-Mode Sample Preparation Strategy for Urinary Forensic Toxicology Screening by UPLC-MS/MS.
11. (a) S. Hoffmann, D. Podlich, B.Hähnel, W. Kriz and N. Gretz, *J. Am. Soc. Nephrol.,* 2004, **15,** 1475-1487. (b) H.H. Hsu, S. Hoffmann, N. Endlich, A. Velic, A. Schwab, T. Weide, E. Schlatter and H. Pavenstädt, *J. Mol. Med.,* 2008, **86,** 1379-1394.
